# Phase‐separated foci of EML4‐ALK facilitate signalling and depend upon an active kinase conformation

**DOI:** 10.15252/embr.202153693

**Published:** 2021-10-18

**Authors:** Josephina Sampson, Mark W Richards, Jene Choi, Andrew M Fry, Richard Bayliss

**Affiliations:** ^1^ School of Molecular and Cellular Biology Astbury Centre for Structural Molecular Biology Faculty of Biological Sciences University of Leeds Leeds UK; ^2^ Department of Pathology Asan Medical Center University of Ulsan College of Medicine Seoul Korea; ^3^ Department of Molecular and Cell Biology University of Leicester Leicester UK

**Keywords:** cancer, NSCLC, phase separation, signalling, tyrosine kinase inhibitors, Cancer, Signal Transduction, Structural Biology

## Abstract

Variants of the oncogenic EML4‐ALK fusion protein contain a similar region of ALK encompassing the kinase domain, but different portions of EML4. Here, we show that EML4‐ALK V1 and V3 proteins form cytoplasmic foci that contain components of the MAPK, PLCγ and PI3K signalling pathways. The ALK inhibitors ceritinib and lorlatinib dissolve these foci and EML4‐ALK V3 but not V1 protein re‐localises to microtubules, an effect recapitulated in a catalytically inactive EML4‐ALK mutant. Mutations that promote a constitutively active ALK stabilise the cytoplasmic foci even in the presence of these inhibitors. In contrast, the inhibitor alectinib increases foci formation of both wild‐type and catalytically inactive EML4‐ALK V3 proteins, but not a Lys‐Glu salt bridge mutant. We propose that EML4‐ALK foci formation occurs as a result of transient association of stable EML4‐ALK trimers mediated through an active conformation of the ALK kinase domain. Our results demonstrate the formation of EML4‐ALK cytoplasmic foci that orchestrate oncogenic signalling and reveal that their assembly depends upon the conformational state of the catalytic domain and can be differentially modulated by structurally divergent ALK inhibitors.

## Graphical abstract

EML4‐ALK fusion proteins phase separate into foci that contain oncogenic signaling factors, through a mechanism that depends on an active ALK kinase conformation. Variants have distinct dynamics determined by a specific region of EML4.

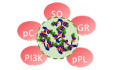

## Introduction

Lung cancer is the most common cancer globally and, despite advances in targeted treatment, the 10‐year survival rate of patients with lung cancer is only 9% in the UK (Sabir *et al*, 2017). More than 80% of lung cancers are classified as non‐small‐cell lung cancer (NSCLC), of which most are adenocarcinomas. While activating mutations in RAS or epidermal growth factor receptor (EGFR) are the most common drivers of NSCLC, oncogenic fusions created by chromosome translocations are also frequent driver events (Yuan *et al*, 2019). These frequently encode proteins in which the kinase domain of a receptor tyrosine kinase (RTK) is constitutively activated by translational fusion to a self‐associating region of another protein. One common example is the fusion of anaplastic lymphoma kinase (ALK) and echinoderm microtubule‐associated protein‐like 4 (EML4), found in ˜ 2–9% of NSCLC patients (Soda *et al*, 2007).

To date, at least 15 EML4‐ALK variants have been identified all containing the cytoplasmic tyrosine kinase domain of ALK. The variants differ in the point of fusion within the EML4 gene although all retain the N‐terminal region of EML4 that encodes its coiled‐coil trimerisation domain (TD) through which the fusion proteins achieve self‐association and constitutive ALK activation (Soda *et al*, 2007; Richards *et al*, 2014). The predominant EML4‐ALK variants identified in patients are variants 1 and 3 (V1 and V3), accounting for around 33% and 29% of cases, respectively. Structurally, EML4‐ALK V3 contains the TD and an unstructured, basic region that mediates microtubule association of EML4, but lacks its entire C‐terminal tandem atypical β‐propeller (TAPE) domain, whereas V1 also retains a large, albeit incomplete, part of the TAPE domain, the presence of which confers dependence on Hsp90 for stability (Richards *et al*, 2014).

Since EML4‐ALK fusions were identified in NSCLC, potent tyrosine kinase inhibitors (TKI) against ALK have been developed and used as a first‐line treatment for those patients. First‐ and second‐generation inhibitors, crizotinib, ceritinib and alectinib, are currently used as first‐line treatment for ALK‐positive NSCLC patients, while lorlatinib, a third‐generation inhibitor, is approved for a second‐ or third‐line treatment after crizotinib, ceritinib or alectinib in ALK‐positive metastatic NSCLC (Solomon *et al*, 2014, 2018). NSCLC harbouring different EML4‐ALK variants exhibit different responses to ALK inhibitors. NSCLC patients identified with EML4‐ALK V1 respond to crizotinib and have increased progression‐free survival (PFS) after treatment (Shaw *et al*, 2011; Yoshida *et al*, 2016). However, EML4‐ALK V3‐positive NSCLC patients demonstrate a higher metastatic spread and increased aggressiveness of the disease, while *in vitro* NSCLC cells harbouring EML4‐ALK V3 exhibit resistance to various ALK inhibitors (Woo *et al*, 2017; Christopoulos *et al*, 2018; O'Regan *et al*, 2020).

The cytoplasm is no longer considered a homogenous solution but rather to contain unevenly distributed protein and RNA molecules forming dynamic assemblies through transient molecular interactions. This behaviour sometimes causes molecules associated with these dynamic assemblies to partition from the bulk cytoplasm into droplets through a process called liquid–liquid phase separation (LLPS) (Hyman *et al*, 2014; Boeynaems *et al*, 2018). Several studies have reported proteins forming foci within the cytoplasm, such as Tau and polo‐like kinase 4 (PLK4) (Wegmann *et al*, 2018; Park *et al*, 2019). Indeed, liquid‐like foci have long been described in numerous papers as membraneless organelles, such as the P granules in *C. elegans* embryos (Brangwynne *et al*, 2009). The major characteristics of LLPS foci are that they: (i) fuse after touching and revert into a spherical shape; (ii) deform, diffuse and exchange material with the cytoplasm; (iii) adopt a spherical shape that is driven by surface tension; and (iv) recover rapidly through internal rearrangement and cytoplasmic exchange when photobleached (Hyman *et al*, 2014). Recently, Tulpule and colleagues have observed RTK fusion proteins, including EML4‐ALK V1 and V3, forming membraneless cytoplasmic granules that act as centres for the organisation and activation of RAS and other downstream signalling pathway components (Tulpule *et al*, 2021). Intriguingly, the granules are disrupted by a catalytically inactive mutant leading to the hypothesis that their formation is dependent on ALK activity (Tulpule *et al*, 2021). Despite the recent progress made to understand the functional role of EML4‐ALK foci in oncogenic signalling, the molecular interactions that underpin their formation are unknown.

In the present study, we demonstrate that EML4‐ALK V1 and V3 proteins partition into cytoplasmic foci in a manner dependent on the active conformation, rather than catalytic activity itself, of the ALK kinase domain. We confirm that the V1 and V3 cytoplasmic foci are rich in protein–protein interactions and capture signalling proteins such as GRB2, SOS1, PLCγ2, PI3K and KIT, suggesting that they may act as hubs for activation of downstream pathways. Interestingly, while V3 foci exhibit typical LLPS characteristics, we found that V1 foci have a more solid‐like state due to the presence of a specific sub‐domain within the partial TAPE region of V1. We also reveal how the ALK inhibitors, ceritinib, alectinib and lorlatinib, differentially affect the localisation and behaviour of EML4‐ALK V1 and V3 inside the cell by dissolving or maintaining these cytoplasmic foci. Taken together, we propose a model to explain how EML4‐ALK foci formation is connected to the activation mechanism of ALK.

## Results

### EML4‐ALK V3 forms dynamic cytoplasmic foci while V1 foci are static

**Figure 1:**
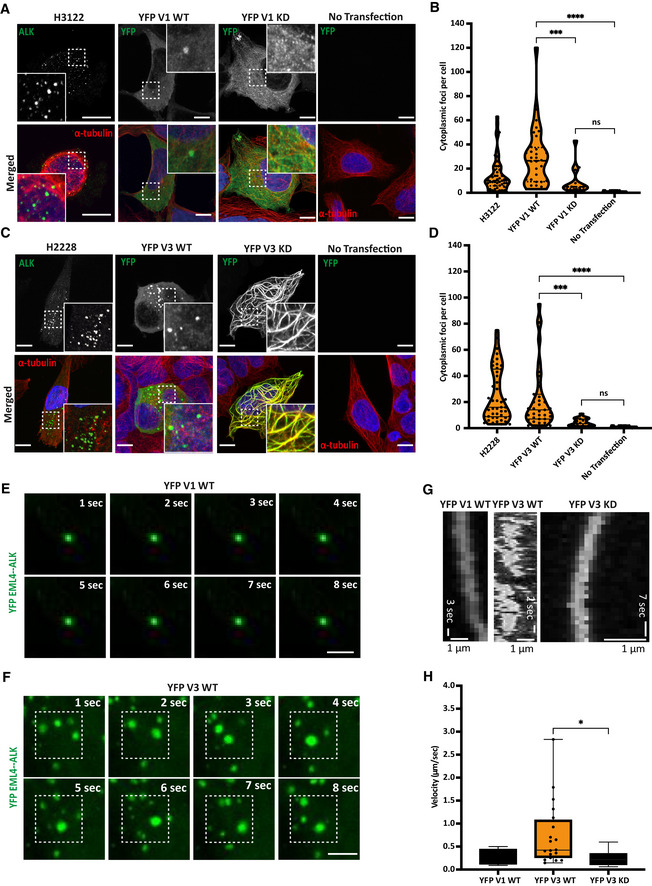
EML4‐ALK V1 and V3 activation induces the formation of cytoplasmic foci H3122 cells expressing endogenous EML4‐ALK V1 and HEK293 transfected with YFP‐EML4‐ALK V1 WT and KD were stained for either anti‐ALK or anti‐GFP (green), anti‐α‐tubulin (red), and DAPI (blue). Scale bars, 10 μm; magnified views of a selected area are shown.Violin plots showing the number of EML4‐ALK V1 cytoplasmic foci per cell. Data represent 30–50 counts from three biological replicates. ***P < 0.001, ****P < 0.0001 in comparison with HEK293 YFP‐EML4‐ALK‐V1 KD by one‐way ANOVA.H2228 cells expressing endogenous EML4‐ALK V3 and HEK293 transfected with YFP‐EML4‐ALK V3 WT and KD were stained for either anti‐ALK or anti‐GFP (green), anti‐α‐tubulin (red) and DAPI (blue). Scale bars, 10 μm; magnified views of a selected area are shown.Violin plots showing the number of EML4‐ALK V3 cytoplasmic foci per cell. Data represent 30–50 counts from four biological replicates. ***P < 0.001, ****P < 0.0001 in comparison with HEK293 YFP‐EML4‐ALK‐V3 KD by one‐way ANOVA.Time‐lapse imaging of transfected HEK293 YFP‐EML4‐ALK V1 WT to observe the movement of cytoplasmic foci. Representative still images are shown of an area of cytoplasm containing YFP‐EML4‐ALK V1 WT and V3 WT foci at the times indicated. Scale bar, 1 μm.Time‐lapse imaging of transfected HEK293 YFP‐EML4‐ALK V3 WT to observe the movement of cytoplasmic foci. Representative still images are shown of an area of cytoplasm containing YFP‐EML4‐ALK V1 WT and V3 WT foci at the times indicated. Scale bar, 1 μm.Kymographs showing the movement of YFP‐EML4‐ALK V1 WT, YFP‐EML4‐ALK V3 WT and YFP‐EML4‐ALK V3 KD over the duration of the observation period. Scale bars; 1 μm horizontal; seconds vertical.Whisker plot showing the calculated velocity of single events in the kymograph. Data represent 20 counts from 10 kymographs. *n = *2. Error bar represents SD of two biological replicates. *P < 0.05 in comparison with YFP EML4‐ALK V3 KD by unpaired *t*‐test.

**Figure EV1:**
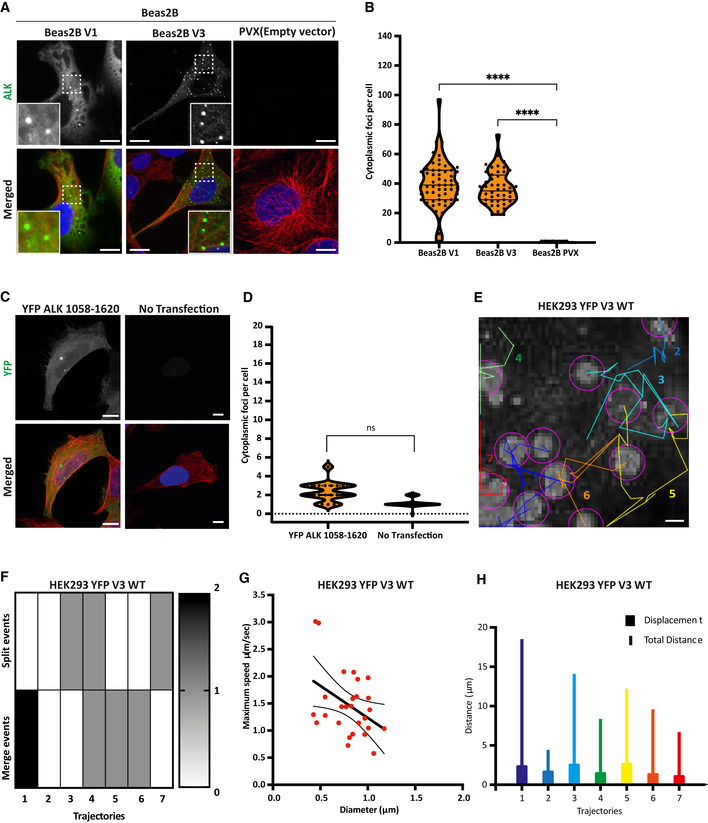
Beas2B inducible cells form cytoplasmic foci but not YFP‐ALK, and the tracking analysis of EML4‐ALK V3 foci Inducible Beas2B V1, V3 and PVX (empty vector) cells were stained with either anti‐ALK (green) or anti‐GFP (green), anti‐α‐tubulin (red) and DAPI (blue). Scale bars, 10 μm; magnified views of a selected area are shown.Violin plot showing the number of cytoplasmic foci per cell. Data represent 40–50 counts from three biological replicates. ****P < 0.0001 in comparison with Beas2B PVX (empty vector) by one‐way ANOVA.HEK293 cells were transfected with YFP‐ALK 1,058–1,620 for 48 h before fixation and staining with anti‐GFP (green), anti‐α‐tubulin (red) and DAPI (blue). Scale bars, 10 μm.Violin plot representing the number of cytoplasmic foci per cell. Data represent counts from at least 20 cells from two biological replicates. Not significant (ns) in comparison with YFP EML4‐ALK 1,058–1,620 by unpaired *t*‐test.Track classification of YFP‐EML4‐ALK V3 foci in the cytoplasm of HEK293. Duration of the movie was 12 s. Scale bar, 1 μm. Each coloured and numbered trajectory indicates the movement of an individual droplet.Heatmap representing the number of split and merge events in each trajectory.Plot of foci maximum speed (μm/s) versus foci diameter (μm). The best‐fit line shows correlation with 95% confidence interval.Plot displays the total distance (μm) and displacement (μm) of each trajectory. Colours and numbers relate to the tracks shown in (E).

Previous studies have reported distinct subcellular localisation of EML4‐ALK to cytoplasm or microtubules depending on the type of variant; however, the precise localisation of these proteins was not clearly defined (Hrustanovic *et al*, 2015; Richards *et al*, 2015). We therefore examined the subcellular localisation of EML4‐ALK V1 and V3 by fixed‐cell imaging in several cell lines: NSCLC patient‐derived cancer cell lines harbouring endogenous EML4‐ALK V1 (H3122) or EML4‐ALK V3 (H2228), a non‐transformed human lung epithelial cell line (Beas2B) expressing EML4‐ALK V1 or V3 in a doxycycline‐inducible manner and HEK293 cells overexpressing YFP‐EML4‐ALK V1 or V3. Fixed imaging analysis revealed approximately 15‐60 prominent cytoplasmic foci of EML4‐ALK V1 in each of the three different cell types (Figs 1A and B, and EV1A and B). Similarly, we identified 25–50 prominent foci of EML4‐ALK V3 in the cytoplasm of each different cell type (Figs 1C and D, and EV1A and B). In contrast, YFP‐ALK 1058–1620 (the region of ALK present in the EML4‐ALK fusions) showed very few (at most 5) distinct droplets in the cytoplasm upon expression in HEK293 cells (Fig EV1C and D). To test whether formation of EML4‐ALK V1 and V3 cytoplasmic foci requires catalytic activity of the ALK tyrosine kinase, we generated catalytically inactive (D1270N) versions of V1 and V3 and analysed their localisation by fixed imaging. Observation of transfected HEK293 cells revealed significantly fewer distinct EML4‐ALK V1 and V3 cytoplasmic foci with the kinase‐dead (KD) compared with wild‐type (WT) constructs (Fig 1A–D). Interestingly, the KD mutant of V3, but not V1, associated strongly with what appeared to be bundled microtubules (Fig 1A–D). Taken together, these data suggest that both EML4‐ALK variants form foci of concentrated protein in the cytoplasm in a process that requires ALK catalytic activity.

To explore the properties of the EML4‐ALK V1 and V3 cytoplasmic foci, we performed time‐lapse imaging of transfected YFP‐EML4‐ALK V1 and V3 HEK293 cells. Observation of YFP‐EML4‐ALK V1 WT transfected cells revealed a lack of movement of V1 cytoplasmic foci, in contrast to rapid movements of individual YFP‐EML4‐ALK V3 WT foci (Fig 1E and F). While live‐cell imaging confirmed the presence of distinct cytoplasmic foci in HEK293 cells transfected with YFP‐EML4‐ALK V1 and V3 WT (Movie EV1 and EV3), these were not detected with the KD construct of each variant (Movie EV2 and EV4). As observed in fixed cells, EML4‐ALK V1 KD cells showed a dispersed localisation whereas the V3 KD protein gave a robust microtubule association (Movie EV2 and EV4). Further analysis suggested that YFP‐EML4‐ALK V3 WT foci had the ability to split and coalesce frequently and to diffuse in a random manner in the cytoplasm with speeds in the range of 0.5–3 μm/s that inversely correlated with foci size (Fig EV1E–H). Furthermore, kymographs revealed that WT V3 foci moved erratically at a mean velocity of 0.7 μm/s, while WT V1 cytoplasmic foci moved smoothly at a mean velocity of 0.3 μm/s (Fig 1G and H). Similar to WT V1, kinase‐dead (KD) V3 drifted smoothly at a mean velocity of 0.2 μm/s (Fig 1G and H). Taken together, these data indicate that EML4‐ALK V3 cytoplasmic foci are motile and able to merge and split, in contrast to static V1 cytoplasmic foci that behave as solid granules.

### Cytoplasmic EML4‐ALK V1 and V3 foci act as signalling hubs

We next investigated whether the cytoplasmic EML4‐ALK V1 and V3 foci are enriched in cytoplasmic components that are required for oncogenic, downstream signalling pathways. Since receptor tyrosine kinase (RTK) signalling is controlled by lipid‐ordered plasma membrane microdomains (Delos Santos *et al*, 2015), we examined whether EML4‐ALK V1 and V3 cytoplasmic foci are associated with lipid membranes by carrying out subcellular fractionation of H3122 and H2228 in the presence of detergent. Unlike the control protein, calnexin, which is an internal membrane protein, neither EML4‐ALK V1 nor V3 were solubilised in the presence of 1% Triton X‐100 treatment, but rather behaved similarly to DCP1B, a cytoplasmic ribonucleoprotein granule from the P‐body (Appendix Fig S1A–D). These data suggest that EML4‐ALK V1 and V3 foci are not membrane enclosed structures. In addition, we investigated whether EML4‐ALK V1 and V3 cytoplasmic foci are formed by multivalent interactions of protein–RNA complexes by carrying out subcellular fractionation of H3122 and H2228 cell lysates with RNase A. EML4‐ALK V1 and V3 fractionation was unaltered by RNase A treatment while, DCP1B, which is a ribonucleoprotein, was partially shifted to the supernatant fraction (Appendix Fig S1E–H). Since the behaviour of EML4‐ALK V1 and V3 upon subcellular fractionation was independent of lipid or RNA, it is likely that the formation of cytoplasmic foci of EML4‐ALK V1 and V3 is driven primarily by protein–protein interactions.

**Figure 2:**
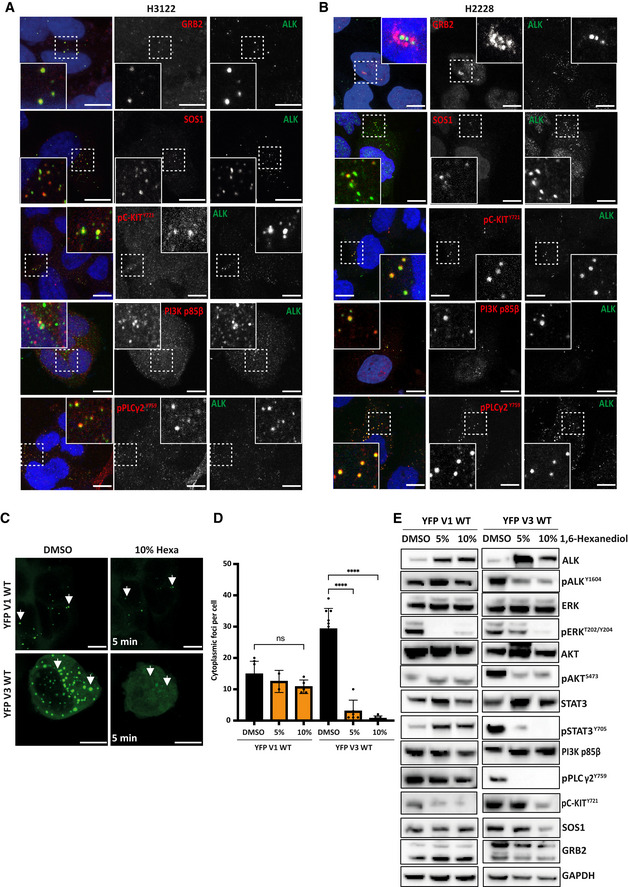
Cytoplasmic EML4‐ALK V1 and V3 foci contain downstream signalling proteins A, BH3122 and H2228 cells were stained for anti‐ALK (green), anti‐GRB2 (red), anti‐SOS1 (red), anti‐pC‐KIT^Y721^ (red), anti‐PI3K p85β or anti‐pPLCγ2^Y759^ and DAPI (blue). Scale bars, 10 μm; magnified views of a selected area are shown.CHEK293 cells transfected with YFP‐EM4L‐ALK V1 WT or V3 WT for 48 h and treated with 5% or 10% 1,6‐hexanediol. Representative still images after 5 min (taken from time‐lapse movies) are shown of cells treated with 10% 1,6‐hexanediol or DMSO. Scale bars, 10 μm; the arrowheads indicate cytoplasmic foci.DBox plot showing the number of cytoplasmic foci in DMSO and 5% or 10% Hexanediol (5 min). Data represent 5–10 counts from three independent experiments (*n = *3). Error bar represents SD of three biological replicates. ****P < 0.0001 in comparison with YFP‐EML4‐ALK V3 WT DMSO by one‐way ANOVA.ERepresentative Western blots of HEK293 transfected EML4‐ALK V1 WT and V3 WT for 48 h. Cells were treated with either 5% or 10% 1,6‐hexanediol for 5 min. DMSO was used as a control. Lysates were analysed for the phosphorylation and expression of the indicated antibodies. GAPDH was used as a loading control. Data representative of *n = *2 experiments.

**Figure EV2:**
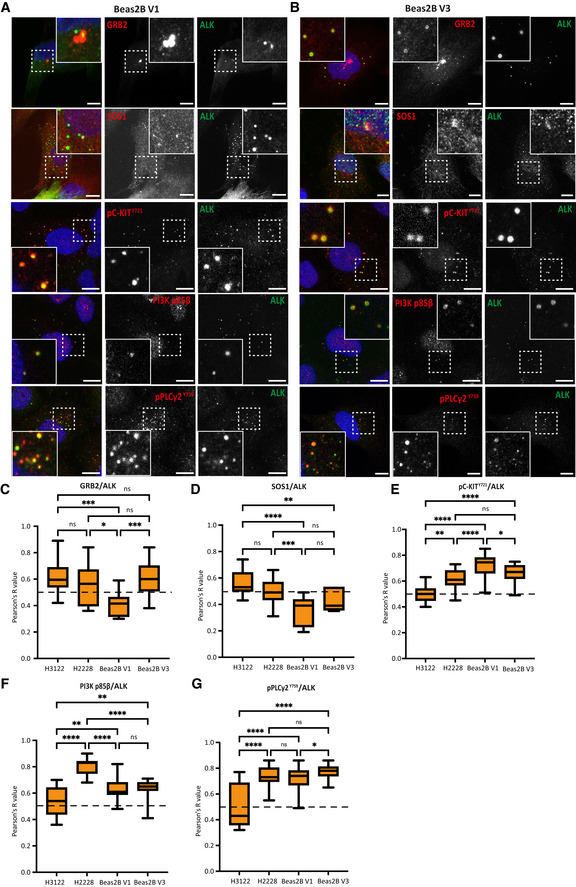
Association of signalling protein with active EML4‐ALK V1 and V3 cytoplasmic foci in inducible Beas2B cells A, BInducible Beas2B V1 and V3 cells were stained with anti‐ALK (green), anti‐GRB2 (red), anti‐SOS1 (red), anti‐pC‐KIT^Y721^ (red), anti‐PI3K p85β or anti‐pPLCγ2^Y759^ and DAPI (blue). Scale bars, 10 μm; magnified views of a selected area are shown.C–GIntensity profiles showing colocalisation between endogenous GRB2, SOS1, pC‐KIT^Y721^, PI3K p85β or pPLCγ2^Y759^ and ALK staining in different cell lines. *R* (Pearson’s correlation coefficient) measures the correlation between the indicated proteins and ALK signals. Pearson’s measurements are from 20 foci of 10–20 cells for each antibody combination. Data in all whisker plots represent counts from at least 10 cells, *n = *3. The central dashed band indicates the minimum Pearson *R* value (0.5) required for colocalisation. All whisker boxplots indicate the minimum and maximum Pearson *R* values of each cell line. *P < 0.5, **P < 0.01, ***P < 0.001, ****P < 0.0001 in comparison of each cell line by one‐way ANOVA.

As proteomic analyses and cellular assays have identified several cellular networks through which EML4‐ALK can signal (Hrustanovic *et al*, 2015; Zhang *et al*, 2016), we chose to look for components of the RAS/MAPK, PI3K/AKT and stem cell factor (SCF)/KIT signalling pathways that colocalised within those EML4‐ALK cytoplasmic foci. Using immunofluorescence microscopy, we observed the colocalisation of MAPK pathway adaptor proteins, GRB2 and SOS1, with EML4‐ALK V1 and V3 cytoplasmic foci in the patient‐derived H3122 and H2228 cells, and in the inducible Beas2B cells (Figs 2A and B, and EV2A–D). Along with the GRB2 and SOS1 proteins, EML4‐ALK V1 and V3 foci were locally enriched with other kinases, notably c‐KIT (phosphorylated on Tyrosine 721), the p85 beta regulatory subunit of PI3K, and PLCγ2 (phosphorylated on Tyrosine 759) in both endogenous H3122 (V1) and H2228 (V3) cells (Figs 2A and B, and EV2E–G), as well as in Beas2B V1 and V3 cells (Fig EV2A and B, E–G). Together, these data demonstrate recruitment of adaptor proteins and activated enzymes at the EML4‐ALK V1 and V3 cytoplasmic foci.

**Figure EV3:**
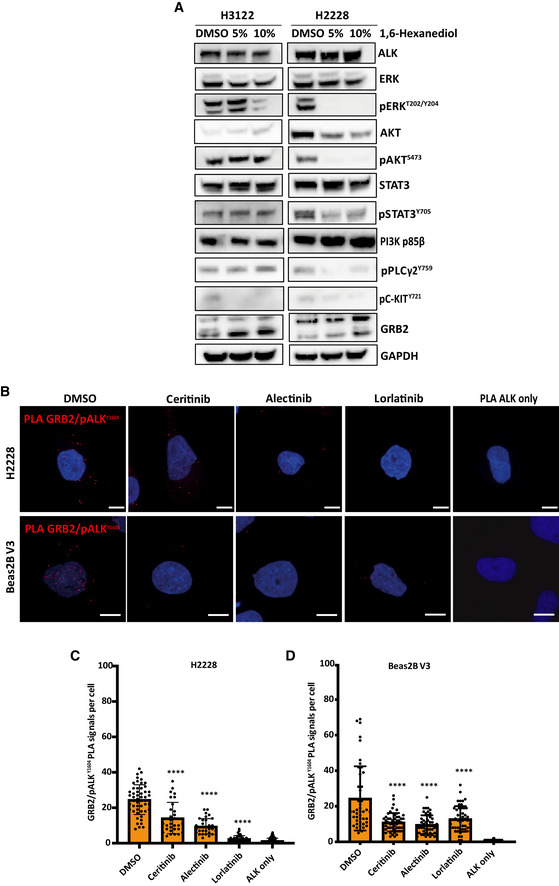
Loss of signalling proteins upon 1,6‐hexanediol and ALK inhibitors AH3122 and H2228 cells were treated with either 5% or 10% 1,6‐hexanediol for 5 min. DMSO was used a control. Lysates were analysed for the phosphorylation and expression of the indicated antibodies. GAPDH was used as a loading control. Data represent of *n = *2 experiments.BH2228 and Beas2B V3 cells were treated with ALK inhibitors for 4 h before fixation and staining with GRB2 and ALK antibodies for proximity ligation assay (PLA). Nuclei are indicated by DAPI staining (blue). Red foci indicate GRB2/ALK protein complexes. Single ALK antibody staining was used as a control for PLA interactions. Scale bars, 10 μm.C, DBar graphs representing the number of PLA foci per cell from B, GRB2/ pALK^Y1604^ PLA signals in H2228 cells and Beas2B V3. Data represent counts from at least 30 cells, *n = *3. Error bar represents SD of three biological replicates. ****P < 0.0001 in comparison with DMSO (empty vector) by one‐way ANOVA. Source data are available online for this figure.

We then investigated the nature of the EML4‐ALK V1 and V3 cytoplasmic foci using treatment with the aliphatic alcohol 1,6‐hexanediol which is able to disrupt the hydrophobic interactions that drive the formation of droplets by liquid–liquid phase separation (LLPS), but which does not affect solid protein aggregation or assemblies (Kroschwald *et al*, 2017). Treatment with 5% or 10% 1,6‐hexanediol dissolved most of the foci formed by EML4‐ALK V3 but not those formed by EML4‐ALK V1 (Fig 2C and D). Collectively, these data suggest that EML4‐ALK V3 cytoplasmic foci behave as a form of LLPS, while EML4‐ALK V1 forms static, solid foci. To determine the importance of the cytoplasmic foci on EML4‐ALK downstream signalling, we tested the consequences of their dissolution by 1,6‐hexanediol. Interestingly, the disruption of cytoplasmic foci by 1,6‐hexanediol resulted in significant loss of activated ALK (pY1640), ERK (pT202/Y204), AKT (pS473), STAT3 (pY705), PLCγ2 (pY759) and C‐KIT (pY721) in HEK293 cells expressing YFP‐EML4‐ALK V3 but had minimal impact in cells expressing YFP‐EML4‐ALK V1 (Fig 2E). The activation of downstream signalling was also significantly reduced by 1,6‐hexanediol treatment in patient‐derived H2228 (V3) cells, but not in H3122 (V1) cells (Fig EV3A).

We next used a proximity ligation assay (PLA) to visualise pair‐wise interactions between EML4‐ALK V3 and GRB2 in the cytoplasm of Beas2B cells and H2228 and observed that the occurrence of these foci was significantly reduced in the presence of ALK inhibitors, ceritinib, alectinib and lorlatinib, as would be expected for a signalling complex mediated by SH2 domain binding to a tyrosine phosphorylated protein (Fig EV3B–D). These data confirm recruitment of the MAPK pathway adaptor protein, GRB2, to the cytoplasmic regions of EML4‐ALK V3 foci and its loss upon ALK inhibition supporting the model that these foci potentially act as signalling hubs in the cytoplasm.

**Figure EV4:**
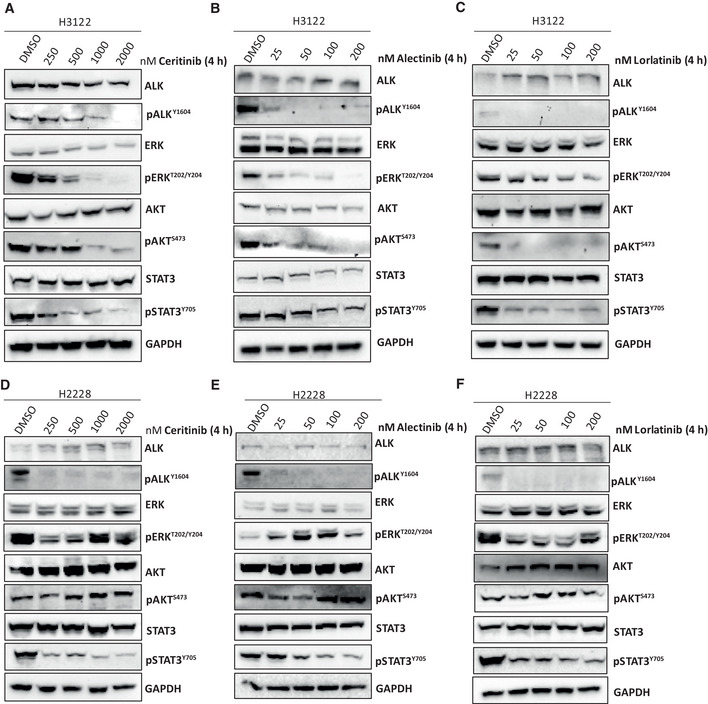
Effects of ALK inhibition on signalling pathways in H3122 and H2228 cells A–CRepresentative Western blots of serum‐starved H3122 cells treated with increasing doses of (A) ceritinib, (B) alectinib or (C) lorlatinib for 4 h. An equal volume of DMSO was used as control. Lysates were analysed by Western blotting and immunostaining for the proteins indicated and for their phosphorylation at the sites indicated. GAPDH was used as a loading control. Data represent of *n = *2 experiments.D–FSerum‐starved H2228 cells were treated with increasing doses of (D) ceritinib, (E) alectinib or (F) lorlatinib for 4 h. Lysates were analysed by Western blotting and immunostaining with the indicated antibodies. GAPDH was used as a loading control. Data represent of *n = *2 experiments. Source data are available online for this figure.

To assess the impact of EML4‐ALK V1 and V3 foci on these RAS/MAPK, PI3K/AKT and JAK/STAT pathways, we treated H3122 (V1) and H2228 (V3) cells with increasing doses of ALK inhibitors, ceritinib, alectinib and lorlatinib. In serum‐starved H3122 cells, EML4‐ALK V1 inhibition by ceritinib, alectinib or lorlatinib resulted in a dose‐dependent loss of activated ALK (pY1604), ERK (pT202/Y204), AKT (pS473) and STAT3 (pY705) (Fig EV4A–C). Inhibition of EML4‐ALK V3 in endogenous serum‐starved H2228 cells using ceritinib and lorlatinib resulted in significant loss of ALK (pY1604), STAT3 (pY705) and ERK (pT202/Y204) phosphorylation but had minimal impact on AKT (pS473) (Fig EV4D and F). Notably, inhibition of EML4‐ALK V3 with alectinib showed an increase of AKT (pS473) signal at most doses even when ALK (pY1604) activity was diminished (Fig EV4E). This suggests that H2228 (V3) cells are able to sustain RAS/MAPK and AKT/PI3K signalling pathways in the absence of ALK activity.

### Differential effects of ALK inhibitors on EML4‐ALK localisation

**Figure 3:**
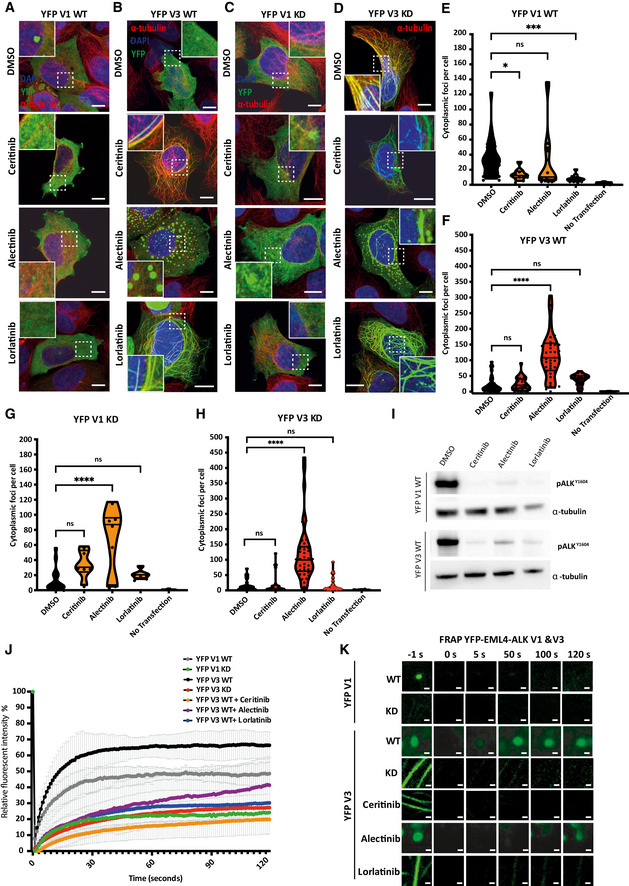
Different ALK inhibitors dissolve or maintain cytoplasmic EML4‐ALK V1 and V3 foci and redirect V3 to microtubules A–DHEK293 cells were transfected with EML4‐ALK V1 WT or V3 WT or kinase dead (KD) for 48 h. Cells were either untreated (DMSO) or treated with ALK inhibitors for 4 h before fixation and staining with anti‐GFP (green), anti‐α‐tubulin (red) and DAPI (blue). Scale bars, 10 μm; magnified views of a selected area are shown.E, FViolin plots show the number of cytoplasmic foci per cell from (A) and (B), respectively. Data represent counts from > 30 cells, *n* = 4. *P < 0.05, ***P < 0.001, ****P < 0.0001 in comparison with DMSO by one‐way ANOVA.G, HViolin plots show the number of cytoplasmic foci per cell from (C) and (D), respectively. Data represent counts from > 30 cells, *n* = 4. ****P < 0.001 in comparison with DMSO by one‐way ANOVA.IHEK293 cells were transfected with YFP‐EML4‐ALK V1 WT or V3 WT for 48 h and treated for 4 h with ALK inhibitors, ceritinib (500 nM), alectinib (100 nM) and lorlatinib (100 nM) or an equivalent volume of DMSO. Western blots of pALK^Y1604^ and α‐tubulin were used to assess relative abundance in transfected HEK293 cells.JAfter photobleaching, the fluorescence intensity of an area of a HEK293 cell transfected with YFP‐EML4‐ALK V1 WT, V3 WT in the presence or absence of ALK inhibitors, or YFP‐EML4‐ALK V1 or V3 KD, was plotted as a function of time. Each curve is the average of data of 40–50 individual cells. Error bar represents SD of three biological replicates.KStill images from FRAP analysis of YFP‐EML4‐ALK V1, V3 WT, KD or ALK inhibitors. Magnified views of a selected area are shown. Time is shown in seconds.

Targeting the EML4‐ALK fusion in NSCLC patients with the TKIs, crizotinib, ceritinib and alectinib, has been broadly successful. However, cancers harbouring EML4‐ALK V3 tend to respond less well to ALK inhibitors compared with cancers driven by EML4‐ALK V1 (Kwak *et al*, 2010; Shaw *et al*, 2011; Solomon *et al*, 2014; Woo *et al*, 2017; Christopoulos *et al*, 2018). We therefore investigated the behaviour of EML4‐ALK V1 and V3 cytoplasmic foci in the presence of ALK inhibitors. Consistent with the observation that catalytically inactive EML4‐ALK V1 and V3 did not readily form cytoplasmic foci, incubation of HEK293 cells expressing WT EML4‐ALK V1 and V3 with the ALK inhibitors, ceritinib and lorlatinib, led to loss of cytoplasmic foci with the V3 protein redirected to microtubules (Fig 3A and B, E and F). Meanwhile, ceritinib and lorlatinib treatment led to further enrichment of the V3 KD (kinase‐dead) protein on microtubules (Fig 3D and H), with V1 KD remaining widely dispersed in the cytoplasm (Fig 3C and G).

**Figure EV5:**
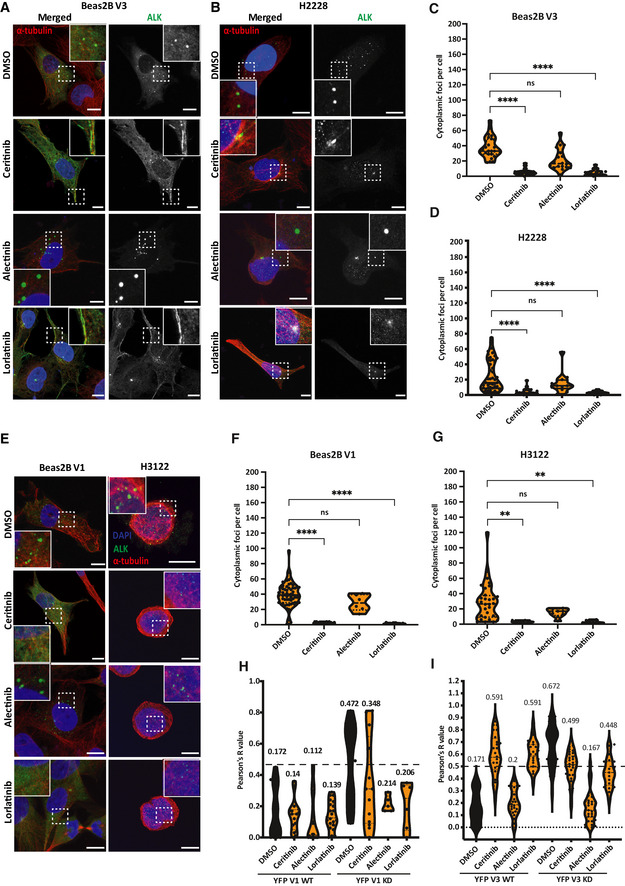
Effects of ALK inhibitors on localisation of EML4‐ALK V1 and V3 harbouring cell lines ABeas2B V3 cells were induced with doxycycline for 72 h and treated with ALK inhibitors or DMSO for 4 h. Cells were fixed and stained with anti‐ALK (green), anti‐α‐tubulin (red), and DAPI (blue). Scale bars, 10 μm; magnified views of a selected area are shown.BH2228 cells treated with ALK inhibitors or DMSO for 4 h. Cells were fixed and stained with anti‐ALK (green), anti‐α‐tubulin (red) and DAPI (blue). Scale bars, 10 μm; magnified views of a selected area are shown.C, DViolin plots representing the number of cytoplasmic foci per cell from (A) and (B), respectively. Data represent measurements taken from at least 20 cells, *n = *3. ****P < 0.0001 in comparison with DMSO in Beas2B V3 and H2228 by one‐way ANOVA.EH3122 cells and Beas2B V1 cells were treated with ALK inhibitors or DMSO for 4 h. Cells were fixed and stained with anti‐ALK (green) or anti‐GFP (green), anti‐α‐tubulin (red), and DAPI (blue). Scale bars, 10 μm; magnified views of a selected area are shown.F, GViolin plots representing the number of cytoplasmic foci per cell from (E). Data represent measurements taken from at least 10 cells, *n = *3. **P < 0.01, ****P < 0.0001 in comparison with DMSO by one‐way ANOVA.H, IIntensity profiles showing colocalisation between YFP‐EML4‐ALK V1 or V3 WT or KD and microtubules −/+ ALK inhibitors. *R* (Pearson’s correlation coefficient) measures the correlation between YFP and α‐tubulin signals. Pearson’s measurements from 20–30 cells for each construct.

Unexpectedly, incubation with alectinib enhanced the formation of EML4‐ALK V1 and V3 foci in transfected HEK293 cells (Fig 3A and B, E and F) and promoted the formation of cytoplasmic foci with the V1 KD and V3 KD proteins (Fig 3C and D, G and H). This distinct behaviour of EML4‐ALK V1 and V3 proteins in the presence of different ALK inhibitors was confirmed in both the Beas2B V3 and patient‐derived H3122 and H2228 cells (Fig EV5A–I). Consistent with these findings, live‐cell imaging of HEK293 cells treated with ceritinib or lorlatinib revealed strong microtubule localisation of YFP‐EML4‐ALK V3, whereas alectinib‐treated cells showed a re‐organisation of the YFP‐EML4‐ALK V3 into cytoplasmic foci (Movie EV5, EV6 and EV7). Colocalisation analysis confirmed that neither ALK inhibitor treatment (ceritinib, lorlatinib or alectinib) nor use of the kinase‐dead ALK mutant promoted microtubule localisation of EML4‐ALK V1 (Fig EV5H). In contrast, quantification of the colocalisation of YFP‐EML4‐ALK V3 with microtubules confirmed that ceritinib and lorlatinib treatment promoted microtubule localisation of the WT and kinase‐dead V3 protein (Fig EV5I). However, the colocalisation of EML4‐ALK V3 with microtubules was low in alectinib‐treated cells and similar to the WT V3 protein (Fig EV5I). In control experiments, A YFP‐tagged version of the ALK kinase domain alone (YFP‐ALK 1058‐1620) exhibited neither microtubule binding nor droplet formation and no effect on localisation was observed upon inhibition with ALK inhibitors (Appendix Fig S2A–C). Hence, while ceritinib and lorlatinib disfavoured the partitioning of EML4‐ALK V3 and V1 into cytoplasmic foci, the effect of alectinib was to promote the formation of these structures. As immunoblotting with phospho‐ALK antibodies (pY1604) indicated that all three inhibitors blocked the kinase activity of EML4‐ALK V1 and V3 (Fig 3I, Appendix Fig S2D and E), this difference in behaviour suggested that ALK catalytic activity *per se* does not drive formation of cytoplasmic foci.

To examine the dynamics of EML4‐ALK V1 and V3 proteins within cytoplasmic foci, and their dependence on ALK activity, we used a fluorescence recovery after photobleaching (FRAP) method. The active YFP‐EML4‐ALK V1 protein (WT) in foci recovered up to 40% after bleaching, suggesting that these foci are composed of only partially dynamic structures and consistent with more solid condensates (Fig 3J and K). However, the cytoplasmic foci formed by active YFP‐EML4‐ALK V3 protein (WT, in the absence of inhibitors) recovered rapidly to 70% after bleaching, suggesting that these are more dynamic structures, more characteristic of LLPS droplets (Fig 3J and K) (Movie EV8). Inactive YFP‐EML4‐ALK V3 (WT protein in the presence of ALK inhibitors, ceritinib and lorlatinib, or the mutant protein with the D1270N inactivating mutation) showed much slower recovery on microtubules than active YFP‐EML4‐ALK V3 (Fig 3J and K). In cells treated with alectinib, YFP‐EML4‐ALK V3 foci exhibited a faster FRAP recovery than the inactive proteins localised to microtubules, but much slower than the foci formed by active and uninhibited fusion protein (Fig 3J and K) (Movie EV9). Hence, these data confirm that the EML4‐ALK V1 foci are not as dynamic as V3 and behave more as a solid‐like condensate compared with the liquid‐like behaviour of V3 foci.

### Molecular basis of differences between variants and inhibitors in foci formation

**Figure 4:**
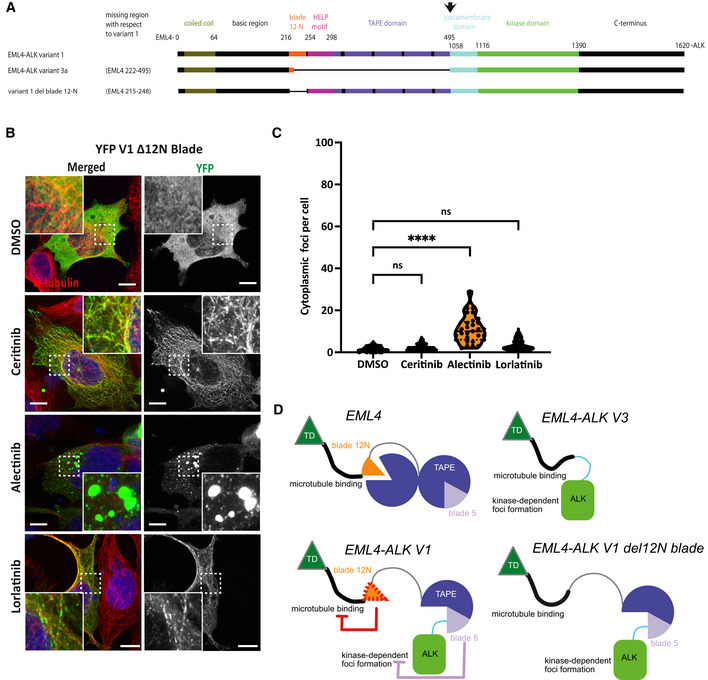
The deletion of 12N blade of EML4‐ALK V1 is redirected to microtubules upon ALK inhibition Linear representation of the structure of EML4‐ALK V1 and V3 variants and the deletion 12N blade mutant including residue numbering indicating domain boundaries.HEK293 cells were transfected with EML4‐ALK V1 deletion 12N blade for 48 h. Cells were either untreated (DMSO) or treated with ALK inhibitors for 4 h before fixation and staining with anti‐GFP (green), anti‐α‐tubulin (red) and DAPI (blue). Scale bars, 10 μm; magnified views of a selected area are shown.Violin plot shows the number of cytoplasmic foci per cell from (B). Data represent counts from > 30 cells, *n* = 3. ****P < 0.0001 in comparison with DMSO by one‐way ANOVA.Models summarising the domain structures of EML4 and EML4‐ALK proteins consisting of a coiled‐coil region (dark green) followed by basic region (black), 12N blade (orange), blades 1–4 (purple) and blade 5 (lilac). The ALK JM domain is coloured light blue, and the kinase domain is coloured light green.

To begin to explore the molecular basis for differences in the localisation of EML4‐ALK V1 and V3, we generated a set of mutants lacking regions or domains that are present in V1 but not V3 (Fig 4A, Appendix Fig S3A) and examined their subcellular localisation and signalling upon loss of ALK activity. The TAPE domain of EML4 comprises a set of 14 individual blade sub‐domains that form a tandem pair of 7‐bladed beta‐propellers. Of those mutants generated, only the deletion of the 12N blade enabled V1 to localise to microtubules in ceritinib and lorlatinib‐treated cells (Fig 4B and C). In addition, alectinib‐treated cells expressing the Δ12N blade mutant showed robust formation of cytoplasmic foci (Fig 4B and C). Most of the other deletion mutants showed a cytoplasmic diffusion of EML4‐ALK V1 in the presence of any of the three ALK inhibitors (Appendix Fig S3B–D and G). However, a V1 deletion mutant that was lacking the partial blade 5 exhibited high numbers of cytoplasmic foci in untreated cells, and loss of ALK activity let to an increase of those cytoplasmic foci (Appendix Fig S3E and F). We conclude that these specific regions within the EML4 portion of the EML4‐ALK V1 fusion protein contribute to the macroscopic localisation of the protein: the 12N blade region prevents microtubule binding of the kinase‐inactive protein, and the blade 5 region inhibits foci formation. This is intriguing because these two blades lie at the N‐ and C‐terminal ends, respectively, of the portion of the TAPE domain present in the EML4‐ALK V1 fusion, closest to the domains whose interaction they affect (Fig 4D).

**Figure 5:**
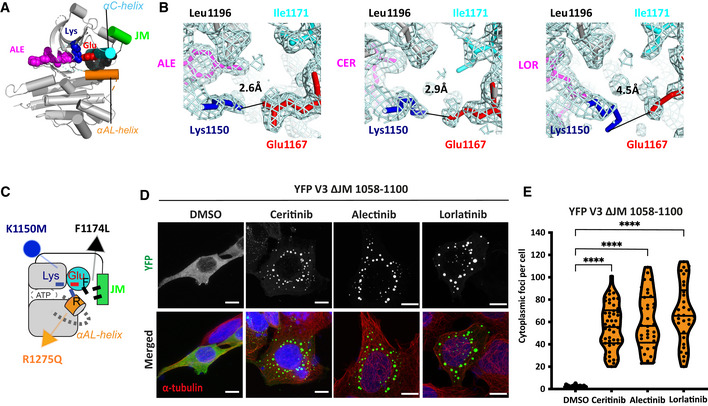
Structure and dynamics of the ALK kinase domain in the presence of TKIs View of ALK (grey) bound to alectinib (magenta) centred on the active site highlighting the αC‐helix (cyan), αAL‐helix (orange) and Lys‐Glu salt bridge (blue‐red), based on the crystal structure (PDB:3AOX).Magnified views of ALK, in the vicinity of the Lys‐Glu salt bridge, bound to inhibitors: alectinib (ALE, PDB:3AOX), ceritinib (CER, PDB:4MKC) and lorlatinib (LOR, PDB:4CLI). The cyan wiremesh is a 2Fo‐Fc electron density map. The black line marks the interatomic distance between terminal atoms in the Lys‐Glu salt bridge.Schematic illustration of the regulatory features of the ALK kinase domain and the effects of mutations. The side chains of key residues are shown as sticks. The αC‐helix of inactive ALK interacts with the JM domain through hydrophobic contacts and the αAL‐helix through polar contacts. The interactions between αC‐helix, the JM domain and the A‐loop are destabilised by mutations such as F1174L, R1275Q that drive the activation of ALK. The Lys‐Glu salt bridge can be disrupted by an inactivating mutation (K1150M).HEK293 cells were transfected with EML4‐ALK V3 ΔJM 1,058–1,100 for 48 h. Cells were either untreated (DMSO) or treated with ALK inhibitors for 4 h before fixation and staining with anti‐GFP (green), anti‐α‐tubulin (red)and DAPI (blue). Scale bars, 10 μm; magnified views of a selected area are shown.Violin plot representing the number of cytoplasmic foci counted per cell from (D). Data represent counts from at least 30 cells, *n = *3. ****P < 0.0001 in comparison with DMSO by one‐way ANOVA.

To gain further insights into the molecular basis of the different effects of ALK inhibitors, we compared the crystal structures of the unphosphorylated ALK kinase domain in complex with ceritinib (PDB code 4MKC) lorlatinib (PDB code 4CLI) and alectinib (PDB code 3AOX) (Sakamoto *et al*, 2011; Friboulet *et al*, 2014; Johnson *et al*, 2014). All three structures show the same overall conformation of ALK, as exemplified by the structure in which alectinib is bound to the ATP site (Fig 5A). Notably, the unphosphorylated activation loop (A‐loop) is partially ordered and adopts a helical conformation that would not be present in the phosphorylated, active kinase (αAL‐helix, Fig 5A). However, close examination of the structures and the electron density maps on which they are based revealed differences in the salt bridge between K1150 on the β3 strand and G1167 on the αC‐helix, which is a hallmark of the kinase active state. While the presence of this salt bridge is clear in the alectinib‐bound ALK structure because the modelled positions of K1150 and G1167 are supported by the electron density map, the positions of these residues are not well‐defined in the ceritinib‐bound and lorlatinib‐bound ALK structures because the electron density maps are more ambiguous (Fig 5B). These differences may reflect the distinct shapes of these inhibitors and their interactions with ALK. Alectinib is a flat molecule that penetrates into the kinase structure and interacts with hydrophobic residues that contact the αC‐helix, such as L1196. In contrast, both lorlatinib and ceritinib, and indeed most other ALK TKIs, are non‐planar, penetrate less deeply into the kinase structure and interact less extensively with hydrophobic residues that contact the αC‐helix. We therefore propose that the ability of alectinib to induce formation of EML4‐ALK V1 and V3 foci arises from its stabilisation of the αC‐helix and the Lys‐Glu salt bridge in positions that are characteristic of an active state.

### Foci formation is dependent on an active kinase conformation

While the Lys‐Glu salt bridge is critical for kinase activity, other interactions of the C‐helix are autoinhibitory (Fig 5C). One such interaction is with the juxtamembrane (JM) domain, mediated through a cluster of hydrophobic residues (Phe‐core). We first determined the localisation of a mutant in which the JM domain was deleted ΔJM (Δ1058–1100) in the presence of ALK inhibitors. The EML4‐ALK ΔJM protein was largely diffused in the cytoplasm in untreated cells but formed foci (mean of 55–65 droplets per cell) that did not colocalise with microtubules in the presence of all three ALK inhibitors (Fig 5D–E). Hence, we conclude that release of the kinase from an autoinhibited state of the kinase via movement of the JM segment is implicated in the formation of EML4‐ALK V3 cytoplasmic foci.

**Figure 6:**
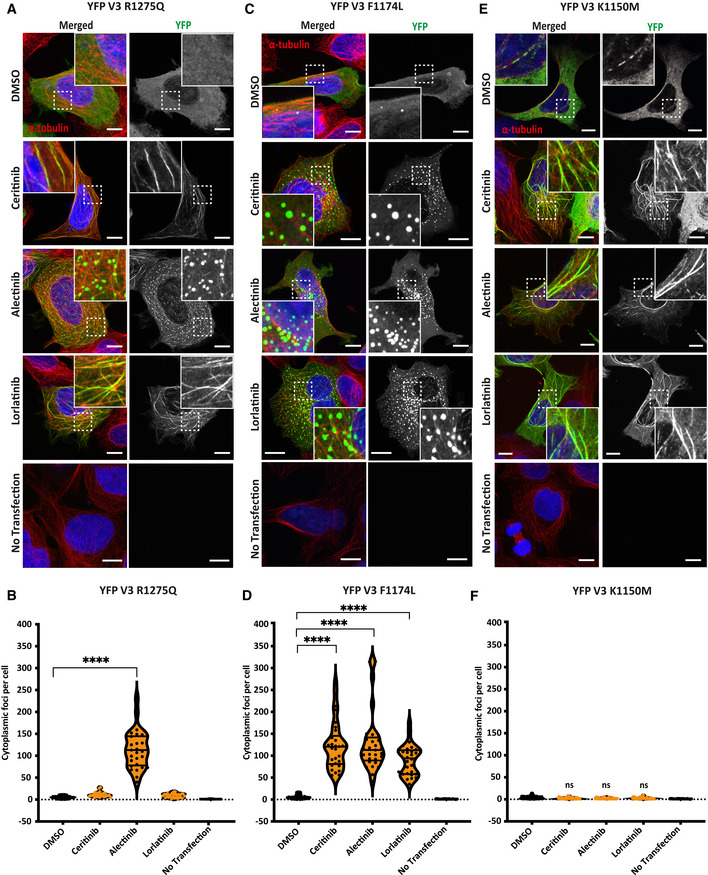
Point mutations in ALK kinase domain alter EML4‐ALK V3 localisation A–FHEK293 cells were transfected with (A) YFP‐EML4‐ALK V3 K1150M, (C) R1275Q or (E) F1174L constructs and treated with ALK inhibitors or DMSO for 4 h before fixation and staining with anti‐GFP (green), anti‐α‐tubulin (red) and DAPI (blue). Scale bars, 10 μm; magnified views of a selected area are shown. (B), (D), (F). Violin plots representing the number of cytoplasmic foci counted per cell from (A), (C) and (E). Data represent counts from at least 30 cells, *n = *3 or 4. ****P < 0.0001 in comparison with DMSO by one‐way ANOVA.

We next investigated the role of autoinhibitory interactions of the αC‐helix with the JM domain and A‐loop in foci formation. These two autoinhibitory interactions are disrupted by activating mutations (e.g. F1174L, R1275Q) in ALK in neuroblastoma that drive kinase activity (Bossi *et al*, 2010; Lee *et al*, 2010; Bresler *et al*, 2014). Introducing activating mutations such as these into EML4‐ALK might be expected to promote foci formation, whereas disruption of the salt bridge should block foci formation (Fig 5C). To test this hypothesis, we generated these mutations in EML4‐ALK V3 and examined their behaviour. The EML4‐ALK V3 R1275Q mutant behaved similarly to WT, binding to microtubules in the presence of ceritinib and lorlatinib, and forming foci (mean of 115 per cell) with alectinib (Fig 6A and B). Like the ΔJM mutant, the other activating mutant, F1174L, exhibited a dispersed cytoplasmic localisation in the absence of ALK inhibitor but formed cytoplasmic foci (> 90) in the presence of ceritinib, alectinib and lorlatinib (Fig 6C and D). Immunoblotting confirmed that all three inhibitors abolished kinase activity of the V3 R1275Q mutant (Appendix Fig S4A and B), whereas the V3 F1174L mutant was less sensitive to all three inhibitors (Appendix Fig S4A and C). We conclude that interaction of the αC‐helix with the JM domain inhibits foci formation, but that interaction of the αC‐helix with the A‐loop is not critical in promoting or inhibiting foci formation.

We also generated an EML4‐ALK V3 K1150M mutant incapable of forming the salt bridge between K1150 and G1167. This mutant exhibited strong microtubule binding and produced very few droplets in the presence of all three ALK inhibitors, including alectinib (Fig 6E and F; Movie EV10). Immunoblotting confirmed the loss of active ALK (pY1604) in untreated and ALK‐treated EML4‐ALK V3 K1150M transfected HEK293 cells (Appendix Fig S4D). The inability of alectinib to rescue the formation of liquid droplets in the EML4‐ALK V3 K1150M salt bridge mutant contrasted with the ability of alectinib to promote foci formation in another kinase‐dead mutant D1270N. We conclude that the Lys‐Glu salt bridge has a key role in formation of EML4‐ALK V3 foci and that these structures are dependent on an active conformation of the kinase, not kinase activity perse.

### Dimerisation drives cytoplasmic foci formation

**Figure 7:**
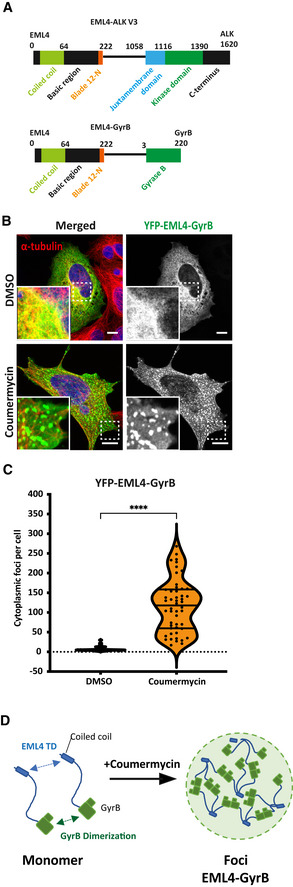
Stoichiometric mismatch between EML4 and ALK proteins is required for foci formation Linear representation of the structure of EML4‐ALK V3 and EML4‐GyrB fusion proteins including residue numbering indicating domain boundaries.HEK293 cells transfected with YFP‐EML4‐GyrB and either untreated (DMSO) or treated with Coumermycin A1 to induce dimerisation of GyrB before fixation and staining with anti‐GFP (green), anti‐α‐tubulin (red) and DAPI (blue). Scale bars, 10 μm; magnified views of a selected area are shown.Violin plot showing the number of cytoplasmic foci per HEK293 cell transfected with YFP‐EML4‐GyrB. Data represent counts from > 30 cells, *n = *4. ****P < 0.0001 in comparison with DMSO by unpaired *t*‐test.Schematic model showing the mechanism of EML4‐GyrB foci formation. EML4‐GyrB foci are formed by dynamic cross‐linking interactions between the EML4 trimerisation domain (TD) and dimerisation of GyrB domain in the presence of coumermycin A1.

Following on from our observation that foci formation depends on an active kinase conformation, and because the activation of RTKs generally proceeds through dimerisation of the kinase domain (Lemmon & Schlessinger, 2010), we hypothesised that direct dimerisation of active ALK kinase domains in the context of trimeric EML4‐ALK V3 may drive cytoplasmic foci formation. If this were the case, then replacing the ALK kinase domain in the EML4‐ALK fusion with an alternative dimerisation domain ought to generate foci. To test this concept, we took advantage of using coumermycin A1 to induce dimerisation of GyrB‐fused proteins, which has been used to study the dimerisation and activation of signalling receptors, such as RAF or JAK2 (Farrar *et al*, 1996; Mohi *et al*, 1998). A YFP‐tagged construct was made in which residues 1–222 of EML4, which is the portion of EML4 present in V3, was fused to GyrB rather than to the ALK kinase domain (Fig 7A). In untreated HEK293 cells, this protein bound to microtubules with excess material diffusely distributed in the cytoplasm, but in the presence of coumermycin A1 the YFP‐EML4‐GyrB fusion protein was observed to condense into cytoplasmic foci, at an average of 117 foci per cell (Fig 7B and C). Hence, dimerisation of GyrB in the context of the trimeric EML4 N‐terminal region is sufficient to drive foci formation (Fig 7D). This is consistent with a model in which the formation of EML4‐ALK cytoplasmic foci is dependent on the dimerisation of the ALK kinase domain.

## Discussion

**Figure 8:**
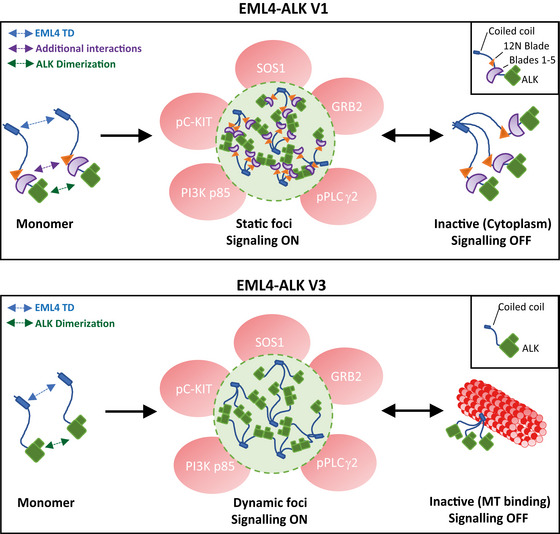
Models summarising the mechanism of EML4‐ALK V1 and V3 foci formation Schematic model showing the mechanism of EML4‐ALK V1 and V3 cytoplasmic foci formation. EML4‐ALK V1 foci are formed by dynamic cross‐linking interactions between the EML4 trimerisation domain (TD), dimerisation of ALK domains and additional interactions via the 12N Blade/Blades 1–5 domains. The shorter EML4‐ALK V3 variant forms foci via interactions of EML4 TD and ALK kinase dimerisation. In both variants, EML4‐ALK trimers arises from the stoichiometric mismatch between the trimeric EML4 N‐terminus and the dimers formed by ALK in its active conformation which leads to the formation of foci in the cytoplasm that exclude large complexes such as microtubules. The EML4‐ALK foci act as signalling platforms by recruiting several proteins and allowing signal transduction. When the ALK kinase domain is monomeric cross‐linking does not occur and the inactive EML4‐ALK V3 trimers instead localise to microtubules through the EML4 TD and basic region, whereas inactive EML4‐ALK V1 trimers are diffusely localised in the cytoplasm.

In a mechanism common among oncogenic RTK fusions, self‐association of the non‐kinase component—in this case the EML4 TD at the N‐terminus—of EML4‐ALK mimics the oligomerisation‐dependent activation mechanism of RTKs and leads to constitutive activation of the ALK kinase domains (Schlessinger, 2000; Lemmon & Schlessinger, 2010). We extend this concept by proposing a model in which the dimerisation of active ALK kinase domain in the context of the EML4‐ALK fusion drives cytoplasmic foci formation. The stoichiometric mismatch between the trimeric EML4 portion of the fusion protein and the dimeric ALK kinase may contribute to the formation of higher‐order structures. Thus, EML4‐ALK will tend to cross‐link through transient kinase domain interactions when the ALK moiety is in an active conformation, leading to phase separation (Fig 8). It is likely that additional interactions via the partial TAPE domain in EML4‐ALK V1 produce more solid‐like, static foci, whereas EML4‐ALK V3 molecules lack these interactions and form a dynamic molecular network. EML4‐ALK cytoplasmic foci act as a platform in which several adaptor proteins (GRB2, SOS1) and signalling kinases (PLCγ2, PI3K p85, c‐KIT) are recruited to facilitate cellular signalling (Fig 8). ALK inhibitors block kinase activation, disrupt signalling and, with the exception of alectinib, dissolve these foci. Soluble, kinase‐inactive V3 trimers then relocate to microtubules, through the EML4 basic region, but the additional interactions in V1 generates higher‐order aggregates that are excluded from microtubules (Fig 8). The foci are themselves not associated with microtubules, which implies that either the additional interactions between EML4‐ALK trimers interfere with microtubule binding or that bulky structures such as microtubules are excluded from the cytoplasmic foci.

The structural basis of ALK kinase domain‐kinase domain interactions in the context of either the native ALK receptor or the EML4‐ALK fusion is unknown. However, crystal structures of ALK reveal the basis for autoinhibition and how it is reversed by activating mutations. In inactive ALK, the JM segment forms a β‐turn motif that folds over the kinase domain and interacts with the αC‐helix and A‐loop via a hydrophobic, phenylalanine (Phe) core that comprises residues such as F1245 and F1174. Mutations of these residues in cancer are strongly activating because they release the autoinhibitory interactions within the Phe core, enabling autophosphorylation to occur (Bresler *et al*, 2014). A region of the unphosphorylated activation loop (A‐loop) of ALK between F1271 and R1279 folds into an α‐helix that is incompatible with activating autophosphorylation of Y1278 (Bossi *et al*, 2010; Lee *et al*, 2010). Hydrogen bond interactions between the αC‐helix and the A‐loop helix stabilise the inactive conformation of ALK. Disruption of these interactions through mutations such as R1275Q promotes activation by unwinding the A‐loop helix, which releases the side chain of Y1278 to be autophosphorylated (Epstein *et al*, 2012). The release of the A‐loop and autophosphorylation are not essential for EML4‐ALK foci formation because unphosphorylated EML4‐ALK V3 can still form foci in the presence of alectinib, and, furthermore, the R1275Q mutation does not promote foci formation. Instead, the data point to an active position of the C‐helix as being the critical feature of an ALK conformation that supports EML4‐ALK foci formation. Based on the striking differences in the localisation of EML4‐ALK V3 upon treatment with different ALK inhibitors, we propose that alectinib mimics kinase activation by stabilising the Lys‐Glu salt bridge and an active conformation, promoting dimerisation and phase separation whereas ceritinib and lorlatinib simply inhibit the kinase and can only support foci formation in the context of an activating mutation such as F1174L. The structure of ALK in an active, dimeric, conformation is required to provide further insights into the activation mechanism and foci formation.

Analysis of the EML4‐ALK V1 and V3 cytoplasmic foci reveals that they contain adaptor and signalling proteins, such as GRB2 and SOS1, that function in the RAS/MAPK pathway and others including c‐KIT, PI3K p85β and PLCγ2 that function in PI3K/AKT, SCF/KIT and phosphoinositide signalling. We therefore suggest that both EML4‐ALK V1 and V3 transduce downstream signalling within and from these cytoplasmic foci. Indeed, some of these findings are consistent with those recently published by Tulpule *et al* (2021), who have suggested a similar mode of signalling by which fusions of RTKs, including ALK and RET, direct RAS/MAPK axis signals from membraneless cytoplasmic protein granules. It is notable that more studies now report the existence of cytoplasmic foci as a mechanism of activation in the cell; for example, the autoactivation of PLK4 by phase separation acts as a mechanism for centriole biogenesis (Park *et al*, 2019). Chemical inhibition of ALK kinase led to loss of GRB2 and EML4‐ALK V3 interaction as observed by PLA assay. While we observed a loss of RAS/MAPK, Jak/STAT3 and AKT/PI3K signalling in EML4‐ALK V1 H3122 cells upon ALK inhibition, consistent with previous reports (Hrustanovic *et al*, 2015; Zhang *et al*, 2016), we observed no significant disruption of AKT/PI3K and RAS/MAPK signalling pathways upon ALK inhibition by alectinib in the H2228 cell line that harbours EML4‐ALK V3. Increased foci numbers upon short‐term alectinib treatment may thus sustain or increase signalling output in the short‐term window of our experiments. However, alectinib is clearly effective at blocking signalling over the long‐term treatment because the efficiency of alectinib in ALK+ cancer patients is impressive with a survival of almost 2 years (Camidge *et al*, 2019). Several kinases are able to phosphorylate AKT at Ser473 including PDK‐1, integrin‐linked kinase (ILK) and AKT itself (Memmott & Dennis, 2009) and AKT activation is stimulated by other upstream pathways (e.g. cytokine receptors, G protein‐coupled receptors (GPCRs), integrins, RTKs and B‐ and T‐cell receptors (Nitulescu *et al*, 2018)). Hence, it is likely that the inhibition of ALK may be readily bypassed in the V3 expressing H2228 cell line in order to maintain anti‐apoptotic AKT/PI3K signalling (Duronio, 2008; Zhao *et al*, 2015). Signalling in the EML4‐ALK V3 cells is robust in the presence of any of the three inhibitors tested and so appears independent of the ability of these compounds to stabilise or destabilise the foci. In contrast to the effects of ALK inhibitors, the disruption of EML4‐ALK V3 cytoplasmic foci by 1,6‐hexanediol led to loss of downstream signalling but had a minimal effect on EML4‐ALK V1 signalling. Although we note that 1,6‐hexanediol has recently been shown to disrupt cellular phosphorylation (Düster *et al*, 2021), the minimal effect of the 1,6‐hexanediol on V1 signalling suggests this is not a major factor with respect to EML4‐ALK. New, more specific molecular tools and further studies will enable us to understand the relationship between the structure and dynamics of EML4‐ALK foci, ALK activity and downstream signalling.

EML4‐ALK V3 in NSCLC is associated with a relatively poor response to ALK inhibitors and more aggressive metastatic disease than longer EML4‐ALK variants such as V1 (Woo *et al*, 2017; Christopoulos *et al*, 2018, 2019). A better understanding of the molecular biology of EML4‐ALK V3 and its response to ALK inhibitors may help to improve the outcomes for these higher‐risk NSCLC patients. Here, we have demonstrated the ability of EML4‐ALK V1 and V3 to form *de novo* foci within the cytoplasm that have different characteristics and observed striking differences in the localisation of EML4‐ALK V1 and V3 upon treatment with different ALK inhibitors. It remains to be seen if these differences at a cellular level will translate to differences on patient response or outcomes, and our findings should not affect the selection of which ALK inhibitor is used for the treatment of patients. The liquid nature of the bodies formed by EML4‐ALK V3 is evidenced by their rapid internal rearrangement and their behaviour in the cytoplasm, moving randomly and frequently dividing and coalescing, and stands in contrast to the static nature of the foci formed by EML4‐ALK V1. Unlike EML4‐ALK V3 liquid‐like foci, the EML4‐ALK V1 foci were not dissolved by 1,6‐hexanediol treatment, and they also exhibited more limited internal rearrangement as measured by FRAP analysis, suggesting that they are more solid in nature. This is consistent with the key structural difference between EML4‐ALK V1 and V3—the longer variant includes the incomplete globular TAPE domain of EML4 that is predicted to aggregate due to exposure of its hydrophobic core and which confers dependence on the chaperone Hsp90 for stability (Richards *et al*, 2014). As the existence of LLPS foci, such as FUS, which are formed by phase separation, is implicated in neurodegenerative diseases, the presence of similar compartments in cancers, such as EML4‐ALK^+^ NSCLC, could also contribute to the progression and aggressiveness of the disease (Qin *et al,* 2021). Therefore, we suggest that disruption of the foci using specific compounds which alter phase behaviour or inhibitors of key protein–protein interactions might provide an additional route to targeting EML4‐ALK V3, as part of a concerted strategy to improve the outcomes of these higher‐risk patients.

## Materials and Methods

### Plasmid construction and mutagenesis

EML4‐ALK V3 and ALK 1058–1620 were cloned into a version of pcDNA3.1‐hygro (Invitrogen) as previously described (Richards *et al*, 2014), providing an N‐terminal YFP tag for transfection of HEK293 cells. D1270N, K1150M, R1275Q and F1174L mutants were generated by QuickChange procedure (Agilent Technologies) and confirmed by sequencing. To generate the ΔJM construct, the region encoding residues 1,058–1,100 of ALK was deleted from pcDNA3.1‐hygro YFP‐EML4‐ALK‐V3 by PCR and the plasmid re‐ligated in frame. To generate the EML4‐ALK V1 deletion constructs, the regions encoding residues of each domain of EML4 was deleted from pcDNA3.1‐hygro YFP‐EML4‐ALK‐V3 by PCR and the plasmid re‐ligated in frame. All constructs were confirmed by sequencing. YFP‐EML4‐GyrB fusion was constructed by replacing the *ALK* portion of EML4‐ALK V3 with a sequence encoding residues 3–220 of GyrB (the ATPase domain of *E. coli* DNA gyrase subunit B).

### Cell culture, transfection and drug treatments

HEK293 (Human embryonic kidney cells) and NCI‐H2228 (a Human NSCLC cell line harbouring EML4‐ALK variant 3b) cells were obtained from ATCC. All cells were obtained within the last 5 years, stored in liquid nitrogen, and maintained in culture at 37°C in a 5% CO_2_ atmosphere for a maximum of 2 months. We relied on the provenance of the original collection for authenticity. Cell lines were regularly tested for mycoplasma contamination using a highly sensitive and specific PCR‐based assay (EZ‐PCR Mycoplasma kit, Geneflow). NCI‐H3122, NCI‐H2228 and Beas2B were cultured in RPMI‐1640 medium, and HEK293 in DMEM. All media were from Invitrogen‐GIBCO and supplemented with 10% heat‐inactivated foetal bovine serum (FBS), 100 IU/ml penicillin and 100 mg/ml streptomycin. Beas2B cell line was treated with doxycycline for 72 h to induce expression of EML4‐ALK V1 and V3. HEK293 were transiently transfected using Fugene HD (Promega, U.K.) according to manufacturer’s instructions. Ceritinib (LDK378), alectinib (CH5424802) and lorlatinib (PF‐06463922) were purchased from Selleck Chemicals, and stock solutions were prepared in DMSO. Unless otherwise indicated, the following compounds were added to cells for 4 h: ceritinib (0.5 µmoles/l); alectinib (0.1 µmoles/l); lorlatinib (0.1 µmoles/l); the compounds were diluted in fresh media before each experiment, and control cells were treated with the same volume of DMSO (Table 1).

**Table 1:** Compounds used and concentrations.

Compound	Supplier	Final concentration (nM)
Ceritinib (LDK378)	Selleck Chemicals	500
Alectinib (CH5424802)	Selleck Chemicals	100
Lorlatinib (PF‐06463922)	Selleck Chemicals	100

### Dissolving EML4‐ALK foci by 1,6‐hexanediol

HEK293 cells expressing YFP‐EML4‐ALK V1 or V3, and patient‐derived H3122 and H2228 cells were treated with 5% or 10% of 1,6‐hexanediol (Sigma) for 5 min. 1,6‐Hexanediol was freshly prepared in RPMI media. Live‐cell imaging of HEK293 YFP‐EML4‐ALK V1 or V3 cells was performed using RPMI media −/+ 1,6‐hexanediol. The same field or view was used to image cells before and after hexanediol treatment at specific time points. H3122 and H2228 cells were treated with 5% or 10% 1,6‐hexanediol for 5 min, then lysed in M‐PER lysis buffer containing protease and phosphatase inhibitor cocktails and analysed by Western blotting.

### Inducible dimerisation of EML4‐GyrB fusion

Forty‐eight hours following transfection with the YFP‐EML4‐GyrB construct, HEK293 cells were treated for 8 h with either 0.1 µmoles/l coumermycin A1 (Promega) or an equivalent volume of DMSO. The average number of cytoplasmic foci formed per cell was measured and analysed by Imaris (v.9.3) software. Data represent the mean of at least three independent experiments ± SD.

### Indirect immunofluorescence microscopy

Cells grown on acid‐etched glass coverslips were washed with PBS and fixed with 3.7% formaldehyde in PBS buffer for 10 min. Cells were kept in blocking buffer (3% BSA in PBS) for 1 h and incubated for 2 h or overnight with primary antibodies diluted in 3% BSA/PBS buffer followed by 1‐h incubation with secondary antibodies (Table 2). Primary antibodies were against, α‐tubulin mouse (1:1,000; Sigma), α‐tubulin rabbit (1:800; 18251; Abcam), GFP (1:1,000; 6556; Abcam), GFP (1:1,000; sc‐9996; Santa Cruz Biotechnology), ALK rabbit (D5F3) (1:100; CST), ALK (31F12) mouse (1:100; CST), GRB2 rabbit (1:1,000; PA1‐10033; Invitrogen), GRB2 (1:100) mouse (C7; Santa Cruz Biotechnology), SOS1 mouse (1:100; MCA2887; Bio‐Rad;), PLCγ2 (Y759) rabbit (1:100; AP0785; ABclonal), PI3K p85β mouse (1:100; 1B180967; Abcam), c‐KIT (Y721) (1:100; 44‐494G; Thermo Fisher Scientific). Secondary antibodies were Alexa Fluor‐488 and ‐594 goat anti‐rabbit and goat anti‐mouse IgGs (1:200; Invitrogen). Imaging was performed on a Zeiss LSM880 + Airyscan Inverted confocal microscope using a 40× oil objective (numerical aperture, 1.4). Z‐stacks comprising of 10–20 ×0.3 µm sections were acquired. Images were analysed using ImageJ (v.2.0.0).

**Table 2:** Antibodies used for immunofluorescence (IF) and Western blotting (WB) and dilutions.

Antibody	Supplier	Identifier	IF dilution	WB dilution
Anti‐α‐tubulin mouse monoclonal	SIGMA‐ALDRICH	T5168	1:1,000	
Anti‐α‐tubulin rabbit polyclonal	Abcam	ab15246	1:800	
Anti‐GFP rabbit polyclonal	Abcam	ab6556	1:1,000	1:1,000
Anti‐GFP mouse monoclonal	Santa Cruz Biotechnology	sc‐9996	1:1,000	1:1,000
Anti‐ALK (D5F3) rabbit monoclonal	CST	3633	1:100	1:1,000
Anti‐ALK (31F12) mouse monoclonal	CST	3921	1:100	1:1,000
Anti‐phospho‐ALK (Y1604) rabbit monoclonal	CST	3341	1:100	1:1,000
Anti‐GRB2 rabbit monoclonal	Invitrogen	PA1‐10033	1:200	1:1,000
Anti‐GRB2 (C7) mouse monoclonal	Santa Cruz Biotechnology	sc‐8034	1:100	1:500
Anti‐SOS1 mouse monoclonal	BIO‐RAD	MCA2887	1:100	1:1,000
Anti‐SOS1 rabbit monoclonal	CST	5890		1:1,000
Anti‐phospho PLCγ2 (Y759) rabbit monoclonal	ABclonal	AP0785	1:100	1:1,000
Anti‐ PI3K p85β rabbit monoclonal	Abcam	ab180967	1:100	1:1,000
Anti‐ phospho‐c‐KIT (Y721) rabbit monoclonal	Thermo Fisher Scientific	44‐494G	1:100	1:1,000
Anti‐STAT3 mouse monoclonal	CST	9139		1:1,000
Anti‐phospho STAT3 (Y705) rabbit monoclonal	CST	9145		1:1,000
Anti‐AKT (pan) (40D4) mouse monoclonal	CST	2920		1:1,000
Anti‐phospho AKT (S473) rabbit monoclonal	CST	4060		1:1,000
Anti‐ERK (L34F12) mouse monoclonal	CST	4696		1:1,000
Anti‐phospho ERK (T202/Y204) rabbit monoclonal	CST	4370		1:1,000
Anti‐calnexin C5C9 rabbit monoclonal	CST	2679		1:1,000
Anti‐DCP1B (D2P9W) rabbit monoclonal	CST	13233		1:1,000
Anti‐GAPDH rabbit monoclonal	Abcam	ab37168		1:2,000

### *In situ* proximity ligation assay (PLA)

Adherent cells were cultured in the appropriate media and treated with ALK inhibitors for 4 h before fixation. Cells were fixed with 3.7% (vol/vol) paraformaldehyde for 10 min and permeabilised with PBS containing 0.5% Triton X‐100 for a further 5 min. Cells were incubated with blocking buffer (3% BSA in PBS) for 1 h and then with the antibodies, as indicated, diluted in the same buffer for overnight. Duolink^®^ proximity ligation assays were carried out according to the manufacturer's instructions (Duolink^®^, Sigma). The average number of PLA dots detected per cell was calculated using DotCount software, and data represent the mean of at least two independent experiments ± SD.

### Live‐cell imaging

Time‐lapse imaging was performed using a Zeiss LSM880 + Airyscan Inverted confocal microscope using a 40× oil objective and a scan zoom of 4. Cells were cultured in glass‐bottomed culture dishes (Ibidi) and maintained at 37°C in an atmosphere supplemented with 5% CO_2_ using a ZL multi S1 Incubator box system. Drugs were added just before imaging with pre‐warmed OptiMEM (Thermo Fisher Scientific) containing 10% FBS. Z‐stacks comprising of twenty 1 μm steps were acquired every 1 s for 1 min. Stacks were processed into maximum intensity projections and movies prepared using ImageJ (v.2.0.0).

### Fluorescence Recovery After Photobleaching (FRAP) assay

Live‐cell FRAP measurements were made in HEK293 cells expressing fluorescently tagged YFP‐EML4‐ALK V1 and V3 WT or kinase‐dead (KD) protein. For FRAP, a region of interest (ROI) of 40 × 40 µm was bleached with 10 iterations at 100% argon 488 nm and 100% 405 nm laser power simultaneously. A second ROI of 40 × 40 µm was used as control (no photo‐bleach) to represent the background. One image was captured prior to bleaching and then images were collected every 1 s for 2 min following bleaching. The fluorescence intensity profiles of the ROI were determined using Zeiss software. The corrected fluorescence intensity was calculated by removing the background (no Bleach ROI) using the following equation and was expressed as percentage: (Intensity of ROI – Background Intensity)/Intensity of pre‐Bleach.

### Colocalisation measurements and tracking analysis

Cells stained with GFP/α‐tubulin, GRB2/ALK, SOS1/ALK, PI3Kp85β/ALK, pC‐KIT^Y721^/ALK or pPLCγ2^Y759^/ALK antibodies for immunofluorescence and 20–30 cells were analysed for colocalisation studies. The colocalisation analysis was measured using Colo2 in ImageJ (v.2.0.0) software. A 100 pixels × 100 pixels ROI was positioned over the interphase cell, and the Pearson *R* values were calculated. Data represent the Pearson *R* values of at least two independent experiments ± SD. For tracking analysis, 12‐s movies of HEK293 YFP‐EML4‐ALK V3 WT foci were analysed to calculate mean droplet speeds using TrackMate v5.2.0 in ImageJ (Plugins ‐> Tracking ‐> TrackMate) (Tinevez *et al*, 2017). Images were filtered to detect foci using “LoG detector” (Laplacian of Gaussian) with an estimated foci diameter of 1‐2 pixels and a threshold of 1. For kymograph analysis, time‐lapse series of YFP fluorescence from HEK293 cells expressing YFP‐EML4‐ALK V1 or V3 WT or YFP‐EML4‐ALK V1 or V3 KD were analysed using ImageJ software. Velocities of individual events were calculated using a custom macro on FiJi. All measurements were taken from at least two independent experiments.

### Subcellular fractionation

Cells from 10‐cm dishes were resuspended in 500 μl of buffer A (10 mM) Tris–HCl (pH 7.4), 1 mM EDTA, 250 mM sucrose, supplemented with 1× HALT protease inhibitors (Thermo Fisher Scientific) and lysed by passing through a 26‐G needle. For Triton X‐100 treatment, lysates were split evenly into two ultracentrifugation tubes and one tube was supplemented with Triton X‐100 to a final concentration of 1% prior to ultracentrifugation. For RNase A treatment, lysates were incubated with or without 1 µg/µl RNase A for 30 min at room temperature prior to ultracentrifugation. The samples were then spun at 100,000 *g* for 1 h at 4°C in an Optima MAX Ultracentrifuge (Beckman Coulter). Supernatant and pelleted fractions were separated, resuspended with Laemmli sample buffer, boiled at 95°C for 5 min and analysed by SDS–PAGE.

### Cell extracts and Western blotting analysis

For dose response experiments, cells were serum‐starved for 24 h prior to harvesting and were then lysed using M‐PER mammalian protein extraction reagent (Thermo Fisher Scientific) containing a Halt™ protease inhibitor cocktail (Thermo Fisher Scientific) and Halt™ phosphatase Inhibitor cocktail (Thermo Fisher Scientific). Immunoblot analyses were carried out with anti‐phospho‐ALK (Y1604) (1:1,000, 3341; CST), anti‐ALK (1:1,000, D5F3; CST), anti‐ERK (1:1,000, L34F12; CST), anti‐phospho‐ERK (T202/Y204) (1:1,000, D13.14.E3; CST), anti‐AKT (1:1,000, 40D4; CST), anti‐phospho‐AKT (S473) (1:1,000, D9E; CST), anti‐STAT3 (1:1,000, 124H6; CST), anti‐phospho‐STAT3 (Y705) (1:1,000, 3E2; CST), GRB2 rabbit (1:1,000; PA1‐10033; Invitrogen), SOS1 mouse (1:1,000; MCA2887; Bio‐Rad;), PLCγ2 rabbit (Y759) rabbit (1:1,000; AP0785; ABclonal), PI3K p85β (1:1,000; 1B180967; Abcam), c‐KIT (Y721) (1:1,000; 44‐494G; Thermo Fisher Scientific), anti‐calnexin (1:1,000, C5C9; CST), anti‐DCP1B (1:1,000, D2P9W; CST), GAPDH (1:2,000, ab37168; Abcam) and anti‐α‐tubulin (1:2,000, T5168; Sigma) antibodies. Secondary antibodies were rabbit or mouse horseradish peroxidase‐conjugated secondary antibodies (1:1,000; Amersham). The blots were visualised using the Pierce™ ECL Western blotting substrate (32106; Pierce).

### Statistical analysis

All quantitative data represent means and standard deviation (SD) of at least two independent experiments. Statistical analyses were performed using one‐way ANOVA from Prism 9.0 software. ****P < 0.0001, ***P < 0.001, **P < 0.01, *P < 0.05.

## Author contributions

JS, MWR and RB conceived and planned the experiments. JS carried out the experiments. MWR generated new reagents. JS and MWR wrote the manuscript with support from RB. JC and AMF helped supervise the project. RB supervised the project. All authors provided critical feedback and helped shape the research, analysis and manuscript.

## Conflict of interest

The authors declare that they have no conflict of interest.

## Supporting information



Appendix

Expanded View Figures PDF

Source Data for Expanded View and Appendix

Movie EV1

Movie EV2

Movie EV3

Movie EV4

Movie EV5

Movie EV6

Movie EV7

Movie EV8

Movie EV9

Movie EV10

Source Data for Figure 2

Source Data for Figure 3

## Data Availability

No primary datasets have been generated or deposited.
