# Supplementary material for: Phase‐separated foci of EML4‐ALK facilitate signalling and depend upon an active kinase conformation
Source: EMBO Rep. 2021 Oct 18;22(12):e53693. doi: 10.15252/embr.202153693 (PMC8647013; doi:10.15252/embr.202153693)
Supplement: Supplementary file 1 — Appendix [file EMBR-22-e53693-s005.pdf]

Table of Content for Appendix Figures

Phase-separated foci of EML4-ALK facilitate signalling and depend upon an active kinase conformation

Sampson et al.

Appendix Figure S1 and figure legend........2

Appendix Figure S2 and figure legend........3

Appendix Figure S3........5

Appendix Figure S3 legend........6

Appendix Figure S4 and figure legend........7

Appendix Figure S1

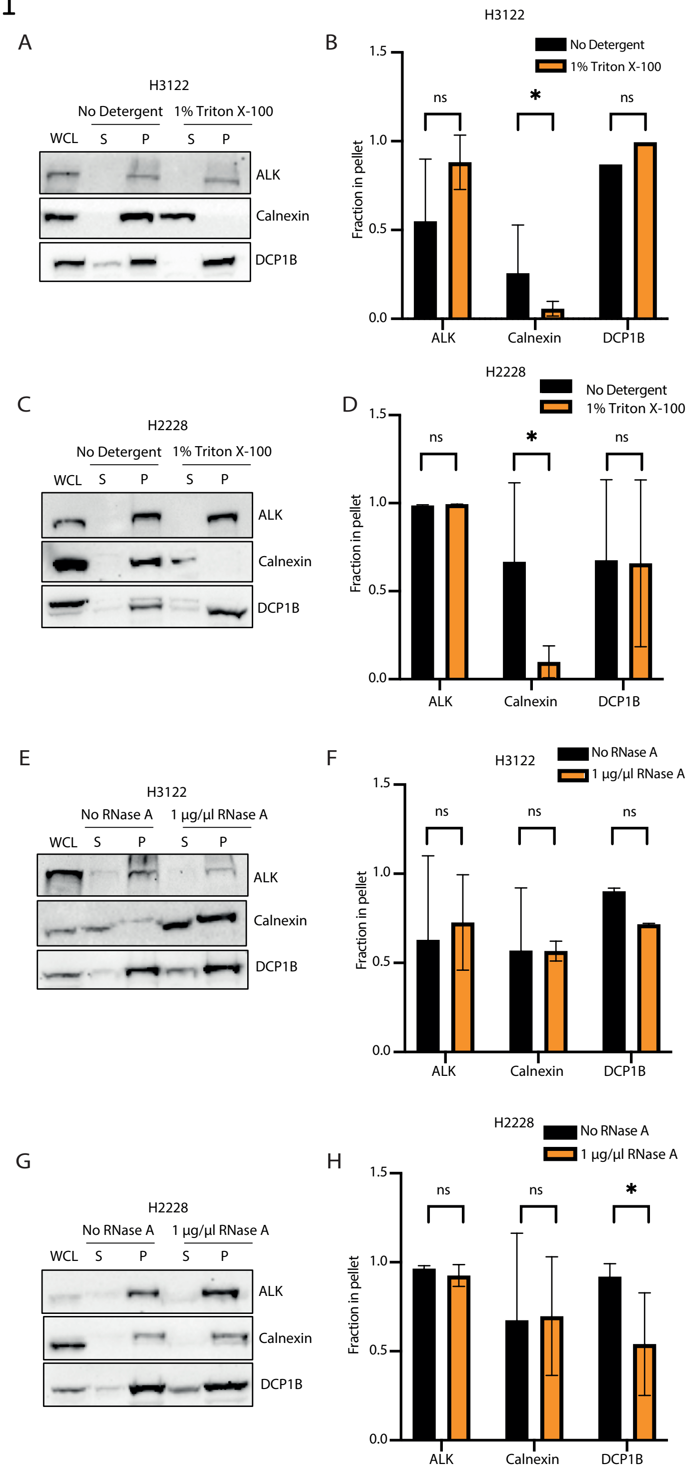

Appendix Figure S1. EML4-ALK V1 and V3 are membraneless cytoplasmic foci rich in protein-protein complexes

A, C. H3122 and H2228 cell lysates fractionated by ultracentrifugation +/- 1% Triton X-100 and analysed by western blotting with the indicated antibodies. **B, D.** Fraction in pellet was calculated as ratio of the insoluble fraction to total (supernatant plus insoluble fractions) as analysed by western blotting A and C. Data representative of three biological replicates (n=3). *P< 0.5 in comparison to no detergent by two-way ANOVA. **E, G.** H3122 and H2228 cell lysates fractionated by ultracentrifugation following +/- 1 µg/µL RNase A for 30 minutes

and analysed by western blotting with the indicated antibodies. **F, H.** Fraction in pellet was calculated as ratio of the insoluble fraction to total (supernatant plus insoluble fractions) as analysed by western blotting E and G. Data representative of three biological replicates (*n*=3). *P< 0.5 in comparison to no detergent by two-way ANOVA.

Appendix Figure S2

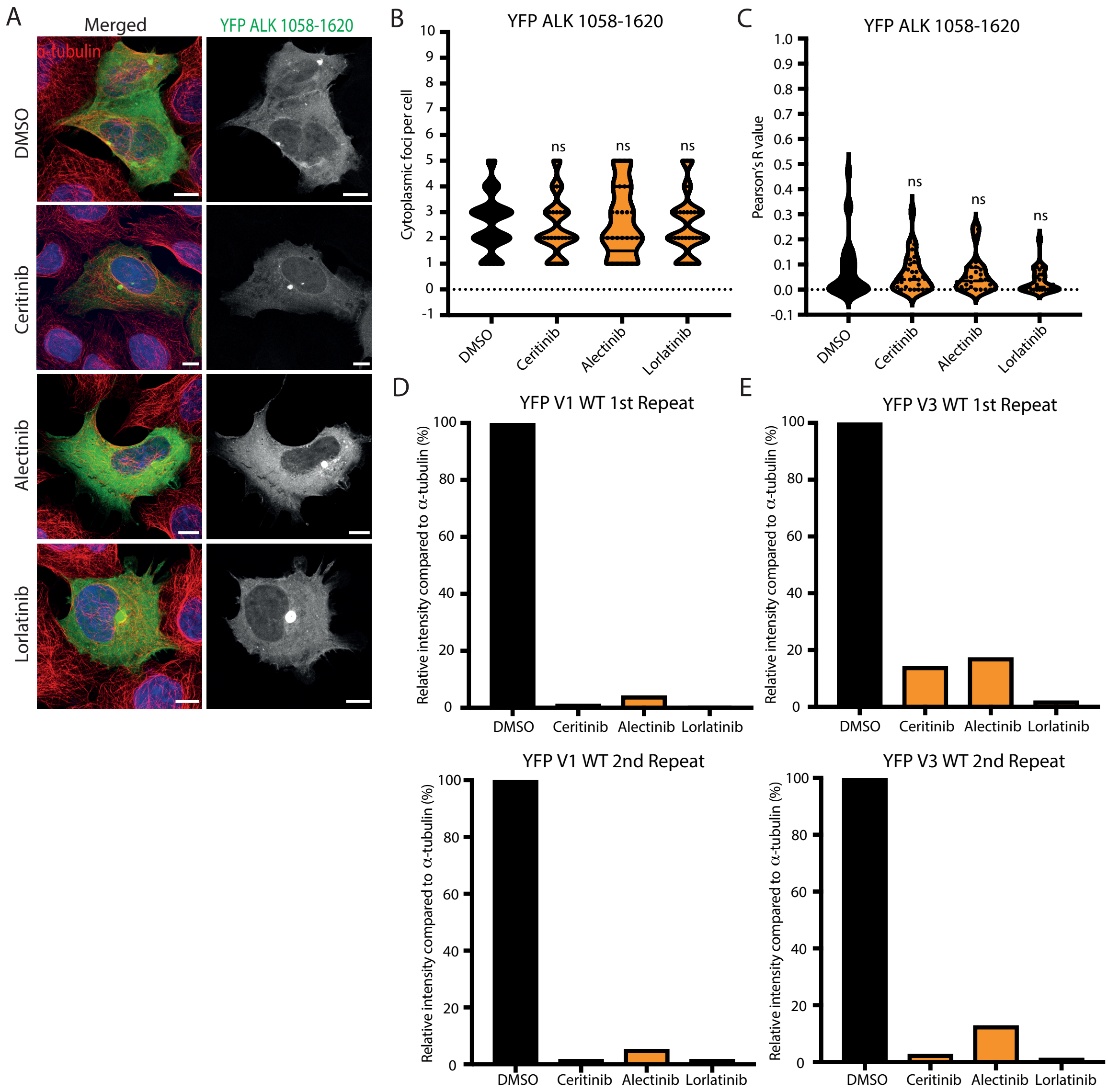

Appendix Figure S2. Effects of ALK inhibitors on localisation of YFP-ALK 1058-1620 and on the phosphorylation of EML4-ALK V1 and V3 WT at Y1604

A. HEK293 cells were transfected with YFP-ALK 1058-1620 and treated with ALK inhibitors or DMSO for 4 hours before fixation and staining with anti-GFP (green), anti-α-tubulin (red), and DAPI (blue). Scale bars, 10 μm; magnified views of a selected area are shown. **B**. Violin plot representing the number of droplets per cell from A.

Data represent measurements taken from at least 20 cells, *n*=2. **C**. Violin plot represents intensity profiles showing co-localisation between YFP-ALK 1058-1620 and microtubules in the presence of ALK inhibitors or DMSO. *R* (Pearson's correlation coefficient) measures the correlation between YFP and α-tubulin signals. Pearson's measurements from 30-50 cells for each treatment. **D, E**. HEK293 cells were transfected with YFP-EML4-ALK V1 WT or V3 WT for 48 hours and treated for 4 hours with ALK inhibitors, ceritinib (500 nM), alectinib (100 nM) and lorlatinib (100 nM) or an equivalent volume of DMSO. Western blots of $pALK^{Y1604}$ and α-tubulin (Fig. 3I) were used to assess relative abundance in transfected HEK293 cells. $pALK^{Y1604}$ band intensity was quantified relative to α-tubulin. Each repeat is shown in a separate graph (*n*=2).

Appendix Figure S3

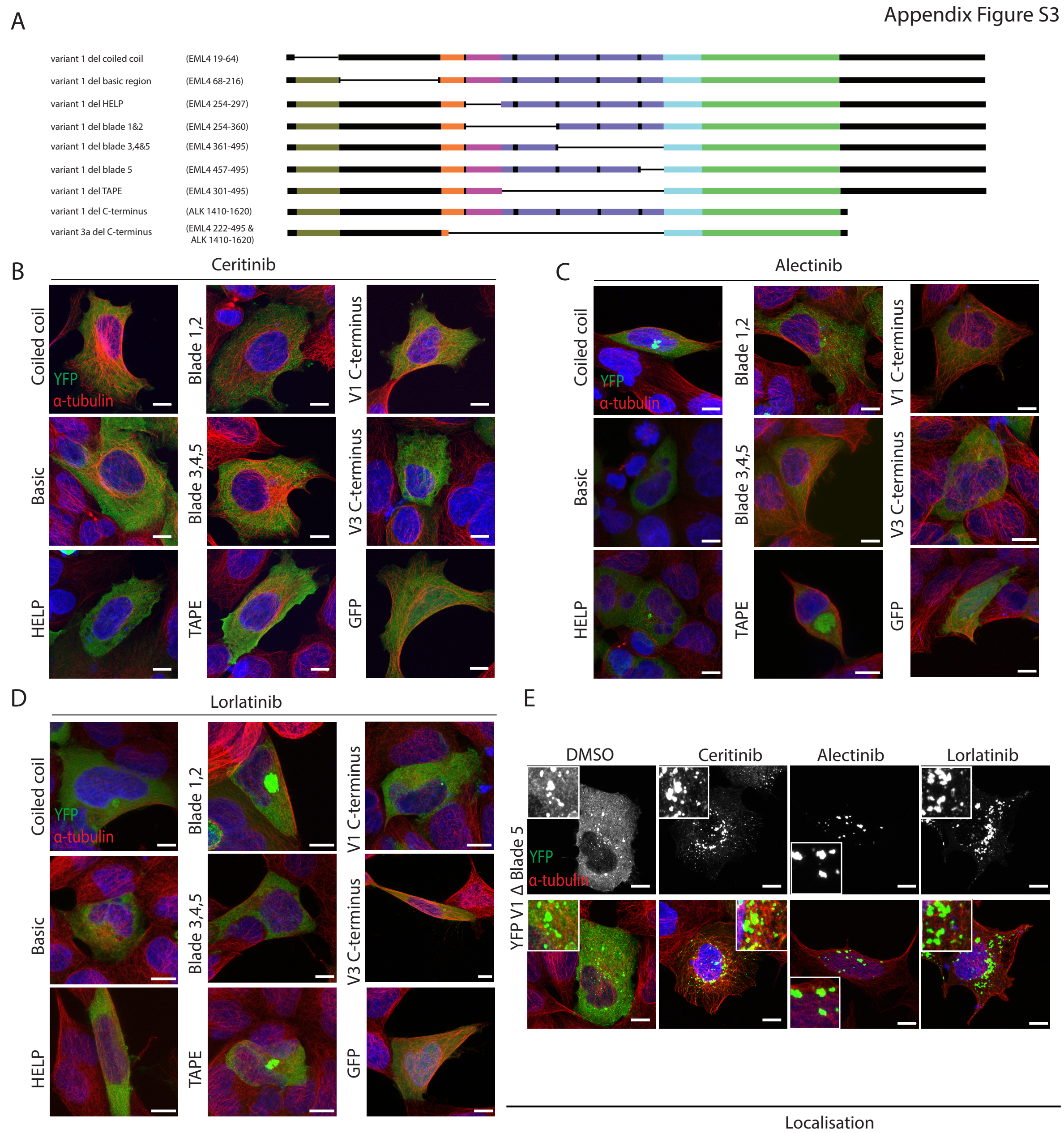

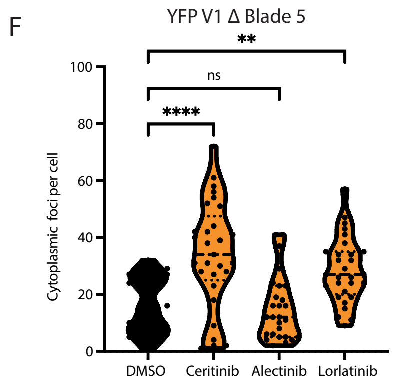

G

	Localisation			
Mutation	DMSO	Ceritinib	Alectinib	Lorlatinib
EML4-ALK V1	Cytoplasm/foci	Cytoplasm	Cytoplasm/foci	Cytoplasm
EML4-ALK V3a	Cytoplasm/foci	MT binding	Cytoplasm/foci	MT binding
V1 ΔCoiled coil	Cytoplasm	Cytoplasm	Cytoplasm	Cytoplasm
V1 ΔBasic	Cytoplasm	Cytoplasm	Cytoplasm	Cytoplasm
V1 Δ12N Blade	Cytoplasm	MT binding	Cytoplasm/foci	MT binding
V1 ΔHELP	Cytoplasm	Cytoplasm	Cytoplasm	Cytoplasm
V1 ΔBlade 1,2	Cytoplasm	Cytoplasm	Cytoplasm	Cytoplasm
V1 ΔBlade 3,4,5	Cytoplasm	Cytoplasm	Cytoplasm	Cytoplasm
V1 ΔBlade 5	Cytoplasm/foci	Cytoplasm/foci	Cytoplasm/foci	Cytoplasm/foci
V1 ΔTAPE	Cytoplasm	Cytoplasm	Cytoplasm	Cytoplasm
V1 ΔC-terminus	Cytoplasm	Cytoplasm	Cytoplasm	Cytoplasm
V3 ΔC-terminus	Cytoplasm	Cytoplasm	Cytoplasm	Cytoplasm

Appendix Figure S3. Effect of ALK inhibitors on EML4-ALK V1 deletion mutants

A. Linear representation of the structure of EML4-ALK V1 and V3 variants and the deletion mutants including residue numbering indicating domain boundaries. **B, C, D.** HEK293 cells were transfected with YFP-EML4-ALK V1 deletion mutants as indicated for 48 hours and treated for 4 hours with ALK inhibitors, (**B**) ceritinib (500 nM), (**C**) alectinib (100 nM) or (**D**) lorlatinib (100 nM) or an equivalent volume of DMSO. Cells were stained with anti-GFP (green), anti-α-tubulin (red), and DAPI (blue). Scale bars, 10 μm. **E.** HEK293 cells were transfected with EML4-ALK V1 deletion blade 5 for 48 hours. Cells were either untreated (DMSO) or treated with ALK inhibitors for 4 hours before fixation and staining with anti-GFP (green), anti-α-tubulin (red), and DAPI (blue). Scale bars, 10 μm; magnified views of a selected area are shown. **F.** Violin plot shows the number of cytoplasmic foci per cell from E. Data represent counts from >30 cells, $n = 3$. $**P < 0.01$, $****P < 0.0001$ in comparison to DMSO by one-way ANOVA. **G**. Table summarizing the phenotypes of EML4-ALK V1 deletion mutants in DMSO and ALK inhibitors.

Appendix Figure S4

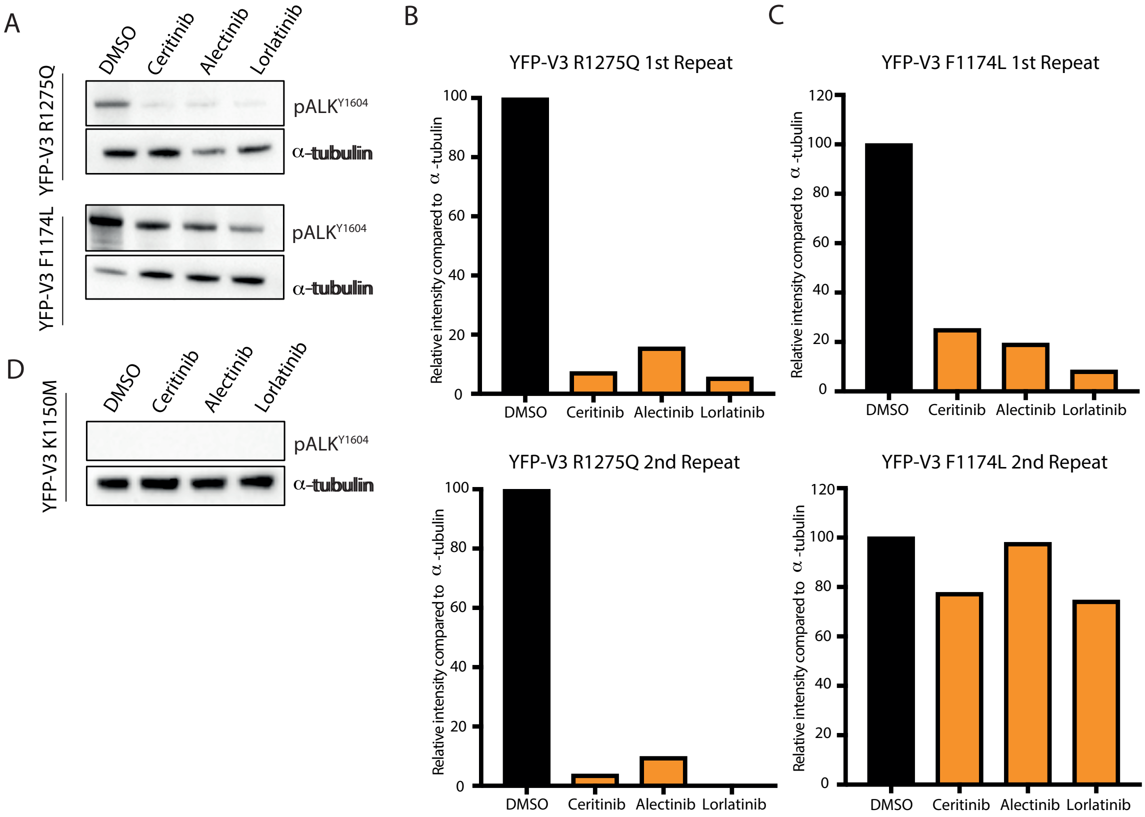

Appendix Figure S4. Loss of pALK^{Y1064} in EML4-ALK V3 mutants

A. HEK293 cells were transfected with YFP-EML4-ALK V3 R1275Q or F1174L, **D.** K1150M for 48 hours and treated for 4 hours with ALK inhibitors, ceritinib (500 nM), alectinib (100 nM) and lorlatinib (100 nM) or an equivalent volume of DMSO. Western blots of pALK^{Y1604} and α-tubulin were used to assess relative abundance in transfected HEK293 cells. **B, C.** pALK^{Y1604} band intensity was quantified relative to α-tubulin from A. Each repeat is shown in a separate graph (*n*=2).
