# Supplementary material for: Phase‐separated foci of EML4‐ALK facilitate signalling and depend upon an active kinase conformation
Source: EMBO Rep. 2021 Oct 18;22(12):e53693. doi: 10.15252/embr.202153693 (PMC8647013; doi:10.15252/embr.202153693)
Supplement: Supplementary file 2 — Expanded View Figures PDF [file EMBR-22-e53693-s008.pdf]

Expanded View Figures

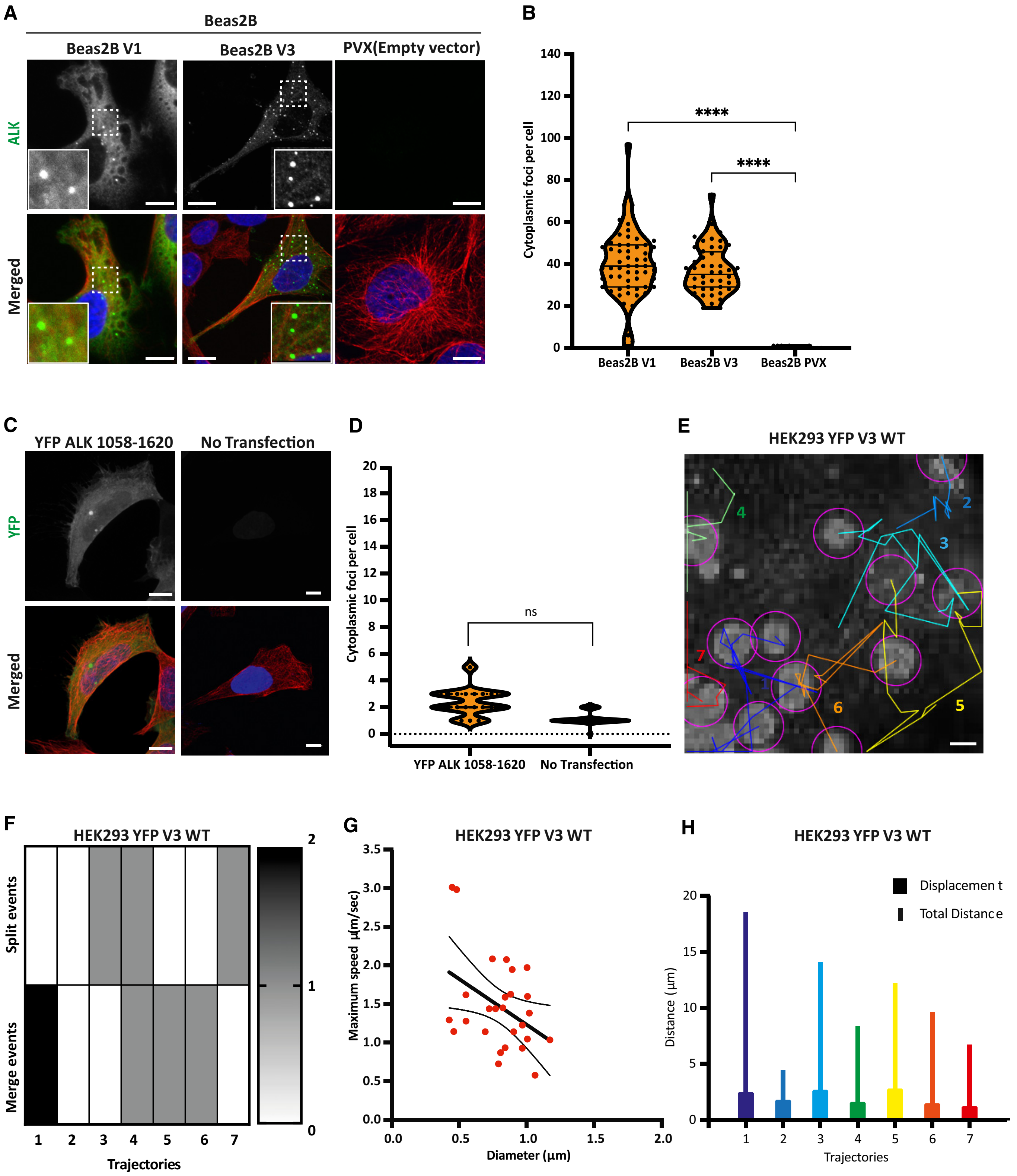

Figure EV1.

Figure EV1. Beas2B inducible cells form cytoplasmic foci but not YFP-ALK, and the tracking analysis of EML4-ALK V3 foci.

A Inducible Beas2B V1, V3 and PVX (empty vector) cells were stained with either anti-ALK (green) or anti-GFP (green), anti-α-tubulin (red) and DAPI (blue). Scale bars, 10 μm; magnified views of a selected area are shown.

B Violin plot showing the number of cytoplasmic foci per cell. Data represent 40–50 counts from three biological replicates. ****$P < 0.0001$ in comparison with Beas2B PVX (empty vector) by one-way ANOVA.

C HEK293 cells were transfected with YFP-ALK 1,058–1,620 for 48 h before fixation and staining with anti-GFP (green), anti-α-tubulin (red) and DAPI (blue). Scale bars, 10 μm.

D Violin plot representing the number of cytoplasmic foci per cell. Data represent counts from at least 20 cells from two biological replicates. Not significant (ns) in comparison with YFP EML4-ALK 1,058–1,620 by unpaired *t*-test.

E Track classification of YFP-EML4-ALK V3 foci in the cytoplasm of HEK293. Duration of the movie was 12 s. Scale bar, 1 μm. Each coloured and numbered trajectory indicates the movement of an individual droplet.

F Heatmap representing the number of split and merge events in each trajectory.

G Plot of foci maximum speed (μm/s) versus foci diameter (μm). The best-fit line shows correlation with 95% confidence interval.

H Plot displays the total distance (μm) and displacement (μm) of each trajectory. Colours and numbers relate to the tracks shown in (E).

Figure EV2. Association of signalling protein with active EML4-ALK V1 and V3 cytoplasmic foci in inducible Beas2B cells.

A, B Inducible Beas2B V1 and V3 cells were stained with anti-ALK (green), anti-GRB2 (red), anti-SOS1 (red), anti-pC-KIT^{Y721} (red), anti-PI3K p85β or anti-pPLCγ2^{Y759} and DAPI (blue). Scale bars, 10 μm; magnified views of a selected area are shown.

C–G Intensity profiles showing colocalisation between endogenous GRB2, SOS1, pC-KIT^{Y721}, PI3K p85β or pPLCγ2^{Y759} and ALK staining in different cell lines. *R* (Pearson's correlation coefficient) measures the correlation between the indicated proteins and ALK signals. Pearson's measurements are from 20 foci of 10–20 cells for each antibody combination. Data in all whisker plots represent counts from at least 10 cells, $n = 3$. The central dashed band indicates the minimum Pearson *R* value (0.5) required for colocalisation. All whisker boxplots indicate the minimum and maximum Pearson *R* values of each cell line. *$P < 0.5$, **$P < 0.01$, ***$P < 0.001$, ****$P < 0.0001$ in comparison of each cell line by one-way ANOVA.

 © 2021 The Authors

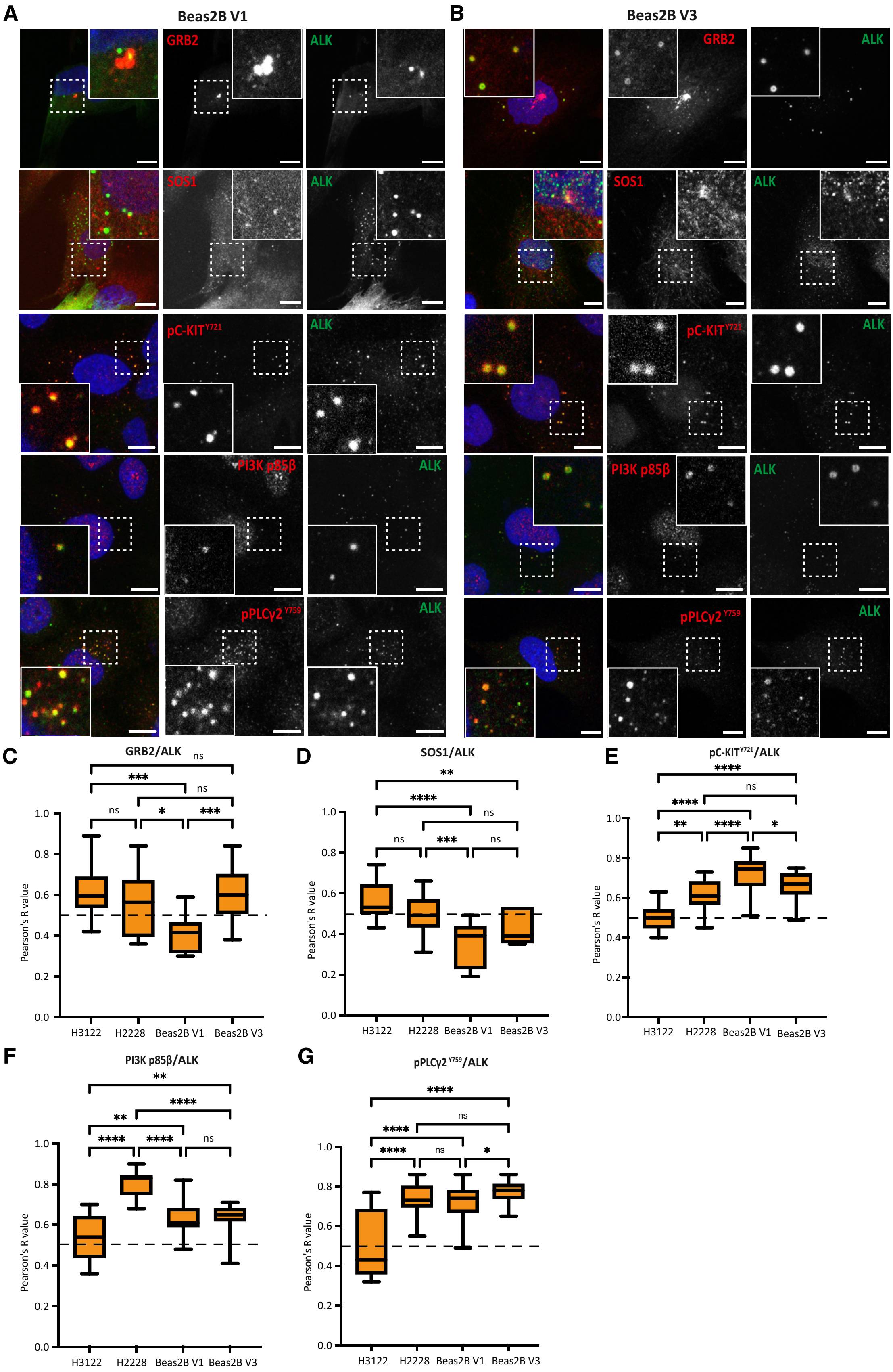
A
Beas2B V1
GRB2
ALK
SOS1
ALK
pC-KIT Y721
ALK
PI3K p85β
ALK
pPLCγ2 Y759
ALK
B
Beas2B V3
GRB2
ALK
SOS1
ALK
pC-KIT Y721
ALK
PI3K p85β
ALK
pPLCγ2 Y759
ALK
C
GRB2/ALK
D
SOS1/ALK
E
pC-KIT Y721/ALK
F
PI3K p85β/ALK
G
pPLCγ2 Y759/ALK
Pearson's R value
H3122
H2228
Beas2B V1
Beas2B V3
ns
*
**
***
****

Figure EV2.

Figure EV3. Loss of signalling proteins upon 1,6-hexanediol and ALK inhibitors.

A H3122 and H2228 cells were treated with either 5% or 10% 1,6-hexanediol for 5 min. DMSO was used a control. Lysates were analysed for the phosphorylation and expression of the indicated antibodies. GAPDH was used as a loading control. Data represent of $n = 2$ experiments.

B H2228 and Beas2B V3 cells were treated with ALK inhibitors for 4 h before fixation and staining with GRB2 and ALK antibodies for proximity ligation assay (PLA). Nuclei are indicated by DAPI staining (blue). Red foci indicate GRB2/ALK protein complexes. Single ALK antibody staining was used as a control for PLA interactions. Scale bars, 10 µm.

C, D Bar graphs representing the number of PLA foci per cell from B, GRB2/ $pALK^{Y1604}$ PLA signals in H2228 cells and Beas2B V3. Data represent counts from at least 30 cells, $n = 3$. Error bar represents SD of three biological replicates. ****$P < 0.0001$ in comparison with DMSO (empty vector) by one-way ANOVA.

Source data are available online for this figure.

© 2021 The Authors

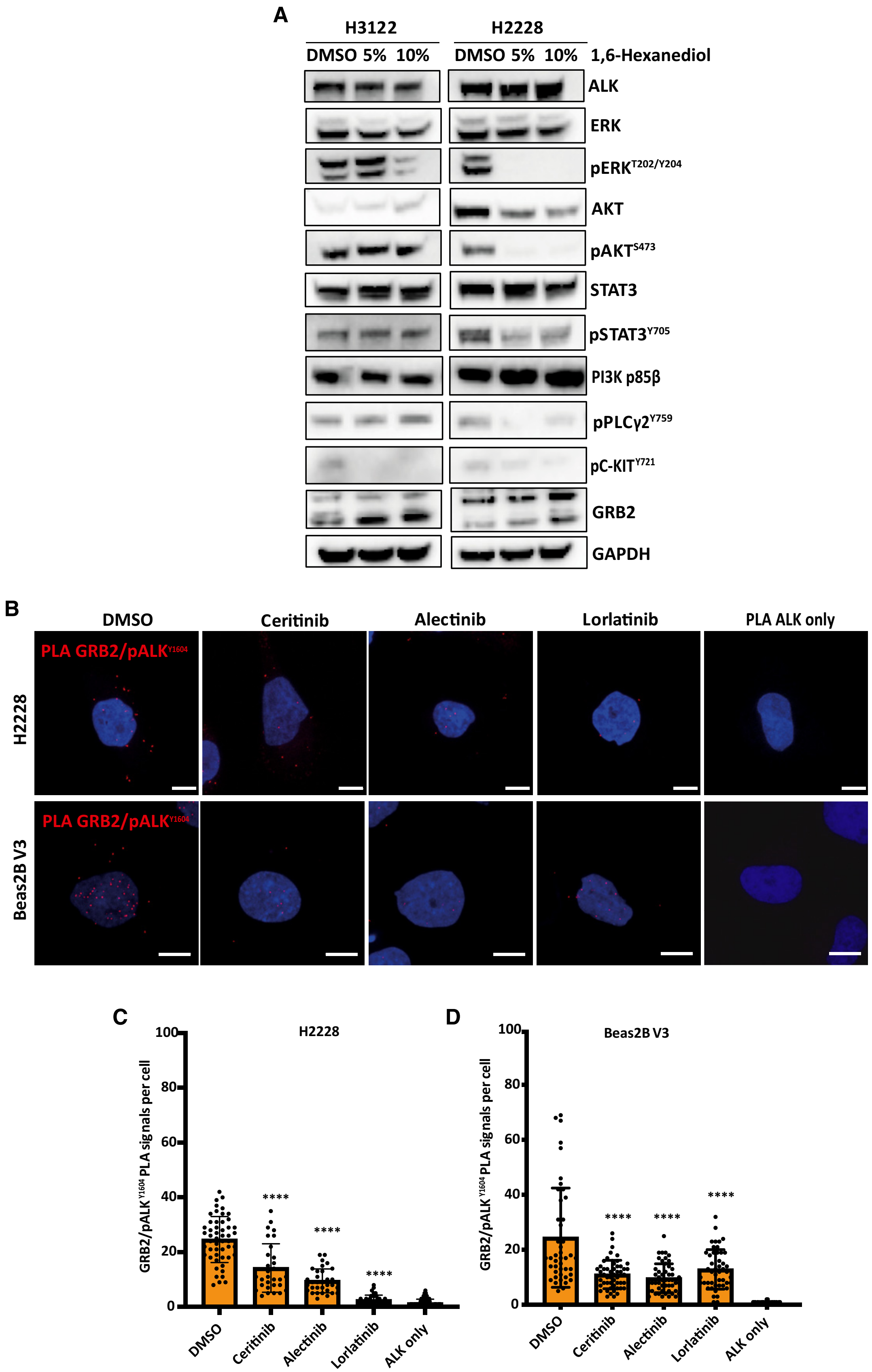
A
H3122
H2228
DMSO 5% 10%
DMSO 5% 10%
1,6-Hexanediol
ALK
ERK
pERKT202/Y204
AKT
pAKTS473
STAT3
pSTAT3Y705
PI3K p85β
pPLCγ2Y759
pC-KITY721
GRB2
GAPDH
B
DMSO
Ceritinib
Alectinib
Lorlatinib
PLA ALK only
PLA GRB2/pALKY1604
H2228
PLA GRB2/pALKY1604
Beas2B V3
C
H2228
GRB2/pALKY1604 PLA signals per cell
100
80
60
40
20
0
****
****
****
DMSO
Ceritinib
Alectinib
Lorlatinib
ALK only
D
Beas2B V3
GRB2/pALKY1604 PLA signals per cell
100
80
60
40
20
0
****
****
****
DMSO
Ceritinib
Alectinib
Lorlatinib
ALK only

Figure EV3.

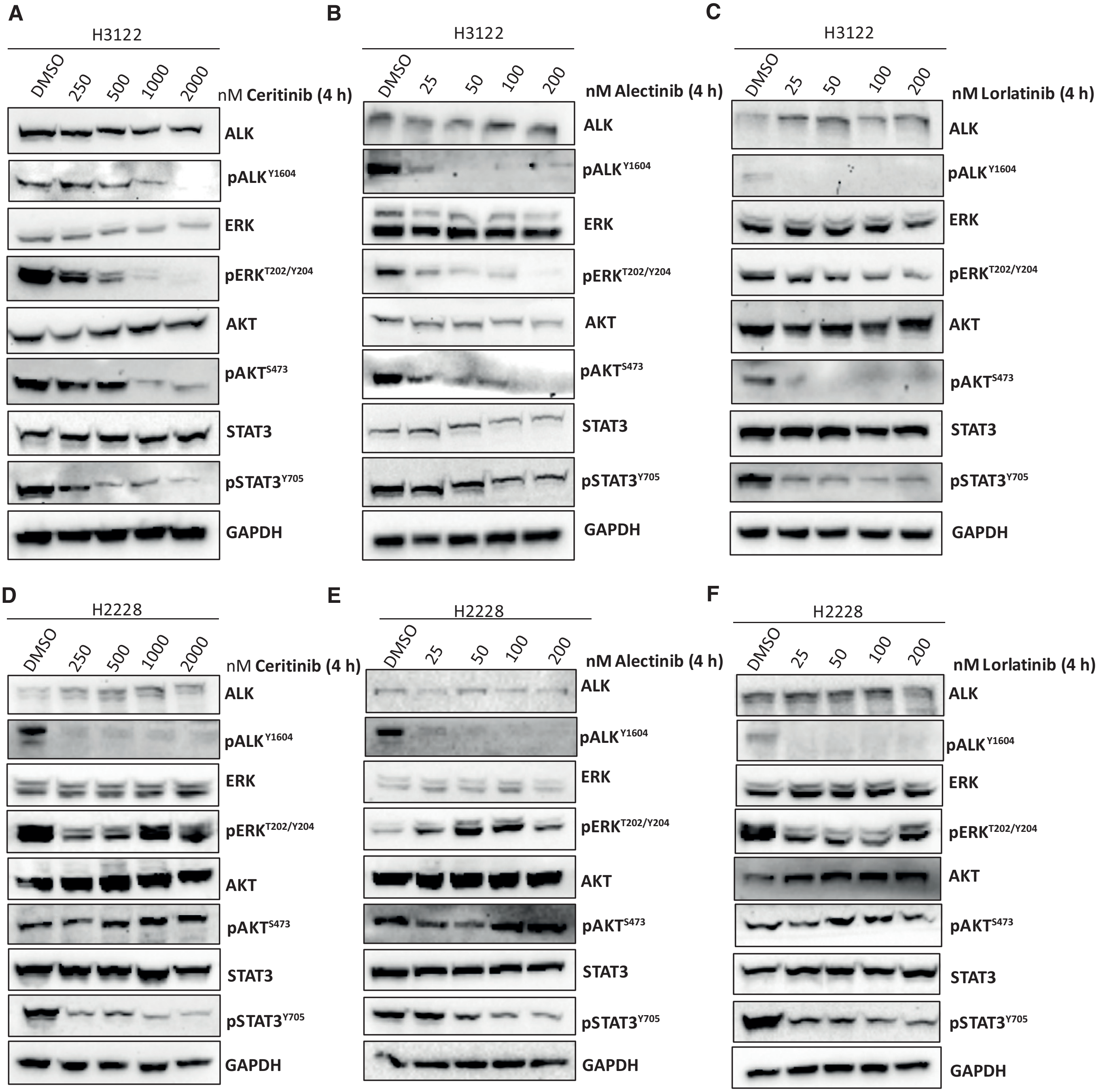

Figure EV4. Effects of ALK inhibition on signalling pathways in H3122 and H2228 cells.

A–C Representative Western blots of serum-starved H3122 cells treated with increasing doses of (A) ceritinib, (B) alectinib or (C) lorlatinib for 4 h. An equal volume of DMSO was used as control. Lysates were analysed by Western blotting and immunostaining for the proteins indicated and for their phosphorylation at the sites indicated. GAPDH was used as a loading control. Data represent of $n = 2$ experiments.

D–F Serum-starved H2228 cells were treated with increasing doses of (D) ceritinib, (E) alectinib or (F) lorlatinib for 4 h. Lysates were analysed by Western blotting and immunostaining with the indicated antibodies. GAPDH was used as a loading control. Data represent of $n = 2$ experiments.

Source data are available online for this figure.

© 2021 The Authors

Figure EV5. Effects of ALK inhibitors on localisation of EML4-ALK V1 and V3 harbouring cell lines.

A Beas2B V3 cells were induced with doxycycline for 72 h and treated with ALK inhibitors or DMSO for 4 h. Cells were fixed and stained with anti-ALK (green), anti-α-tubulin (red), and DAPI (blue). Scale bars, 10 μm; magnified views of a selected area are shown.

B H2228 cells treated with ALK inhibitors or DMSO for 4 h. Cells were fixed and stained with anti-ALK (green), anti-α-tubulin (red) and DAPI (blue). Scale bars, 10 μm; magnified views of a selected area are shown.

C, D Violin plots representing the number of cytoplasmic foci per cell from (A) and (B), respectively. Data represent measurements taken from at least 20 cells, $n = 3$. ****$P < 0.0001$ in comparison with DMSO in Beas2B V3 and H2228 by one-way ANOVA.

E H3122 cells and Beas2B V1 cells were treated with ALK inhibitors or DMSO for 4 h. Cells were fixed and stained with anti-ALK (green) or anti-GFP (green), anti-α-tubulin (red), and DAPI (blue). Scale bars, 10 μm; magnified views of a selected area are shown.

F, G Violin plots representing the number of cytoplasmic foci per cell from (E). Data represent measurements taken from at least 10 cells, $n = 3$. **$P < 0.01$, ****$P < 0.0001$ in comparison with DMSO by one-way ANOVA.

H, I Intensity profiles showing colocalisation between YFP-EML4-ALK V1 or V3 WT or KD and microtubules −/+ ALK inhibitors. *R* (Pearson's correlation coefficient) measures the correlation between YFP and α-tubulin signals. Pearson's measurements from 20–30 cells for each construct.

© 2021 The Authors

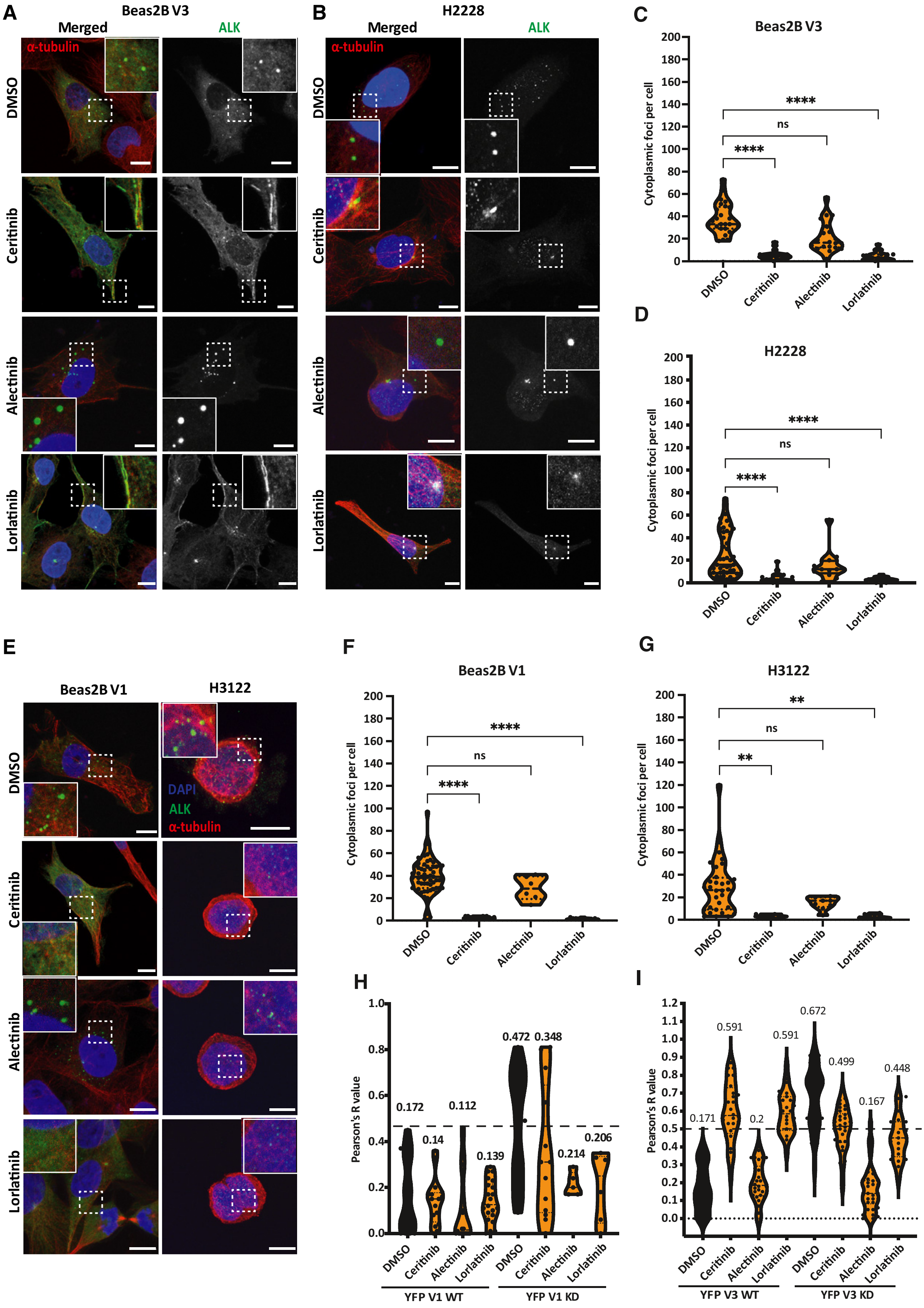

Figure EV5.

 © 2021 The Authors
