# Supplementary figures and images for: Phase‐separated foci of EML4‐ALK facilitate signalling and depend upon an active kinase conformation
Source: EMBO Rep. 2021 Oct 18;22(12):e53693. doi: 10.15252/embr.202153693 (PMC8647013; doi:10.15252/embr.202153693)

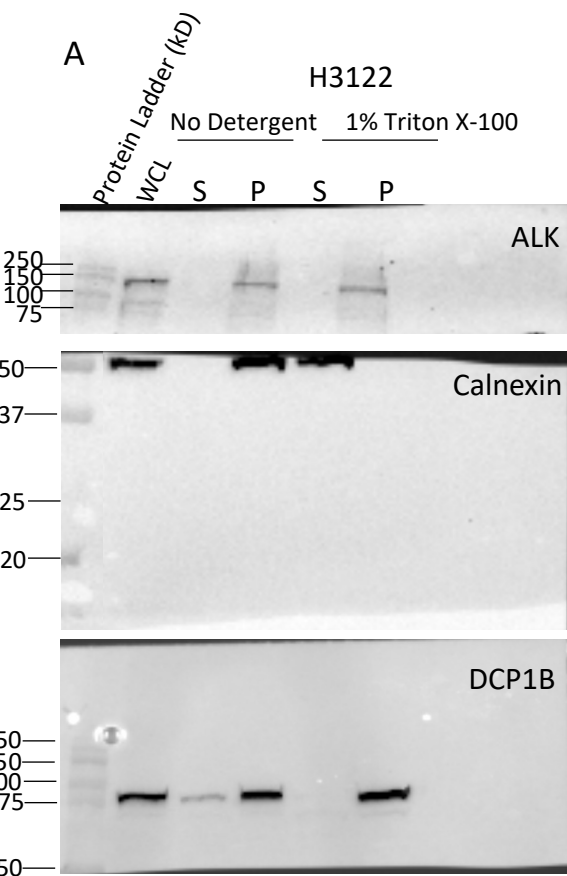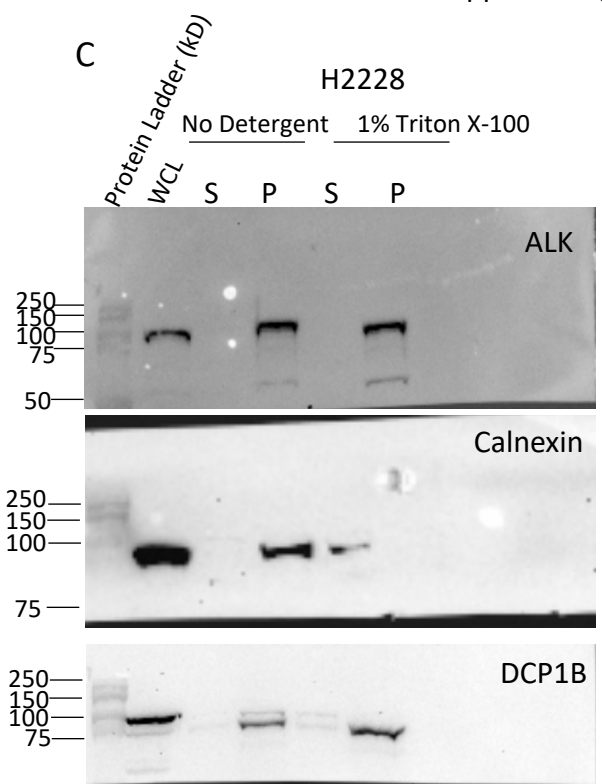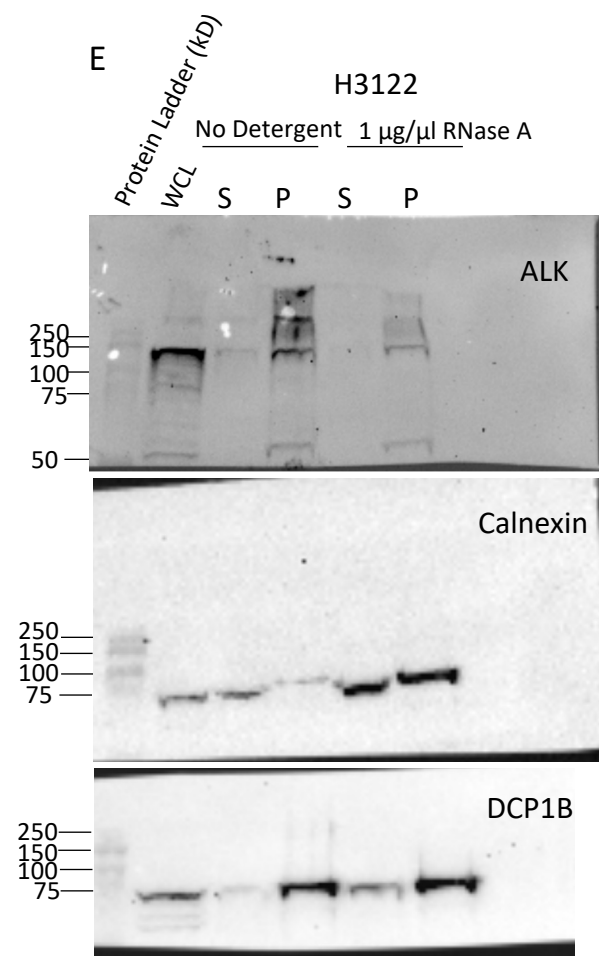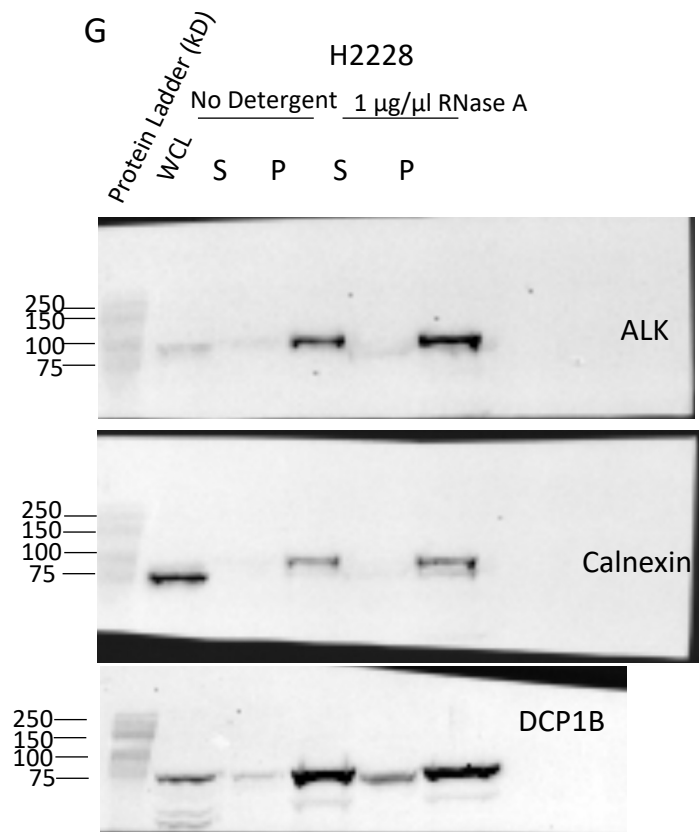

Supplement: Supplementary file 3 — Source Data for Expanded View and Appendix [file EMBR-22-e53693-s007.zip › Appendix_and_EV_Source_Data-sd/Appendix_FigureS1_Source_data.pdf]

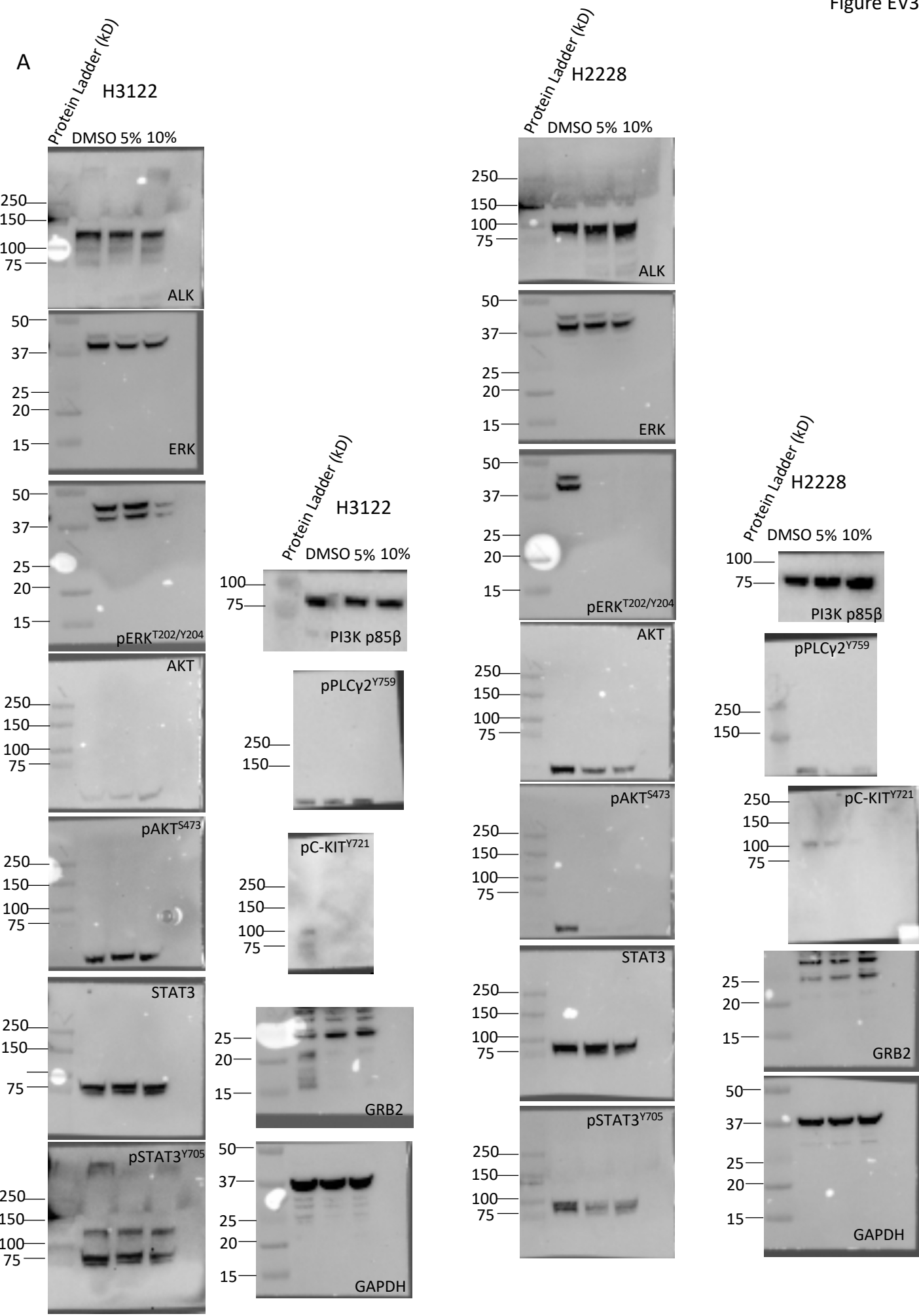

Supplement: Supplementary file 3 — Source Data for Expanded View and Appendix [file EMBR-22-e53693-s007.zip › Appendix_and_EV_Source_Data-sd/Source data Figure EV3.pdf]

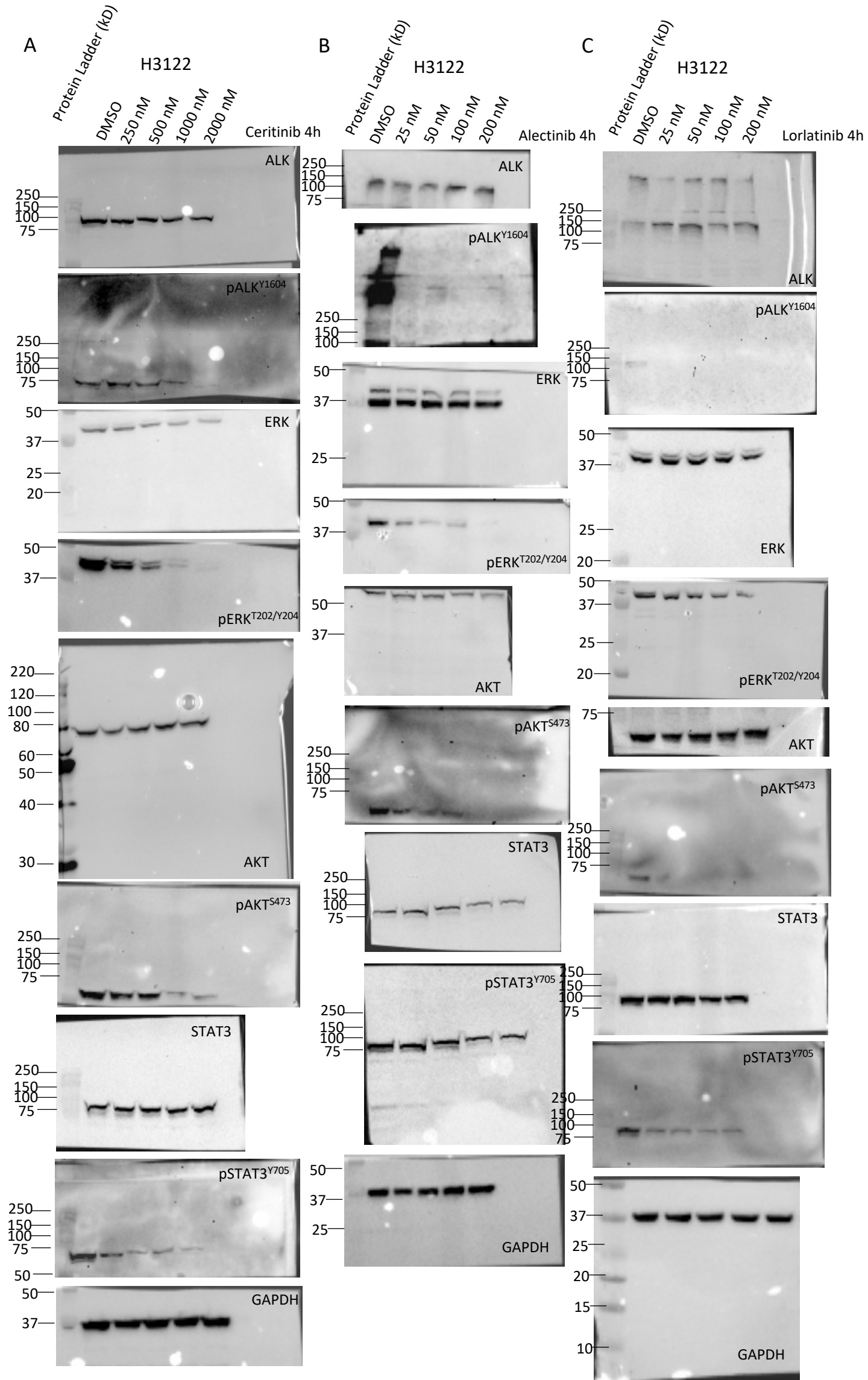

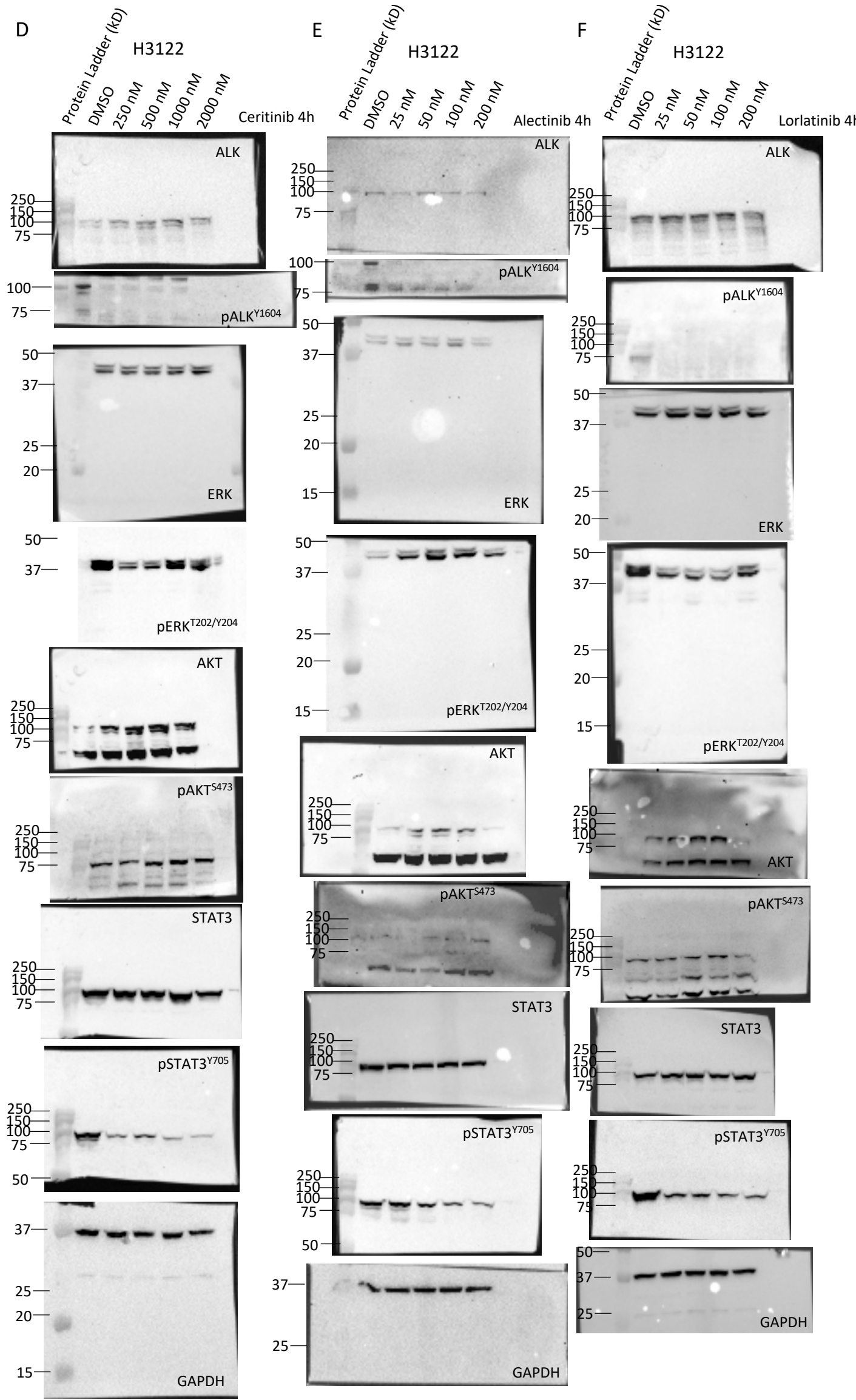

Supplement: Supplementary file 3 — Source Data for Expanded View and Appendix [file EMBR-22-e53693-s007.zip › Appendix_and_EV_Source_Data-sd/Source data Figure EV4.pdf]

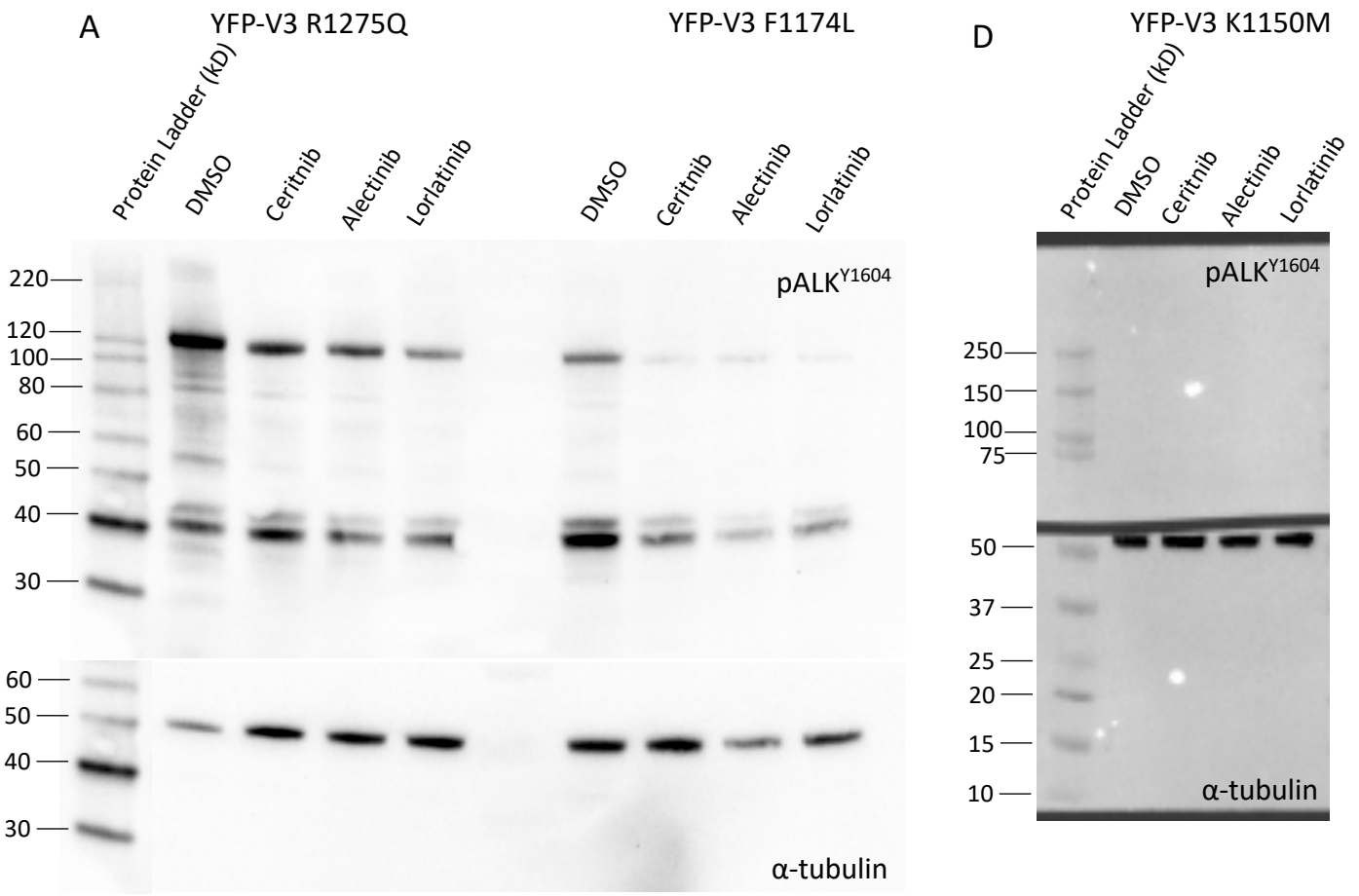

Supplement: Supplementary file 3 — Source Data for Expanded View and Appendix [file EMBR-22-e53693-s007.zip › Appendix_and_EV_Source_Data-sd/Appendix_FigureS4_Source_Data.pdf]

Figure 2

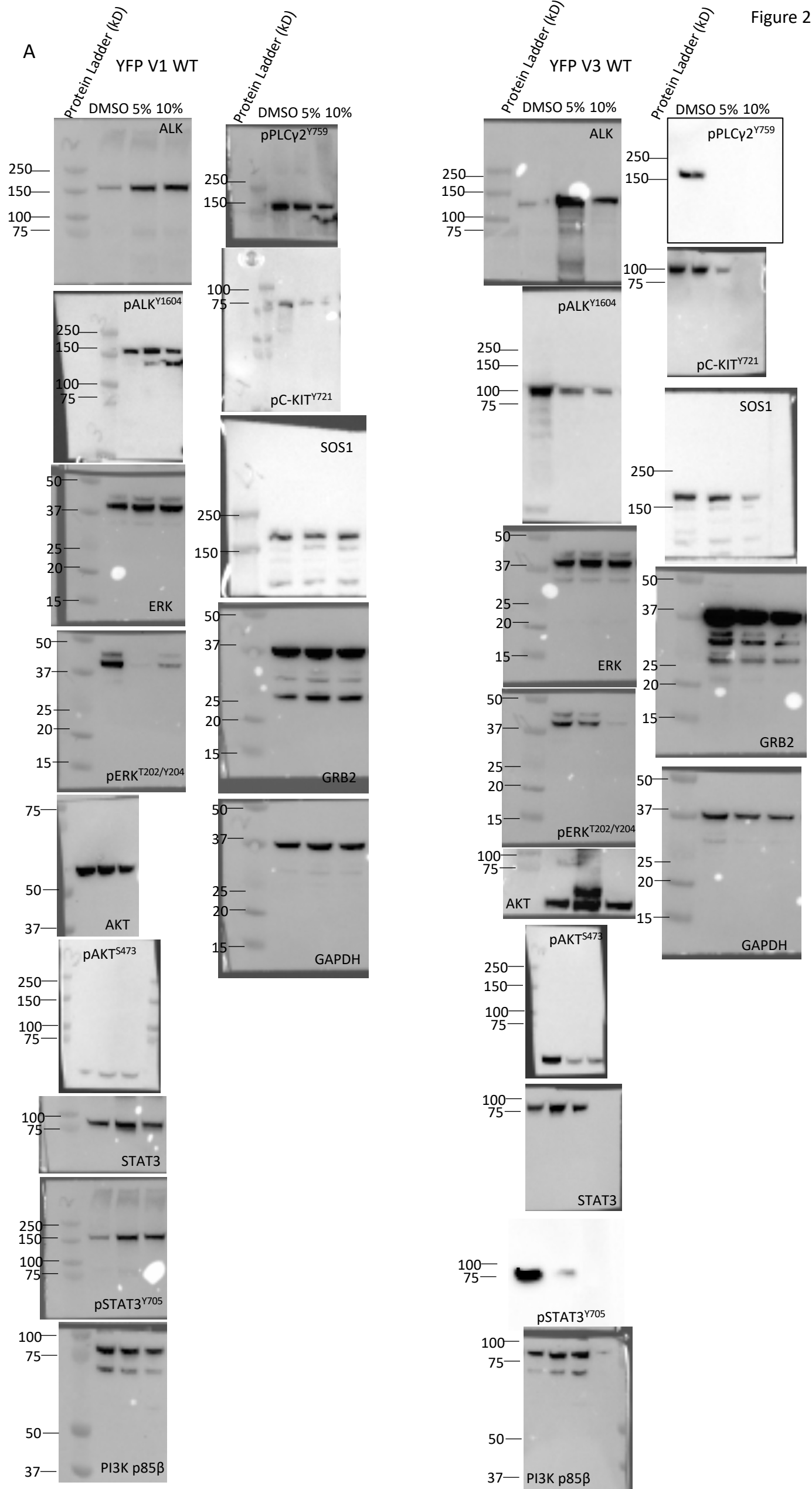

Supplement: Supplementary file 14 — Source Data for Figure 2 [file EMBR-22-e53693-s002.pdf]

Figure 3

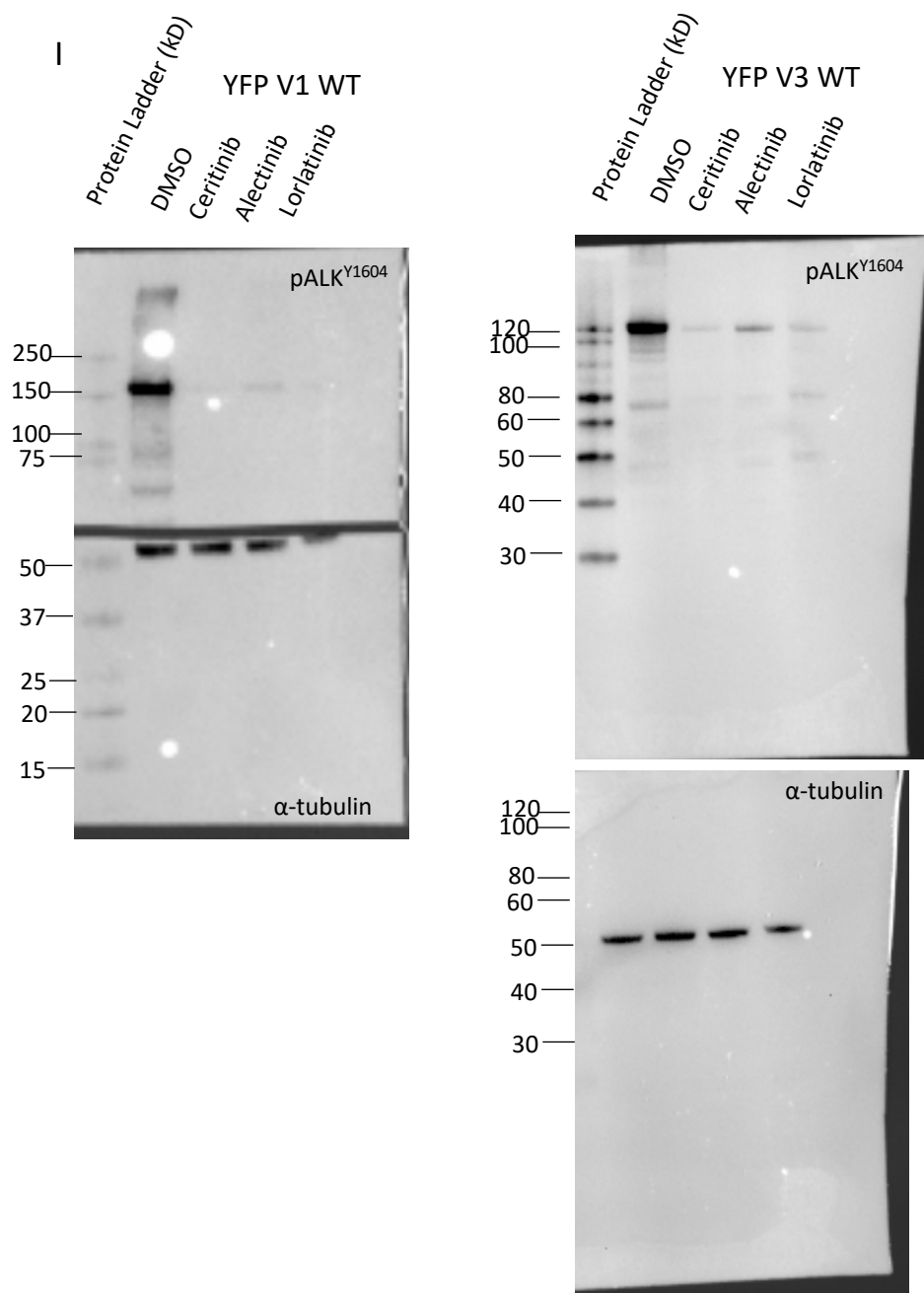

Supplement: Supplementary file 15 — Source Data for Figure 3 [file EMBR-22-e53693-s009.pdf]
