# Supplementary material for: Phase‐separated foci of EML4‐ALK facilitate signalling and depend upon an active kinase conformation
Source: EMBO Rep. 2021 Oct 18;22(12):e53693. doi: 10.15252/embr.202153693 (PMC8647013; doi:10.15252/embr.202153693)
Supplement: Supplementary file 4 — Movie EV1 [file EMBR-22-e53693-s003.zip › Movie EV1.docx]

**Movie EV1. Time-lapse imaging of EML4-ALK V3 WT droplet formation in HEK293 cells**

Time-lapse imaging of HEK293 cells transfected with YFP-EML4-ALK V3 WT. 12 z-sections of 1 μm step size were captured every second. Time is shown in seconds.
