# Supplementary material for: Phase‐separated foci of EML4‐ALK facilitate signalling and depend upon an active kinase conformation
Source: EMBO Rep. 2021 Oct 18;22(12):e53693. doi: 10.15252/embr.202153693 (PMC8647013; doi:10.15252/embr.202153693)
Supplement: Supplementary file 5 — Movie EV2 [file EMBR-22-e53693-s015.zip › Movie EV2.docx]

**Movie EV2. Time-lapse imaging of EML4-ALK V3 kinase dead (KD) in HEK293 cells**

Time-lapse imaging of HEK293 cells transfected with YFP-EML4-ALK V3 D1270N. 25 z-sections of 1 μm step size were captured every second. Time is shown in seconds.
