# Supplementary material for: Phase‐separated foci of EML4‐ALK facilitate signalling and depend upon an active kinase conformation
Source: EMBO Rep. 2021 Oct 18;22(12):e53693. doi: 10.15252/embr.202153693 (PMC8647013; doi:10.15252/embr.202153693)
Supplement: Supplementary file 11 — Movie EV8 [file EMBR-22-e53693-s001.zip › Movie EV8.docx]

**Movie EV8. Time-lapse imaging of EML4-ALK V3 WT by FRAP**

Time-lapse imaging of HEK293 cells transfected with YFP-EML4-ALK V3 WT and a ROI of 40x40 µm was photobleached at 100% argon 488 nm and 100% 405 nm laser power simultaneously. A second ROI of 40x40 µm was used as control (no photo-bleach) to represent the background. Single frames were captured every second. Time is shown in seconds.
