# Supplementary material for: Phase‐separated foci of EML4‐ALK facilitate signalling and depend upon an active kinase conformation
Source: EMBO Rep. 2021 Oct 18;22(12):e53693. doi: 10.15252/embr.202153693 (PMC8647013; doi:10.15252/embr.202153693)
Supplement: Supplementary file 16 [file EMBR-22-e53693-s012.pdf]

EMBO reports

Phase-separated foci of EML4-ALK facilitate signalling and depend upon an active kinase conformation

Josephina Sampson, Mark Richards, Jene Choi, Andrew Fry, and Richard Bayliss
DOI: 10.15252/embr.202153696

Corresponding author(s): Richard Bayliss (r.w.bayliss@leeds.ac.uk)

Review Timeline:

Transfer from Review Commons:	24th Jul 21
Editorial Decision:	5th Aug 21
Revision Received:	9th Sep 21
Accepted:	14th Sep 21

Editor: Achim Breiling

Transaction Report: This manuscript was transferred to EMBO Reports following peer review at Review Commons and The EMBO Journal.

(Note: With the exception of the correction of typographical or spelling errors that could be a source of ambiguity, letters and reports are not edited. Depending on transfer agreements, referee reports obtained elsewhere may or may not be included in this compilation. Referee reports are anonymous unless the Referee chooses to sign their reports.)

1st Editorial Decision 5th Aug 2021

Dear Prof. Bayliss,

Thank you for the transfer of your revised manuscript to our editorial offices. I went through your manuscript, the point-by-point response of the original revised manuscript submitted to The EMBO Journal and your further response to the remaining concerns of referee #2. Looking through these, I think that the concerns of all three referees have been adequately addressed and the manuscript is suitable for publication in EMBO reports.

Before we can proceed with formal acceptance, I have these editorial requests I ask you to address in a final revised manuscript:

- Please add a formal 'Data Availability Section' to the manuscript. This refers to large datasets that have been submitted to a public database. If no primary datasets have been deposited (as seems the case here), please state this in that section (e.g. 'No primary datasets have been generated or deposited').

- Please order the manuscript sections like this (using these section names):
Title page - Abstract - Introduction - Results - Discussion - Materials and Methods - Data availability section - Acknowledgements - Author contributions - Conflict of interest statement - References - Figure legends - Expanded View Figure legends - tables.

- Please make sure that all the funding information is also entered into the online submission system and is complete and similar to the one in the manuscript text file.

- There are 10 movie legends in the manuscript, but no movie files have been uploaded. Please upload the movie files separately, naming these 'Movie EVx', ZIPed together with their corresponding title and legend as text file. Finally, please check that all the movies are called out (and are correctly called out using 'Movie EVx') and finally remove the movie legends from the manuscript main text.

- Some microscopic images miss the scale bars (e.g. in Fig. 3L), whereas and many scale bars presently are too thin. Please add scale bars of similar style and thickness to all the microscopic images (main, EV and Appendix images), using clearly visible black or white bars (depending on the background). Please place these in the lower right corner of the images. Please do not write on or near the bars in the image but define the size in the respective figure legend.

- EV figures 2 and 3 are labeled 'Figure S3' and 'Figure S4'. Please change this.

- The time-lapse images in Fig. 1E all appear identical, i.e. this seems to be always the same image. Please check/explain.

- Fig 3 has no panels F and J (although there are panels G, H, I, K, L). Please check (also the callouts).

- Fig. EV5 has no panels F, G, J (although there are panels H, I, K, L). Please check (also the callouts).

- Please align the label for panel I in Fig 3 and for panel H in Fig. EV1 with the other panel labels.

- Could the data in panel EV1H be shown with the same weight (thickness - 1 and 7 are much thinner).

- Please move Tables 1 & 2 with their title and legends after figure legends, at the very end of the manuscript text.

- Please make sure that the number "n" for how many independent experiments were performed, their nature (biological versus technical replicates), the bars and error bars (e.g. SEM, SD) and the test used to calculate p-values is indicated in the respective figure legends (also of the EV and Appendix figures), and that statistical testing has been done where applicable. Please avoid phrases like 'independent experiment', but clearly state if these were biological or technical replicates. If statistical testing was done but there is no significant difference, please also mark this in all the diagrams (n.s.).

- As the Western blots shown are significantly cropped, could you provide the source data for all the blots (main figures, EV figures and Appendix figures). The source data will be published in a separate source data file online along with the accepted manuscript and will be linked to the relevant figure. Please submit the source data (scans of entire blots) together with the revised manuscript. Please include size markers for scans of entire blots, label the scans with figure and panel number and send one PDF file per figure. The images for the Appendix should be combined in one file.

- Please make sure that the references are formatted according to our journal style (we need et al. for references with more than 10 authors). See:
http://www.embopress.org/page/journal/14693178/authorguide#referencesformat

- Finally, please find attached a word file of the manuscript text (provided by our publisher) with changes we ask you to include in your final manuscript text, and some queries, we ask you to address. Please provide your final manuscript file with track changes, in order that we can see the modifications done.

In addition, I would need from you:
- a short, two-sentence summary of the manuscript (not more than 35 words).
- two to four bullet points highlighting the key findings of your study.
- a schematic summary figure (in jpeg or tiff format with the exact width of 550 pixels and a height of not more than 400 pixels) that can be used as a visual synopsis on our website.

I look forward to seeing the final revised version of your manuscript when it is ready. Please let me know if you have questions regarding the revision.

Kind regards,

Achim Breiling
Editor
EMBO Reports

<u>**Review Commons: Revised manuscript for publication as a Research article**</u>

To reflect the enhanced breadth and depth of the revised manuscript, we have amended the title to:

Phase-separation of EML4-ALK facilitates signalling and depends upon an active kinase conformation

by Josephina Sampson, Mark W. Richards, Jene Choi, Andrew M. Fry and Richard Bayliss

Referee comments are in *black italic*; our response is in blue standard font.

Response to Reviewer #1 (Evidence, reproducibility and clarity (Required)):

In this manuscript Sampson and colleagues report that the ALK/EML4 variant 3 partition into liquid droplets in the cytoplasm and this partition is dependent on the conformation rather than the catalytic activity of the ALK kinase. They demonstrate that the droplets contain proteins such as GRB2 and SOS1. TKIs, including ceritinib, alectinib and lorlatinib affect the localisation of the ALK/EML4 V3 by dissolving or maintaining these droplets. Particularly, while ceritinib and lorlatinib dissolved the ALK/EML4 V3 droplets, alectinib induced the formation of liquid droplets. Furthermore, the authors demonstrate that the ALK kinase activity is fundamental for droplets' formation.
The manuscript is interesting and novel and experiments are well performed.

We thank the referee for noting the novelty and importance of our study, and highlighting that the study was well performed and interesting.

However, few points need to be addressed as highlighted below:
In fig 2 would be more clear to see color images for pALK in green and GRB2/SOS1 in red, not only the merged images.

In the revised version, we have extended our colocalization experiments between ALK and several other signalling proteins including GRB2, SOS1, pC-KIT^{Y721}, PI3K p85β and pPLCγ2^{Y759} in patient-derived H3122 (V1) and H2228 (V3) and in the inducible Beas2B cells. We have included the green and red individual images with zoomed areas emphasizing the colocalization of the two proteins. The revised figure 2 and supplementary S3 provide a detailed representation of the colocalization between EML4-ALK V1/V3 foci and signalling proteins. These set of data highlight the existence of those proteins in EML4-ALK foci.

The majority of the experiments have been performed in HEK293. It is important to confirm droplets formation in ALK negative NSCLC cell lines.

We highlight the presence of EML4-ALK foci in several cell models including the endogenous EML4-ALK NSCLC H3122 (V1) and H2228 (V3) cell lines, as well as in inducible (Beas2B) and overexpressed (HEK293) cell lines. In addition, we have added extra controls including non-transfected and ALK domain only (1058-1620) HEK293 cells, as well as PVX (empty vector) Beas2B as a control for the inducible model system.

As stated in the discussion authors propose less metastatic spread in patients treated with alectinib. However, experiments that support this hypothesis are missing. Migration and/or invasion assay would be helpful to verify this hypothesis.

We decided to remove this statement on metastatic spread and alectinib from the discussion. We wanted to have a focused discussion that describes in depth the molecular mechanisms of the EML4-ALK foci formation

and their importance in signal transduction. Our key findings are summarized in Figure 8, where we described in detail the stoichiometric mismatch of trimeric EML4 with ALK dimerization for the foci formation and the recruitment of proteins to facilitate cellular signalling.

Reviewer #1 (Significance (Required)):
The study is quite novel, I did not find anything related to the topic. My expertise is on NSCLC.

Response to Reviewer #2 (Evidence, reproducibility and clarity (Required)):

Phase-separation of EML44-ALK variant 3 is dependent upon an active ALK conformation

The current submission aims to gain a detailed understanding of mechanisms of ALK-fusion protein localisation / conformation / activation with models of a common, aggressive EML4-ALK fusion variant EML4-ALK V3 found in non small lung cancer (NSCLC). Experimental methods include the use of a single EML4-ALK lung cancer cell line (H2228), over-expression models of EML4-ALK V3 (in Beas2B (tet inducible) and 293 cells), genetically modified tagged, mutant & deletion EML44-ALK V3 constructs and also a range of ALKi (crizotinib, ceritinib, alectinb & lorlatinib).

Essentially the EML4-ALK V3 can be found in 2 subcellular localisations, either bound to microtubules or in lipid droplets. Data is presented which is suggestive that EML4 ALK V3 in lipid droplets co-localises with components of the RAS/MAPK pathway (GRB2 & SOS1). Dimerisation of EML4-ALK V3 protein drives lipid droplet localisation, the active ALK conformation drives lipid droplet localisation, most ALKi (except alectinib which forces ALK to adopt an active conformation) block lipid droplet localisation of wild type EML4 ALK V3 but not the F1174 mutant EML4-ALK V3 & constitutively active ALK mutants. The results are used to develop a model to explain the role of differing ALK mutations / ALK inhibitors have on ALK activation & autophosphorylation.

We thank the referee for noting the novelty and importance of our study.

Comments:

1.The majority of the data has been generated using overexpression of tagged EML4-ALK V3 (including mutants and deletion constructs) in 293 cells. There is supporting data from one patient derived EML4 ALK V3 cell line and tetracycline induced expression in a lung cell model. The strength of the observations made & and conclusions drawn from the tagged EML4-ALK V3 constructs would be increased by additional work with the H2228 cell line and/or additional patient derived lung cancer cell lines (these may be difficult to come by).

Indeed, it is difficult to get additional patient derived lung cancer cell lines but will be attractive for the future. We have shown EML4-ALK foci formation using several models such as overexpressed EML4-ALK tagged in HEK293, patient derived EML4-ALK V1 and V3 cell lines and tetracycline induced expression Beas2B cell line. We believe these set of data confirm the presence of EML4-ALK foci. It should be highlighted those two recent studies from Tupule et al and Qin et al recorded the presence of EML4-ALK foci in their cellular and mouse models (1, 2).

1. Tulpule, A., Guan, J., Neel, D. S., Allegakoen, H. R., Lin, Y. P., Brown, D., Chou, Y. T., Heslin, A., Chatterjee, N., Perati, S., Menon, S., Nguyen, T. A., Debnath, J., Ramirez, A. D., Shi, X., Yang, B., Feng, S., Makhija, S., Huang, B. and Bivona, T. G. (2021) 'Kinase-mediated RAS signaling via membraneless cytoplasmic protein granules', *Cell,* 184(10), pp. 2649-2664.e18.

2. Qin, Z., Sun, H., Yue, M. *et al.* Phase separation of EML4–ALK in firing downstream signaling and promoting lung tumorigenesis. *Cell Discov* **7,** 33 (2021). https://doi.org/10.1038/s41421-021-00270-5

2.Fundamentally this is a nice set of experiments and observations. However I don't get a real sense of the physiologic, pharmacologic and translational relevance of the findings. The EML4-ALK V3 is an aggressive variant associated with a metatstatic phenotype, which is more resistant to & responds less well to ALKi treatment. However the experimental results aren't substantively related back to these statements. It's not clear whether the divergent results on the EML4-ALK V3 localisation dependent on ALKi binding (alectinib versus crizotinib / ceritinib / lorlatinib) are ultimately an interesting but predominantly laboratory based observation in an overexpression model with 293 cells or are perhaps more fundamentally linked to ALKi response / resistance in lung cancer.

Our observation of EML4-ALK foci is not just in 293 cells, but also in lung cancer cell lines derived from patients, as well as other model cell lines. Similar observations have recently been reported in two papers (Tupule et al; Qin et al.), and so the physiological relevance of these foci is clear. We and others have shown that these foci are enriched in signalling molecules and, critically, we show that dissolving the V3 foci blocks signalling. This to us is a key finding of translational significance because it provides a new route to targeting EML4-ALK V3 – a priority for this more aggressive variant of the disease. The key finding of pharmacological significance is that the ALK inhibitors can elicit different responses in the foci, resulting in differential cellular localisation of these proteins. It remains to be seen if these differences at a cellular level will translate to differences on patient response or outcomes, but we feel it is important that this question is now asked so that it can be answered through longitudinal studies on patients. Of course, the fact that we have a working model of the molecular mechanism of foci formation, and of the differences between inhibitors, means that we can develop strategies for exploiting these mechanisms in the development of therapies to synergise with ALK inhibitors.

3.The transition between microtubule bound versus lipid droplet is clearly demonstrated yet in the YFP tagged overexpression model (figure 1A), really only a very small proportion of the YFP tagged EML4-ALK V3 ends up in lipid droplets compared to bound to microtubules (although these are static images). This may be an artefact of the overexpression. Second in comparing the kinase dead EML4-ALK V3 to the wild type EML4-ALK V3, whilst there are clearly very few / no lipid droplets in the kinase dead, the most notable feature is the very different alpha-tubulin staining patterns between kinase active versus kinase dead ALK.

To further emphasize the presence of these proteins primarily in liquid droplets, we have included further examples of H2228, HEK293 and H3122 cell lines to the figures and supplementary figures. As well as static figures, we include movies of the HEK293 EML4-ALK V3 foci dynamics. Several other approaches are used to show the presence of liquid droplets – eg. FRAP, treatment with compounds that dissolve specific types of condensate. ALK inhibitors do not affect the microtubule network in control or V1 cells, and therefore these effects are driven by the binding of V3 to the microtubules, consistent with published data (1). The relocalisation of V3 to microtubules upon ALK inhibition (or in kinase dead) is indeed striking and is driven by the microtubule-binding region of the EML4 portion (as we previously published – (2)). New in the revised version, we pin down the region in V1 that *prevents* binding to microtubules – this is the blade12N subdomain – and we present a model to explain its behaviour.

1. O'Regan L, Barone G, Adib R, Woo CG, Jeong HJ, Richardson EL, Richards MW, Muller PAJ, Collis SJ, Fennell DA, Choi J, Bayliss R, Fry AM. EML4-ALK V3 oncogenic fusion proteins promote microtubule stabilization and accelerated migration through NEK9 and NEK7. J Cell Sci. 2020 May 11;133.
2. Richards MW, O'Regan L, Roth D, Montgomery JM, Straube A, Fry AM, Bayliss R (2015) Microtubule association of EML proteins and the EML4-ALK variant 3 oncoprotein require an N-terminal trimerization domain. Biochem. J. 467:529-536.

4.The ALKi experiments have been conducted using single concentrations of each ALKi [Ceritinib(0.5 µmoles/L); Alectinib (0.1 µmoles /L); Lorlatinib (0.1 µmoles /L) - are these doses correct?] with over-expression of tagged EML4-ALK V3 in 293 cells. This is clearly a tractable experimental system but I think the strength of the observations would be increased by additional work.

Yes, the above ALK inhibitors were used at those concentrations. In the revised manuscript, we have included EML4-ALK V1 data and ALK inhibitor treatments in the inducible Beas2B and patient-derived cell lines. These set of data confirmed the localisation of EML4-ALK as seen previously in overexpressed HEK293 cells.

5.There is no indication of dose response to each different ALKi, nor whether the observations occur at ALKi levels which are pharmacologically / clinically achievable or only occur at supra-pharmacologic levels. It's not clear what relation the doses used in the experimental systems have to the in vitro IC50 or what is clinically achievable in lung cancer patients. For example in the ASCEND 8 phase I clinical trial of ceritinib, the steady state pharmacokinetics at the start of cycle 2 showed a plasma level of around 1000ng/ml at all 3 doses tested (J Thorac Oncol. 2017 Sep;12(9):1357-1367. doi: 10.1016/j.jtho.2017.07.005.). Therefore repeat observations over a dose range of each ALKi would increase the potential biologic, translational and clinical relevance. I also think it's important to relate the observation made in 293 cells back to a at least one patient derived lung cancer model, (e.g. H2228) ideally more than one EML4-ALK V3 cell line (if others are available). Furthermore I think there is value in knowing whether the observed subcellular localisation with ALKi treatment occurs at concentrations in the lung cancer cell line which are known to be associated with inhibition of ALK and cytotoxicity.

The IC50 of ceritinib, alectinib and lorlatinib are 0.2 nM, 1.9 nM and 0.07 nM, respectively, in cell-free assays. The doses for the experiments were chosen according to the expression profiles of phosphorylated ALK and downstream signalling proteins as described in Marsilje et al, Sakamoto et al, and Zou et al (1, 2, 3). In our experiments, we have used the ceritinib at a 500 ng/ml dose. To further strengthen our study we examined the effect of ALK inhibitors in a dose dependent manner in MAPK and JAK/STAT signalling pathways. Additionally, we have included data from patient-derived and inducible cell lines treated with ALK inhibitors and confirmed the distinct localisation of EML4-ALK V1 and V3, as seen in HEK293 cells.

1. Marsilje, T. H., Pei, W., Chen, B., Lu, W., Uno, T., Jin, Y., Jiang, T., Kim, S., Li, N., Warmuth, M., Sarkisova, Y., Sun, F., Steffy, A., Pferdekamper, A. C., Li, A. G., Joseph, S. B., Kim, Y., Liu, B., Tuntland, T., Cui, X., Gray, N. S., Steensma, R., Wan, Y., Jiang, J., Chopiuk, G., Li, J., Gordon, W. P., Richmond, W., Johnson, K., Chang, J., Groessl, T., He, Y. Q., Phimister, A., Aycinena, A., Lee, C. C., Bursulaya, B., Karanewsky, D. S., Seidel, H. M., Harris, J. L. & Michellys, P. Y. 2013. Synthesis, Structure-Activity Relationships, And In Vivo Efficacy Of The Novel Potent And Selective Anaplastic Lymphoma Kinase (Alk) Inhibitor 5-Chloro-N2-(2-Isopropoxy-5-Methyl-4-(Piperidin-4-Yl)Phenyl)-N4-(2-(Isopropylsulfonyl)Phenyl)Pyrimidine-2,4-Diamine (Ldk378) Currently In Phase 1 And Phase 2 Clinical Trials. *J Med Chem,* 56**,** 5675-90.
2. Sakamoto, H., Tsukaguchi, T., Hiroshima, S., Kodama, T., Kobayashi, T., Fukami, T. A., Oikawa, N., Tsukuda, T., Ishii, N. & Aoki, Y. 2011. Ch5424802, A Selective Alk Inhibitor Capable Of Blocking The Resistant Gatekeeper Mutant. *Cancer Cell,* 19**,** 679-90.
3. Zou, H. Y., Li, Q., Engstrom, L. D., West, M., Appleman, V., Wong, K. A., Mctigue, M., Deng, Y. L., Liu, W., Brooun, A., Timofeevski, S., Mcdonnell, S. R., Jiang, P., Falk, M. D., Lappin, P. B., Affolter, T., Nichols, T., Hu, W., Lam, J., Johnson, T. W., Smeal, T., Charest, A. & Fantin, V. R. 2015. Pf-06463922 Is A Potent And Selective Next-Generation Ros1/Alk Inhibitor Capable Of Blocking Crizotinib-Resistant Ros1 Mutations. *Proc Natl Acad Sci U S A,* 112**,** 3493-8.

6.The introduction presents the concept that the EML4 ALK V3 variant responds poorly to ALKi but there is no clear statement as to whether there is a common thread to ALKi resistance / response and the observations around localisation made in this submission? What are the known resistance patterns to ceritinib, alectinib &

lorlatinib and is there any relationship to the subcellular EML4-ALK V3 localisation?

In the recent years, there is an emerging need for identifying resistance patterns to ALK inhibitors. Studies including Isozaki et al, Dagogo-Jack et al, Tsuji et al and Yoda et al highlighted key ALK mutations that confer resistance after ALK inhibition with either crizotinib, ceritinib and alectinib (1, 2, 3, 4, 5). Lorlatinib, a third generation ALK inhibitor, can overcome some of the ALK resistant mutations that arise from the previous ALK inhibitors. Some resistance mechanisms identified from ALK inhibition are I) point mutations on ALK kinase itself such as G1202R, L1196M, F1174L, L1198F, I1171S; II) activation of alternative receptor tyrosine kinases such as insulin-like growth factor-1 receptor (IGF1R) and human epidermal growth factor receptor 3 (HER3). However, there has not been analysis/comparison of the response of patients with different inhibitors in the context of the different inhibitors. This is an important study to do in the future.

1. Dagogo-Jack, I., Rooney, M., Lin, J. J., Nagy, R. J., Yeap, B. Y., Hubbeling, H., Chin, E., Ackil, J., Farago, A. F., Hata, A. N., Lennerz, J. K., Gainor, J. F., Lanman, R. B. & Shaw, A. T. 2019. Treatment With Next-Generation Alk Inhibitors Fuels Plasma Alk Mutation Diversity. *Clin Cancer Res,* 25**,** 6662-6670.
2. Isozaki, H., Ichihara, E., Takigawa, N., Ohashi, K., Ochi, N., Yasugi, M., Ninomiya, T., Yamane, H., Hotta, K., Sakai, K., Matsumoto, K., Hosokawa, S., Bessho, A., Sendo, T., Tanimoto, M. & Kiura, K. 2016. Non-Small Cell Lung Cancer Cells Acquire Resistance To The Alk Inhibitor Alectinib By Activating Alternative Receptor Tyrosine Kinases. *Cancer Res,* 76**,** 1506-16.
3. Tsuji, T., Ozasa, H., Aoki, W., Aburaya, S., Yamamoto Funazo, T., Furugaki, K., Yoshimura, Y., Yamazoe, M., Ajimizu, H., Yasuda, Y., Nomizo, T., Yoshida, H., Sakamori, Y., Wake, H., Ueda, M., Kim, Y. H. & Hirai, T. 2020. Yap1 Mediates Survival Of Alk-Rearranged Lung Cancer Cells Treated With Alectinib Via Pro-Apoptotic Protein Regulation. *Nat Commun,* 11**,** 74.
4. Yoda, S., Lin, J. J., Lawrence, M. S., Burke, B. J., Friboulet, L., Langenbucher, A., Dardaei, L., Prutisto-Chang, K., Dagogo-Jack, I., Timofeevski, S., Hubbeling, H., Gainor, J. F., Ferris, L. A., Riley, A. K., Kattermann, K. E., Timonina, D., Heist, R. S., Iafrate, A. J., Benes, C. H., Lennerz, J. K., Mino-Kenudson, M., Engelman, J. A., Johnson, T. W., Hata, A. N. & Shaw, A. T. 2018. Sequential Alk Inhibitors Can Select For Lorlatinib-Resistant Compound Alk Mutations In Alk-Positive Lung Cancer. *Cancer Discov,* 8**,** 714-729.
5. Lin JJ, Zhu VW, Yoda S, Yeap BY, Schrock AB, Dagogo-Jack I, Jessop NA, Jiang GY, Le LP, Gowen K, Stephens PJ, Ross JS, Ali SM, Miller VA, Johnson ML, Lovly CM, Hata AN, Gainor JF, Iafrate AJ, Shaw AT, Ou SI. Impact of EML4-ALK Variant on Resistance Mechanisms and Clinical Outcomes in ALK-Positive Lung Cancer. J Clin Oncol. 2018 Apr 20;36(12):1199-1206. doi: 10.1200/JCO.2017.76.2294. Epub 2018 Jan 26. PMID: 29373100; PMCID: PMC5903999.

7.It's not clear what the relevance of F1175 and R1275 mutations are in the EML4-ALK V3 and lung cancer. Whilst important in neuroblastoma & useful experimentally in overexpression systems the physiologic significance to EML4-ALK V3 lung cancer is perhaps less clear. It would be nice for the authors to clarify this in the discussion. Although the F1175 & R1275 are common resistance mutations seen in neuroblastoma, are they common in EML4-ALK V3 NSCLC after treatment with ceritinib / alectinib & if so do they have relevance for subsequent treatment with lorlatinib (given that it is predominantly used in 2nd line therapy)? Are there other common resistance mutations in EML4 ALK V3?

F1174 and R1275 are key residues that frequently mutated in neuroblastoma. We choose those mutations to provide insights into the structural mechanism, not for any relevance to the specific tumour biology. We acknowledge that this wasn't clear in the original submission and so we have re-written this section. We have added a better explanation and clarification the relevance of F1174L and R1275Q mutants in EML4-ALK V3 lung cancer biology. Secondary mutations in ALK kinase including F1174L frequently seen in patients with relapse due to acquired resistance to ALK inhibitors (1). These mutations seen after treatment with crizotinib/ceritinib/alectinib. Lorlatinib indeed is used as a 2nd line therapy in patients acquired resistance to the previous ALK inhibitors (crizotinib/ceritinib/alectinib). Yes, there are some other common resistance mutations in EML4-ALK V3 such as G1202R, I1171N/S, V1180L, double G1202R/F1174L. Clarification of the different resistance mechanisms to the different inhibitors in the context of different variants is important work for the future.

1. Lin JJ, Zhu VW, Yoda S, Yeap BY, Schrock AB, Dagogo-Jack I, Jessop NA, Jiang GY, Le LP, Gowen K, Stephens PJ, Ross JS, Ali SM, Miller VA, Johnson ML, Lovly CM, Hata AN, Gainor JF, Iafrate AJ, Shaw AT, Ou SI. Impact of EML4-ALK Variant on Resistance Mechanisms and Clinical Outcomes in ALK-Positive Lung Cancer. J Clin Oncol. 2018 Apr 20;36(12):1199-1206. doi: 10.1200/JCO.2017.76.2294. Epub 2018 Jan 26. PMID: 29373100; PMCID: PMC5903999.

8.The experiments to demonstrate colocalisation with GRB2 & SOS1 are presented in figure 2E and 2G. The merged images are presented in colour but the single antibody images are in black and white. I had some trouble distinguishing how many of the lipid droplets had co-localised ALK with GRB2 / SOS1 versus droplets with only ALK, I think these figures would be clearer in colour and perhaps with more than one cell example.

In the revised version, we have extended our colocalization experiments between ALK and several other signalling proteins including GRB2, SOS1, pC-KIT^{Y721}, PI3K p85β and pPLCγ2^{Y759} in patient-derived H3122 (V1) and H2228 (V3) and in the inducible Beas2B cells. We have included the green and red individual images with zoomed areas emphasizing the colocalization of the two proteins. The revised figure 2 and supplementary S3 provide a detailed representation of the colocalization between EML4-ALK V1/V3 foci and signalling proteins. This set of data highlight the existence of those proteins in EML4-ALK foci. We also include the proximity ligation assays to show signalling-relevant, protein-protein interactions that are dependent on ALK activity (e.g EML4-ALK v3/GRB2, Fig. S4).

9.The discussion "the present work may suggest that those ALK inhibitors, including ceritinib and lorlatinib, that prevent EML4-ALK V3 phase separation might tend to increase metastatic potential in EML4-ALK V3-driven cancer by shifting the protein onto microtubules. However, we propose that patients with cancer driven by EML4-ALK V3 might exhibit a better response with less metastatic spread if treated with alectinib, since it causes the inhibited EML4-ALK V3 protein to be sequestered in cytoplasmic droplets away from the microtubule network", whilst interesting is speculative and there is no data presented in the current submission which links the in vitro experimental phenomena with any in vivo tumour behaviour behaviour or response to treatment. Without additional data I don't think this speculation can be supported by the data presented.

We have removed the above statement from the discussion as we believe its out of the scope of the current manuscript. The hypothesis of phase separation increasing metastatic potential and how ALK inhibitors respond to this phenomenon is an interesting area that we are planning to address in the future.

Reviewer #2 (Significance (Required)):

A nice body of experimental work dissecting ALK sub-cellular localisation with a proposed model to define EML4 ALK V3 subcellular localisation based on ALK activation status and exposure to different ALK inhibitors.

As it currently stands I think the work is predominantly of pre-clinical interest and that the potential translational implications haven't been fully realised in the experiments presented in the submission.

REFEREES CROSS-COMMENTING

I agree that the input from each of us raised similar issues. I also didn't have additional suggests.

I have active research interests in ALK-rearranged inflammatory myofibroblastic tumours and neuroblastoma rather than lung cancer. As a clinician-scientist I think the translational significance would be enhanced by additional work focusing on bringing the observations made using the YFP tagged over-expression system back to the patient derived lung cancer model and demonstrating that the observations hold at ALKi exposure

similar to those achievable in the clinical scenario.

Response to Reviewer #3 (Evidence, reproducibility and clarity (Required)):

This manuscript presents a recent concept of phase separation based on in vitro experiments as a mechanism by which EML4-ALK V3 exhibits poor response to ALK inhibitors compared to other variants. Sampson and colleagues demonstrated that EML4-ALK V3 forms de novo liquid droplets with the signaling proteins GRB2 and SOS1 in the cytoplasm. The authors then showed differences in the subcellular localization of EML4-ALK V3 protein upon treatment with different ALK inhibitors and suggested that alectinib may result in less metastatic spread than other ALK inhibitors by promoting sequestration of EML4-ALK V3 from the microtubule network into cytoplasmic droplets. Thus, it has suggested that alectinib may provide more effective clinical benefit in NSCLC patients with EML4-ALK V3 over other inhibitors.

The idea that the activity of ALK inhibitors against EML4-ALK V3 is associated with the distinct ability to liquid droplet formation of EML4-ALK V3 is an exciting and the imaging experiments are of high quality. However, the functional significance of the liquid droplet by EML4-ALK V3 remains unclear and though my concern is that overexpression of YFP-EML4-ALK V3 is not representative of the state in EML4-ALK V3. Overall experiments are insufficient to support the claims of authors.

We thank the referee for highlighting that the study was well performed and interesting. The functional significance of liquid-liquid phase separation (LLPS) is an emerging and exciting phenomenon that recently gained much attention. We believe it is an important area that needs further exploration and understand the functionality of molecules in a cellular content.

Major comments

1.Previous retrospective studies analyzing the correlation between the ALK variant and differential sensitivity to ALK inhibitors have suggested that variant 3 had a relatively poorer prognosis for ALK inhibitors than other variants. In this context, the authors only focused on the liquid droplet formation of EML4-ALK V3 using specific EML4-ALK V3-overexpressing cell lines. However, a previous study showed that overexpression of EML4-ALK variant 3 exhibited different localization depending on the established cell line model. Indeed, even in their study, the co-localization of GRB2 and cytoplasmic V3 droplets was less pronounced in H2228 than in isogenic Beas2B cells that inducibly expression V3.

As we mentioned above, there are several studies highlighting the poor prognosis and response to ALK inhibitors compared to EML4-ALK V1. In this revised manuscript, we investigated the localisation of EML4-ALK V1 and V3 fusion protein. We have shown foci formation of EML4-ALK V1 and V3 is various cell lines either overexpressed (HEK293) or endogenous patient-derived (H3122 (V1) and H2228(V3)) or tetracycline induced (Beas2B). Some other studies have shown that overexpression of V1 and V3 had cytoplasmic and nucleus localisation in NIH3T3 cells and microtubule binding in HeLa cells (1, 2), however their exact localisation remained elusive.

1. Heuckmann, J. M., Balke-Want, H., Malchers, F., Peifer, M., Sos, M. L., Koker, M., Meder, L., Lovly, C. M., Heukamp, L. C., Pao, W., Küppers, R. & Thomas, R. K. 2012. Differential Protein Stability And Alk Inhibitor Sensitivity Of Eml4-Alk Fusion Variants. *Clin Cancer Res,* 18**,** 4682-90.

2. Richards, M. W., O'regan, L., Roth, D., Montgomery, J. M., Straube, A., Fry, A. M. & Bayliss, R. 2015. Microtubule Association Of Eml Proteins And The Eml4-Alk Variant 3 Oncoprotein Require An N-Terminal Trimerization Domain. *Biochem J,* 467**,** 529-36.

1-1.To support their claim that the ability of EML4-ALK V3 to form liquid droplets and the different effects of ALK inhibitors on these properties are related to drug sensitivity, the authors should present the results of EML4-ALK V1 (H3122) as an appropriate control for all their experiments.

In the revised manuscript, we have included data of the EML4-ALK V1 protein from patient-derived (H3122), overexpressed HEK293 and inducible Beas2B. The appropriate controls were included in each set of data. We found that EML4-ALK V1 cells form cytoplasmic foci as seen in V3 cells. These data confirm findings from recent papers of Tupule et al and Qin et al (1,2).

1. Tulpule, A., Guan, J., Neel, D. S., Allegakoen, H. R., Lin, Y. P., Brown, D., Chou, Y. T., Heslin, A., Chatterjee, N., Perati, S., Menon, S., Nguyen, T. A., Debnath, J., Ramirez, A. D., Shi, X., Yang, B., Feng, S., Makhija, S., Huang, B. and Bivona, T. G. (2021) 'Kinase-mediated RAS signaling via membraneless cytoplasmic protein granules', *Cell,* 184(10), pp. 2649-2664.e18.

2. Qin, Z., Sun, H., Yue, M. *et al.* Phase separation of EML4–ALK in firing downstream signaling and promoting lung tumorigenesis. *Cell Discov* **7,** 33 (2021). https://doi.org/10.1038/s41421-021-00270-5

1-2. In line with the results for the different localization of EML4-ALK V3 to various inhibitors, it would further support their claims if the authors show in parallel the effects of inhibitors on cell viability and downstream signals such as AKT, ERK and STAT3 in recombinant model cell lines expressing V3.

We have included a detailed set of data from western blotting analysis that investigate the expression profiles of downstream signalling proteins such as cleave AKT, ERK and STAT3 in patient-derived EML4-ALK V1 and V3 cell lines. Data suggested that inhibition of EML4-ALK V1 by ALK inhibitors resulted in a dose-dependent loss of activated ALK, ERK, STAT and AKT. Similarly, inhibition of EML4-ALK V3 by ALK inhibitors resulted in a significant loss of STAT3 but not AKT and ERK. These results suggest that dynamic foci of H2228 (V3) cells may be less sensitive to ALK inhibition than the static foci of H3122 (V1) cells.

2.The discussion is poor. The results should be discussed in a more data of the literature and with respect to clinical of view particularly on the links between de novo resistant and metastasis in EML4-ALK V3.

We revised the discussion to focus on the key findings: the molecular mechanism of foci formation and relationship to ALK activity/conformation; the contribution of foci to signalling; the differences between V1 and V3, and how we can develop a strategy to disrupt foci in V3 patients.

Reviewer #3 (Significance (Required)):

Although the functional mechanism is still unclear, analysis of relationship between the phase-separation properties of EML4-ALK V3 and its sensitivity to targeted therapy suggests a conceptual advance in de novo resistance of EML4-ALK V3.
I am not an expert in the field of new liquid phase separations, but the theoretical interpretation that localization of oncogenic fusion proteins could contribute to the response of the targeted therapy was very interesting.
It is also expected to further expand the exploration of drug resistance by providing initial evidence of novel mechanisms for targeted therapeutic strategies.

REFEREES CROSS-COMMENTING

My opinions with other reviewers are generally in line.
So there are no additional comments.

Referee #2:

In reading the current revision I have been left with the overall impression that the authors really haven't taken on board comments and suggestions from the prior review & nor incorporated them into the revised submission.

I would return to the major comment that I had had previously made:

"The majority of the data has been generated using overexpression of tagged EML4-ALK V3 (including mutants and deletion constructs) in 293 cells. There is supporting data from one patient derived EML4 ALK V3 cell line and tetracycline induced expression in a lung cell model. The strength of the observations made & and conclusions drawn from the tagged EML4-ALK V3 constructs would be increased by additional work with the H2228 cell line and/or additional patient derived lung cancer cell lines."

In the original review, this comment also included the phrase "(these may be difficult to come by)". Indeed, this is most certainly the case and there are no additional EML4-ALK cell lines that we can access beyond the H2228 and H3122. This is a major issue for the field, and we are setting up to generate novel patient-derived cell lines, but progress on this will be very slow, not least because our clinical colleagues on the NHS are fully occupied and taking samples from lung cancer patients for cell-line generation is not a standard practice. We require new ethical approval and to establish the pipeline.

The current submission relies heavily on model systems & whilst the experiments are elegantly performed there are many instances where supporting & corroborating data in additional cells has not been presented in this revision. The strength of the experimental observations needs to be validated in additional cell systems & models. There is a very strong reliance on overexpression constructs (YFP tagged constructs & inducible expression) & model systems (predominantly 293 and Beas2B cells) with only a single lung cancer cell line. So despite the revised submission I am left, as before, "without a real sense of the physiologic, pharmacologic and translational relevance of the findings."

The key data establishing the phase separation and the presence of signalling molecules in the foci was done in patient-derived cell lines H2228 and H3122 (Figs 1&2). The model cell systems are used to enable live-cell imaging of tagged EML4-ALK and for the structure-function studies, such as through expression of kinase-dead EML4-ALK (and other mutations).

In reading the revision and authors response to the reviewers comments I am left with the impression that few actual changes have been made…

This is an incorrect statement – the revised paper includes a parallel set of studies in the EML4-ALK V1 cell line H3122 (Figure 1; 2; 3) –the use of hexanediol to probe the type of phase separation (Figure 2; S2); extensive characterisation of the signalling in the V1 and V3 cells (Figure 2, Figures S2-S5); structure-function studies that identify the key region within EML4-ALK V1 that confers its less dynamic behaviour (Fig. 4). The revised paper comprises 8 Figures and 9 Supplemental Figures (up from 6 and 4, respectively), and was extensively re-written. This is not a "few actual changes".

… and that there continue to be statements within the discussion that are unsupported by the experimental data and in my opinion cannot be supported, even with citing the work of others, specifically: "indeed the present work may suggest that those ALK inhibitors, including ceritinib and lorlatinib, that prevent EML4-ALK V3 phase separation might tend to increase metastatic potential in EML4-ALK V3-driven cancer by shifting the protein onto microtubules. However, we propose that patients with cancer driven by EML4-ALK V3 might exhibit a better response with less metastatic spread if treated with alectinib, since it causes the inhibited EML4-ALK V3 protein to be sequestered in cytoplasmic droplets away from the microtubule network"

The quoted section was removed, and it is unreasonable for the reviewer to criticize us on the basis of text that was not present in the revised manuscript.

Whilst a fundamentally interesting observation has been made, I think the real importance and relevance of this finding hasn't been unravelled but would require additional work in a broader range of experimental systems.

We maintain that our work is an important advance in the characterisation of phase separation in EML4-ALK lung cancer – we both characterise the phase separation and signalling in patient cell lines, and provide a structure-function relationship between the ‘type’ of phase separation and the molecular interactions that occur in the two most common EML4-ALK variants.

Prof. Richard Bayliss
University of Leeds
School of Molecular and Cellular Biology
Woodhouse Lane
Leeds LS2 9JT
United Kingdom

Dear Prof. Bayliss,

I am very pleased to accept your manuscript for publication in the next available issue of EMBO reports. Thank you for your contribution to our journal.

At the end of this email I include important information about how to proceed. Please ensure that you take the time to read the information and complete and return the necessary forms to allow us to publish your manuscript as quickly as possible.

As part of the EMBO publication's Transparent Editorial Process, EMBO reports publishes online a Review Process File to accompany accepted manuscripts. As you are aware, this File will be published in conjunction with your paper and will include the referee reports, your point-by-point response and all pertinent correspondence relating to the manuscript.

If you do NOT want this File to be published, please inform the editorial office within 2 days, if you have not done so already, otherwise the File will be published by default [contact: emboreports@embo.org]. If you do opt out, the Review Process File link will point to the following statement: "No Review Process File is available with this article, as the authors have chosen not to make the review process public in this case."

Should you be planning a Press Release on your article, please get in contact with emboreports@wiley.com as early as possible, in order to coordinate publication and release dates.

Thank you again for your contribution to EMBO reports and congratulations on a successful publication. Please consider us again in the future for your most exciting work.

Yours sincerely,

Achim Breiling
Editor
EMBO Reports

****

THINGS TO DO NOW:

You will receive proofs by e-mail approximately 2-3 weeks after all relevant files have been sent to

our Production Office; you should return your corrections within 2 days of receiving the proofs.

Please inform us if there is likely to be any difficulty in reaching you at the above address at that time. Failure to meet our deadlines may result in a delay of publication, or publication without your corrections.

All further communications concerning your paper should quote reference number EMBOR-2021-53693V2 and be addressed to emboreports@wiley.com.

Should you be planning a Press Release on your article, please get in contact with emboreports@wiley.com as early as possible, in order to coordinate publication and release dates.
